# Neddylation Promotes Ubiquitylation and Release of Ku from DNA-Damage Sites

**DOI:** 10.1016/j.celrep.2015.03.058

**Published:** 2015-04-23

**Authors:** Jessica S. Brown, Natalia Lukashchuk, Matylda Sczaniecka-Clift, Sébastien Britton, Carlos le Sage, Patrick Calsou, Petra Beli, Yaron Galanty, Stephen P. Jackson

**Affiliations:** 1 The Wellcome Trust and Cancer Research UK Gurdon Institute, University of Cambridge, Cambridge 2 1QN, UK; 2 Institut de Pharmacologie et de Biologie Structurale, CNRS, Université de Toulouse-Université Paul Sabatier, Equipe Labellisée Ligue contre le Cancer, 31077 Toulouse, France; 3 Institute of Molecular Biology (IMB), 55128 Mainz, Germany

## Abstract

The activities of many DNA-repair proteins are controlled through reversible covalent modification by ubiquitin and ubiquitin-like molecules. Nonhomologous end-joining (NHEJ) is the predominant DNA double-strand break (DSB) repair pathway in mammalian cells and is initiated by DSB ends being recognized by the Ku70/Ku80 (Ku) heterodimer. By using MLN4924, an anti-cancer drug in clinical trials that specifically inhibits conjugation of the ubiquitin-like protein, NEDD8, to target proteins, we demonstrate that NEDD8 accumulation at DNA-damage sites is a highly dynamic process. In addition, we show that depleting cells of the NEDD8 E2-conjugating enzyme, UBE2M, yields ionizing radiation hypersensitivity and reduced cell survival following NHEJ. Finally, we demonstrate that neddylation promotes Ku ubiquitylation after DNA damage and release of Ku and Ku-associated proteins from damage sites following repair. These studies provide insights into how the NHEJ core complex dissociates from repair sites and highlight its importance for cell survival following DSB induction.

## Highlights

• NEDD8 accumulation at DNA-damage sites is a dynamic process
• Depletion of the NEDD8 E2 UBE2M reduces cell survival following NHEJ
• Neddylation promotes ubiquitylation of Ku following DNA damage
• Neddylation promotes Ku release from damage sites following DNA repair

## Graphical Abstract

**Figure:**
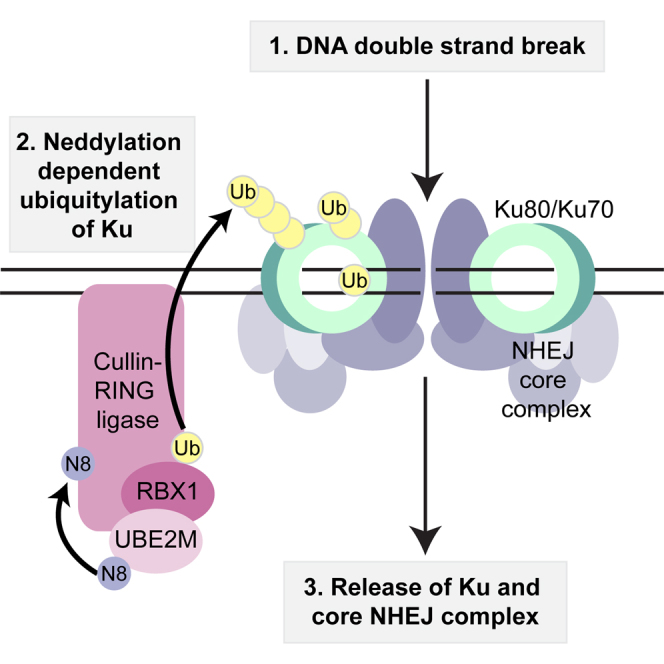

## Teaser

Double-strand break (DSB) repair is essential for genomic stability and is tightly regulated by modification of proteins with ubiquitin and ubiquitin-like molecules. Brown et al. show that, upon completion of repair, neddylation promotes release of the main DSB sensor Ku from damaged DNA, providing insight into a long-sought mechanism.

## Introduction

The DNA-damage response (DDR), comprising the sensing, signaling, and repair of damaged DNA, requires recruitment and post-translational modification (PTM) of many proteins at DNA-damage sites (Polo and Jackson, 2011). Effective DSB repair is essential for genomic stability, with hereditary DSB repair defects causing cancer predisposition, immunodeficiency, developmental defects, and hypersensitivity to DNA damaging agents (Jackson and Bartek, 2009; Ciccia and Elledge, 2010). DSB repair mainly occurs through two pathways: homologous recombination (HR) and nonhomologous end-joining (NHEJ). Classical NHEJ requires binding of the Ku70/Ku80 heterodimer to DNA ends, with ensuing recruitment of DNA-PKcs, PAXX, and end-processing factors leading to repair by the DNA ligase IV/XRCC4/XLF complex (Davis and Chen, 2013; Grundy et al., 2014; Wang and Lees-Miller, 2013; Ochi et al., 2015; Xing et al., 2015). While the main NHEJ proteins have been characterized, it is not yet clear how their recruitment to, and dissociation from, DSBs is regulated.

**Figure 1:**
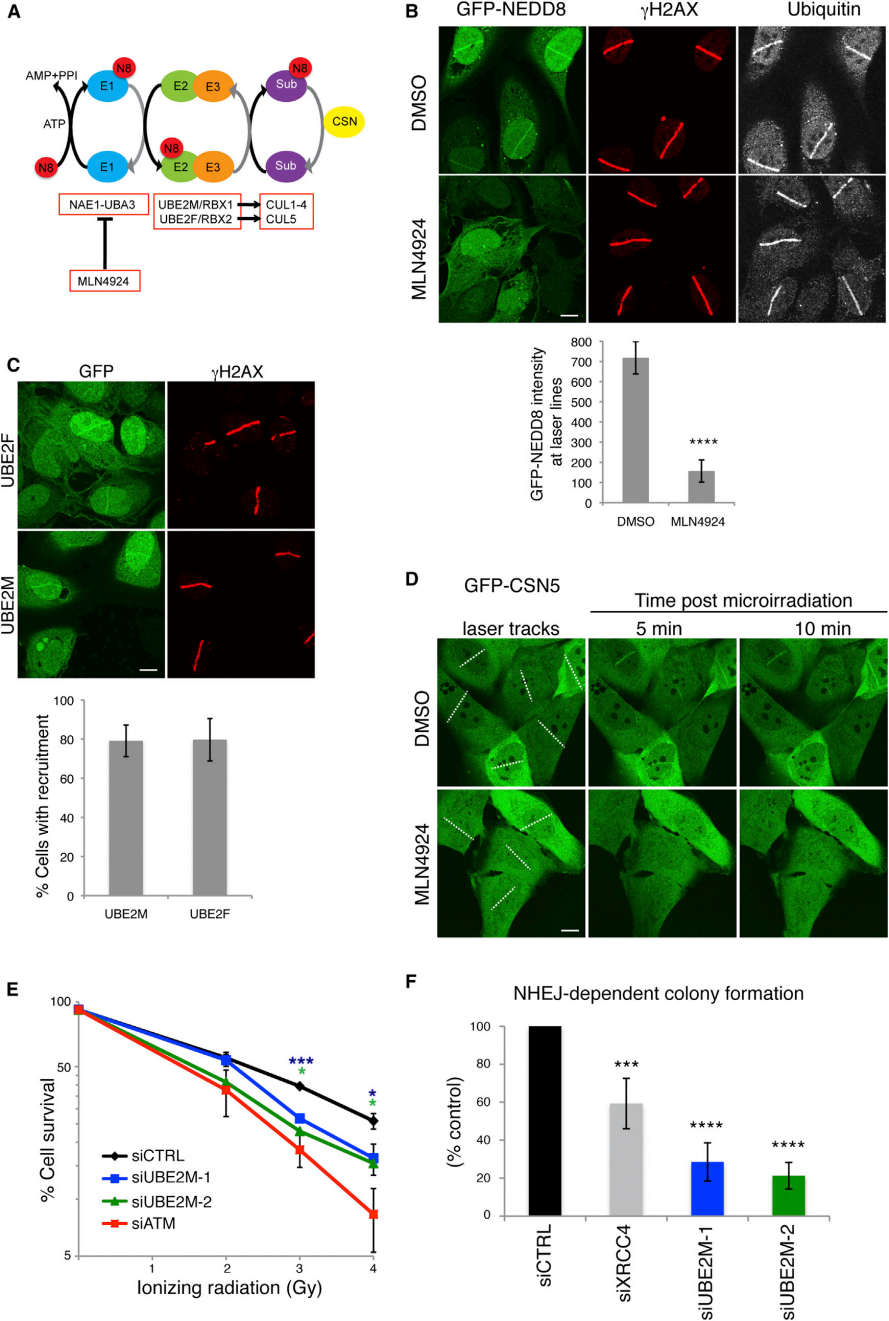
NEDD8 and the Neddylation Machinery Accumulate at Sites of DNA Breaks and Promote Cell Survival after NHEJ (A) Representation of major neddylation pathway components. NEDD8 (N8) is conjugated in an ATP-dependent cascade involving an E1 (NAE1-UBA3), E2 (UBE2M or F), and E3 (RBX1 or 2) to Cullin substrates (Sub). Neddylation is reversed by the CSN complex. MLN4924 inhibits UBA3. Figure adapted from Brown and Jackson (2015). (B) MLN4924 blocks NEDD8, but not ubiquitin recruitment to DNA-damage sites. U2OS-GFP-NEDD8 cells were pre-treated for 1 hr with DMSO or 3 μM MLN4924 and laser microirradiated. Cells were fixed after 20 min and visualized by immunofluorescence as indicated. Graph shows average intensity of GFP-NEDD8 at the laser line from three experiments ±SD. White bar represents 10 μM. Asterisks indicate statistically significant difference to control (^∗^p < 0.05; ^∗∗^p < 0.01; ^∗∗∗^p < 0.001; ^∗∗∗∗^p ≤ 0.0001). (C) GFP-UBE2F and GFP-UBE2M are recruited to DNA-damage sites. U2OS cells stably expressing GFP-UBE2F or GFP-UBE2M were laser microirradiated, fixed, and visualized as in (B). Graph shows average percentage of γH2AX positive cells with detectable GFP-UBE2M or GFP-UBE2F recruitment from five independent experiments ±SD. White bar represents 10 μM. (D) GFP-CSN5 recruitment to DNA-damage sites is blocked by MLN4924. U2OS cells stably expressing GFP-CSN5 were treated as in (B). Images were acquired by live cell imaging. Laser tracks are indicated by dashed white lines. White bar represents 10 μM (E) UBE2M depletion causes hypersensitivity to IR. Clonogenic U2OS cell survivals were performed after transfection with indicated siRNAs and doses of IR. Each point represents an average of at least three independent experiments (except UBE2M-2 which was repeated twice). Error bars correspond to SDs, and asterisks are as in (B). (F) UBE2M depletion causes an NHEJ defect. Random plasmid integration assay was performed in U2OS cells transfected with indicated siRNAs. Error bars correspond to SD of at least three independent experiments (asterisks as in B). See also Figure S1.

The covalent attachments of ubiquitin and the ubiquitin-like molecule (UBL) SUMO to DDR proteins have well-established roles in the DDR (Jackson and Durocher, 2013). However, functions of other UBLs in such processes remain relatively unexplored (Pinder et al., 2013). Of the UBLs, NEDD8 has the highest sequence similarity to ubiquitin and is conjugated to substrates in an enzymatic process analogous to those of ubiquitin and other UBLs (Figure 1A; reviewed by Enchev et al., 2015; Lydeard et al., 2013; Schulman and Harper, 2009; Watson et al., 2011). The NEDD8 E1 activating enzyme, comprising the NAE1-UBA3 heterodimer, adenylates the exposed NEDD8 C-terminal glycine and forms a covalent NEDD8-thioester linkage. Activated NEDD8 is then conjugated to substrates, predominantly by the E2/E3 enzyme complexes UBE2M/RBX1 or UBE2F/RBX2 (Huang et al., 2009). Although RBX1 and RBX2 are the major NEDD8 E3s, others have been described (Kurz et al., 2005; Ma et al., 2013; Meyer-Schaller et al., 2009; Kurz et al., 2008; Scott et al., 2010; Xirodimas et al., 2004). De-neddylation is mainly mediated by the CSN (COP9 signalosome) complex (Cope et al., 2002). The best-characterized NEDD8 substrates, cullins (CUL1, 2, 3, 4A, 4B, 5, and 7 and PARC in human cells), serve as molecular scaffolds for cullin-RING ubiquitin ligases (CRLs; Lydeard et al., 2013; Sarikas et al., 2011). Cullin neddylation increases CRL ubiquitylation activity via conformational changes that optimize ubiquitin transfer to target proteins (Duda et al., 2008). MLN4924, a mechanism-based inhibitor of NAE1-UBA3, currently being explored as an anti-cancer treatment, blocks neddylation in cells, inhibiting CRL activity (Brownell et al., 2010; Soucy et al., 2009; Milhollen et al., 2011). While neddylation has a well-defined role in DNA nucleotide excision repair (Groisman et al., 2003), recent studies have connected it to DSB-repair processes (Cukras et al., 2014; Li et al., 2014; Ma et al., 2013; Wu et al., 2012; Jimeno et al., 2015). Here, we establish that neddylation is crucial for cell survival after DSB induction, and that it promotes Ku ubiquitylation and release from DSB sites.

## Results

### Neddylation Occurs at DSB Sites

To determine whether NEDD8 is present at DNA-damage sites, we used laser microirradiation to generate DSBs in cells pre-sensitized with bromodeoxyuridine (BrdU; Lukas et al., 2003). This revealed that both stably expressed GFP-tagged (Figure 1B) and endogenous (Figure S1A) NEDD8 were detectable at DNA-damage sites within minutes, co-localizing with Ser-139 phosphorylated histone H2AX (γH2AX), an established DSB marker (Rogakou et al., 1998). Pre-incubating cells for 1 hr with MLN4924 at a dose that effectively inhibits NEDD8 conjugation in cells (Figure S1B) blocked NEDD8 recruitment to sites of laser microirradiation (Figures 1B and S1A), indicating that DNA-damage-induced NEDD8 accrual requires an active neddylation pathway.

The ubiquitin machinery, particularly the ubiquitin E1 UBE1, can utilize highly overexpressed NEDD8, causing “false” neddylation of substrates (Hjerpe et al., 2012). Importantly, GFP-NEDD8 conjugation detected by immunoblotting of extracts from our stable cell line was blocked by MLN4924, but not by depleting UBE1 (Figure S1C). This indicated that overexpressed GFP-NEDD8 in this cell line was not substantially used by the ubiquitin system. However, depletion of UBE1 did reduce GFP-NEDD8 recruitment to DNA-damage sites (Figure S1D), although to a lesser extent than MLN4924 treatment (Figures 1B and S1D), demonstrating that NEDD8 accumulation is at least partially dependent on ubiquitylation-mediated events. Of note, NEDD8 accumulation was only observed in BrdU pretreated cells (Figure S1E), implying that in our system, NEDD8 accrual was largely promoted by DSBs rather than other forms of damage (Lukas et al., 2003). NEDD8 recruitment did not require the activity of PARP or the DDR kinases ATM, ATR, and DNA-PK (Figure S1F). Indeed, impairing DNA repair by inhibiting these kinases actually increased NEDD8 accumulation at laser sites (Figure S1F), supporting a role for neddylation in DSB-dependent events.

Although it was reported recently by Ma et al. (2013) that neddylation promotes ubiquitylation at sites of DNA damage, we found that, in our system, robust inhibition of neddylation by MLN4924 did not decrease ubiquitylation at DNA-damage sites as detected by the FK2 antibody (Figure 1B). In the Ma et al. (2013) study, neddylation was inhibited by depleting RNF111/Arkadia, which they reported to be a NEDD8 E3 ligase. However, RNF111 is also a well-established ubiquitin E3 ligase with a role in the DDR (Poulsen et al., 2013) and it was not determined by Ma et al. whether the effects they observed on ubiquitylation and other aspects of the DDR were due to the ubiquitin E3 activity, rather than the reported NEDD8 E3 ligase activity of RNF111.

Through assessing GFP-NEDD8 recruitment kinetics in live cells, we found that NEDD8 accumulated at damaged sites as early as 5 min after microirradiation and persisted until 40 min in most cells (Figure S1G, left). To further investigate neddylation dynamics, we treated cells with MLN4924 immediately before laser microirradiation. In these cells, NEDD8 was initially detected at damaged sites (5 min) and then rapidly disappeared, being undetectable by 15 min (Figure S1G, right). The initial accumulation of NEDD8 in this instance most likely represents the time taken for neddylation to be completely inhibited in cells by MLN4924 (which occurs within 5 min; Brownell et al., 2010). These data therefore suggested that neddylation is a dynamic modification that occurs and turns over at DSBs, although we cannot exclude the possibility that some pre-neddylated proteins accumulate at damaged sites then disperse. To corroborate our findings, we tested for DNA-damage-dependent recruitment of neddylation-pathway components. Crucially, this revealed that the NEDD8-conjugating E2 enzymes UBE2M and UBE2F (Figure 1C), and the deneddylating-complex catalytic subunit, CSN5 (Figure 1D), were recruited to DNA-damage sites with kinetics similar to that of GFP-NEDD8. Furthermore, CSN5 recruitment was blocked by MLN4924 (Figure 1D), implying that neddylation is required for CSN5 recruitment. Collectively, these data strongly supported a model in which neddylation and deneddylation actively occur at DSB sites.

### Neddylation Promotes Cell Survival after NHEJ

In light of the above findings and because inhibiting neddylation can sensitize cells to DNA-damaging agents (Kee et al., 2012; Wei et al., 2012; Yang et al., 2012; Garcia et al., 2014), we hypothesized that neddylation promotes DSB repair. To investigate this, we tested the effects of depleting UBE2M or UBE2F by small interfering RNAs (siRNAs) on clonogenic cell survival following ionizing radiation (IR) treatment. Notably, while both UBE2M and UBE2F were recruited to DNA-damage sites (Figure 1C), UBE2M but not UBE2F depletion significantly sensitized cells to IR (Figures 1E and S1H). We speculate that functional compensation by UBE2M (and potentially lower levels of UBE2F compared to UBE2M in the cells we tested) may explain why UBE2F was recruited to laser lines but its depletion did not sensitize cells to IR.

Although DSB repair by HR is restricted to S and G2 cells and can take several hours to complete (Shibata et al., 2011), NHEJ occurs in all cell-cycle stages, with most simple breaks being repaired within minutes (Wang et al., 2001; DiBiase et al., 2000). Because NEDD8 accrual at DNA-damage sites was rapid and occurred in most cells, we speculated that neddylation might regulate NHEJ. In accord with this, depleting UBE2M with two independent siRNAs significantly reduced the number of cell colonies arising in an assay for random plasmid integration (Figure 1F), which is mediated by NHEJ as well as alternative DNA end-joining processes.

### Neddylation Promotes Ku Release from DNA Damage Sites

**Figure 2:**
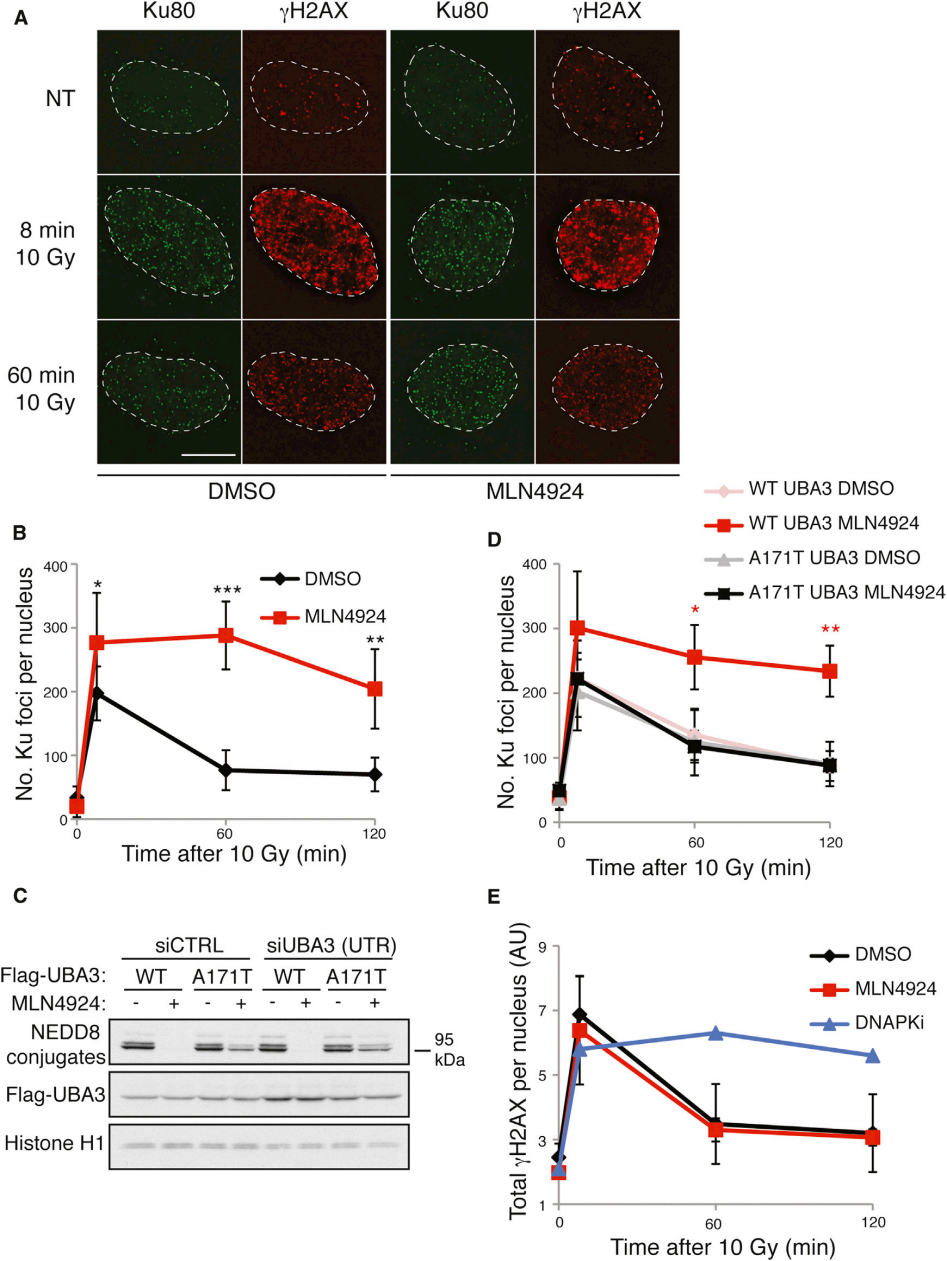
MLN4924 Inhibits Ku Removal from DNA Repair Sites (A and B) MLN4924 causes Ku80 foci persistence after IR. U2OS cells were pre-treated with DMSO or 3 μM MLN4924 for 1 hr and then subjected to 10 Gy IR. Samples were pre-extracted with CSK+RNase A and visualized by immunofluorescence. (A) shows representative images, and (B) shows quantification. Dotted lines indicate nuclear peripheries. Error bars correspond to SDs of at least three independent experiments (asterisks as in Figure 1B). White bar represents 10 μM. (C) U2OS-A171T UBA3 are resistant to MLN4924. U2OS cells stably expressing WT UBA3 or A171T UBA3 were treated with DMSO or 3 μM MLN4924 for 1 hr and analyzed by immunoblotting with indicated antibodies. Endogenous UBA3 was depleted with a siRNA to the 3′ UTR (Figure S2B). Neddylated conjugates are detected with a NEDD8-specific antibody. (D) MLN4924 effects on Ku80 foci retention are through UBA3 inhibition. U2OS cells stably expressing WT UBA3 or A171T UBA3 were processed as in (A), and results were quantified as in (B). (E) MLN4924 does not affect γH2AX recovery after IR. Quantification of total γH2AX intensity per nucleus in cells treated with 10 Gy IR then harvested at indicated times. Samples were prepared as in (A). Pre-treatment for 1 hr with 3 μM DNA-PK inhibitor (DNA-PKi) used as a positive control. Statistical analysis as in (B). AU, arbitrary units. See also Figure S2.

To explore the impact of neddylation on NHEJ, we used high-resolution microscopy together with an RNase A-based extraction method to study formation and dissolution of Ku IR-induced foci (IRIF). In agreement with published findings (Britton et al., 2013), Ku foci in control cells were formed within 8 min following IR and then decayed over time, returning to near baseline levels by 1 hr (Figures 2A and 2B). Strikingly, while not impairing Ku IRIF formation, MLN4924 treatment significantly delayed their dissolution, with high numbers of Ku foci remaining even after 2 hr (Figures 2A and 2B). This effect was not through MLN4924 itself causing DNA damage because parallel treatments of non-irradiated cells with MLN4924 did not induce Ku IRIF or γH2AX (Figure S2A; MLN4924 treatment for longer than 6 hr did cause DNA damage as previously described by Soucy et al., 2009). Of note, while Feng and Chen (2012) published that RNF8 depletion caused Ku80 retention at laser microirradiation sites, we were unable to detect any effect of RNF8 depletion on the resolution of Ku IRIF (data not shown).

To test whether the effect of MLN4924 on Ku removal was indeed via UBA3 inhibition, we generated U2OS cell lines stably expressing wild-type UBA3 or a UBA3 Ala-171 to Thr mutant (UBA3-A171T) that confers MLN4924 resistance (Toth et al., 2012; Milhollen et al., 2012). As expected, NEDD8 conjugation was abolished by MLN4924 in cells expressing wild-type UBA3 but not UBA3 A171T (Figures 2C and S2B). Importantly, while both cell lines showed comparable Ku IRIF kinetics under control conditions (Figure 2D), MLN4924 caused persistent Ku IRIF only in cells expressing wild-type UBA3 (Figure 2D), thus indicating that the effect of MLN4924 on Ku was via UBA3 inhibition. Although we initially considered the possibility that Ku IRIF persistence reflected defective DSB repair, this did not appear to be the case because MLN4924 did not impair the time-dependent reduction of IR-induced γH2AX, a well-established readout of DSB repair (Britton et al., 2013; Löbrich et al., 2010), detected either by immunoblotting (Figure S2C) or immunofluorescence microscopy (Figures 2A and 2E; note in Figure 2E that γH2AX did persist following DNA-PK inhibition). During these analyses, we found that the size and intensity of Ku foci were unaffected by MLN4924, indicating that MLN4924 does not lead to more Ku molecules being loaded onto each DSB (data not shown). Collectively, these findings suggested that blocking neddylation does not affect Ku loading but rather impairs Ku removal from damage sites after repair has occurred.

**Figure 3:**
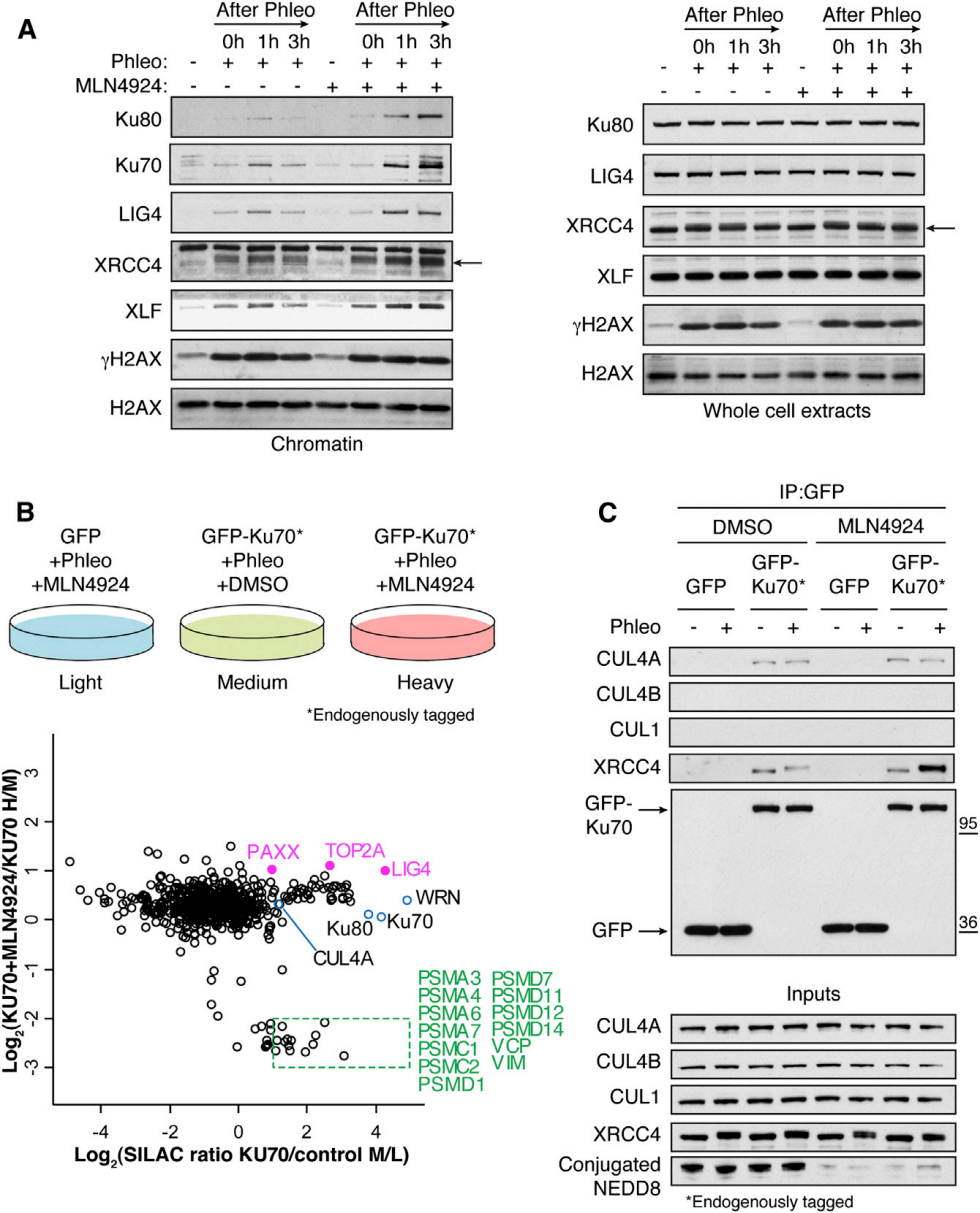
Proteomics Identifies Neddylation-Dependent Ku Interactors (A) MLN4924 causes retention of NHEJ factors on the chromatin. U2OS cells were pretreated with DMSO or 3 μM MLN4924 for 1 hr and then treated with 500 μM phleomycin (Phleo) for 1 hr. Cells were left to recover in the presence of MLN4924 or DMSO following phleomycin removal and then collected at the indicated times. Cells were pre-extracted with CSK buffer + RNase A prior to lysis (chromatin; left) or lysed as whole cell extracts (right) and immunoblotted with indicated antibodies. Black arrow indicates XRCC4. (B) RPE-1 cells stably expressing GFP or Ku70 endogenously tagged with GFP were labeled with light, medium, or heavy isotopes and treated as indicated. Cell lysates were subjected to GFP retrieval. Enriched proteins were resolved by SDS-PAGE and proteolysed in gel with trypsin, and peptides were analyzed by LC-MS/MS. The scatterplot shows the logarithmized SILAC ratio of GFP-KU70/GFP control and GFP-KU70 + MLN4924/GFP-KU70. The known Ku interactors and CUL4A (ratio 2.23) are labeled in black font and open blue circles. In pink are interactions enhanced upon MLN4924. In green are interactions decreased upon MLN4924 (see also Table S1). (C) Experiment repeated as in (B) without isotope labeling of cells. Following GFP IP, cell lysates were immunoblotted with indicated antibodies. Note that CUL1 (Postow and Funabiki, 2013) and CUL4B were not detected in Ku immunoprecipitates. See also Figure S3.

To assess the above model by a different approach, we used immunoblotting to monitor the accumulation of Ku and other NHEJ factors in RNase A-resistant chromatin fractions after treating cells with the radiomimetic compound phleomycin. In accord with our immunofluorescence data, inhibiting neddylation with MLN4924 caused Ku80 and Ku70 persistence on chromatin after treating cells with a pulse of phleomycin (Figure 3A). Similarly, MLN4924 caused persistence of the NHEJ factors XRCC4, LIG4, and XLF, suggesting that they are recruited and subsequently released concomitantly with Ku (Figure 3A; as shown on the right, total levels of Ku80, XRCC4, LIG4, and XLF were unaltered by DNA damage and/or MLN4924). These data supported a model in which neddylation promotes removal of Ku and other NHEJ factors from DNA-damage sites.

### Proteomics Identifies Neddylation-Dependent Ku Interactors

To identify factors that might associate with Ku in a NEDD8-pathway-dependent manner, we used human RPE-1 cells stably expressing GFP or RPE-GFP-Ku70 cells expressing endogenously tagged GFP-Ku70, wherein the GFP-tag was fused to one of the *XRCC6* chromosomal alleles (Britton et al., 2013), in SILAC (stable isotope labeling of amino acids in cell culture) studies followed by liquid chromatography-tandem mass spectrometry (LC-MS/MS; Figure 3B; Table S1). Applying a cutoff of ≥2-fold enrichment for Ku-specific binding, we identified several known Ku interactors as well as various other proteins, including CUL4A (Figure 3B; a highly related cullin CUL4B had a ratio of 1.8). Subsequent reciprocal co-immunoprecipitation studies confirmed CUL4A as a Ku interactor (Figures 3C and S3A). Notably, we found that depletion of either CUL4A or CUL4B significantly reduced NEDD8 accrual at DNA-damage sites (Figure S3B), and stably expressed GFP-CUL4A and GFP-CUL4B were both recruited to DSB sites (Figure S3C). To investigate their potential functional roles in Ku release from chromatin following DNA repair, we depleted CUL4A/CUL4B by siRNA and established cell lines stably expressing inducible dominant-negative CUL4A or CUL4B. However, by neither of these approaches were we able to demonstrate consistently strong effects on Ku removal (data not shown). Nevertheless, we also noted that neither approach inhibited CRL4 ubiquitylation activity to a level comparable to MLN4924 treatment, as monitored by protein levels of the CRL4 substrates CDT1, p27, and p21 (Figures S3D and S3E). We therefore concluded that residual CRL enzymatic activity and/or functional redundancy between CUL4A, CUL4B, and probably other cullins likely precluded us from observing effects in these studies. Indeed, in vitro studies have implicated CUL1 in the removal of Ku from DNA in cell-free *Xenopus laevis* egg extracts (Postow et al., 2008; Postow and Funabiki, 2013), thus indirectly supporting the idea that Ku could be a shared substrate of CUL1 and CUL4A/B in human cells. In regard to the above, we found that depletion of RBX1—which functions together with UBE2M (Huang et al., 2009) and is the NEDD8 and ubiquitin E3 ligase for cullins 1, 2, 3, 4A, and 4B—increased the number of Ku IRIF at all time points tested and caused persistence of Ku and NHEJ factors on chromatin after phleomycin treatment (Figures S3F–S3H). These effects on NHEJ-factor kinetics were less marked than with MLN4924 treatment, however, and also the kinetics of Ku release following RBX1 depletion differed from that seen with MLN4924 treatment. These differences might reflect the potency of MLN4924 treatment compared to incomplete RBX1 depletion (Figure S3H) and/or could be influenced by prolonged RBX1 depletion over 72 hr compared to 1 hr exposure to MLN4924. Collectively, these data were consistent with CRL activity promoting Ku removal, although we acknowledge that other factors might also be involved.

In support of a model in which neddylation promotes dissociation of the NHEJ apparatus (Figure 3A), our proteomics data and subsequent co-immunoprecipitation studies revealed that the interaction between Ku and DNA ligase 4/XRCC4, as well as the recently identified NHEJ complex component PAXX (Ochi et al., 2015; Xing et al., 2015), was significantly enhanced when neddylation was blocked by MLN4924 (Figures 3B and 3C; Table S1). Interestingly, the interaction between Ku and several other proteins, including topoisomerase 2A (TOP2A), was also enhanced when neddylation was blocked by MLN4924 (Figure 3B; Table S1). This could occur because such factors directly interact with Ku on the chromatin and are therefore released with Ku, or, alternatively, these factors might interact with chromatin in other ways, in a manner that is regulated by neddylation. Strikingly, almost all the proteins whose association with Ku was diminished upon MLN4924 treatment comprised factors associated with the 26S proteasome, as well as the segregase/unfoldase VCP (valosin containing protein; also known as p97) that targets ubiquitylated proteins to dissociate them from molecular assemblies, frequently promoting their proteasomal degradation (Meerang et al., 2011). Notably, MLN4924 treatment did not affect the levels of VCP or proteasome components (Figure S3I). Collectively, these findings suggested that VCP and proteasomal components recognize DNA-damage-dependent, NEDD8-mediated ubiquitylation of Ku, and/or other NHEJ components.

### Neddylation Promotes Ku Ubiquitylation following DNA-Damage Induction

**Figure 4:**
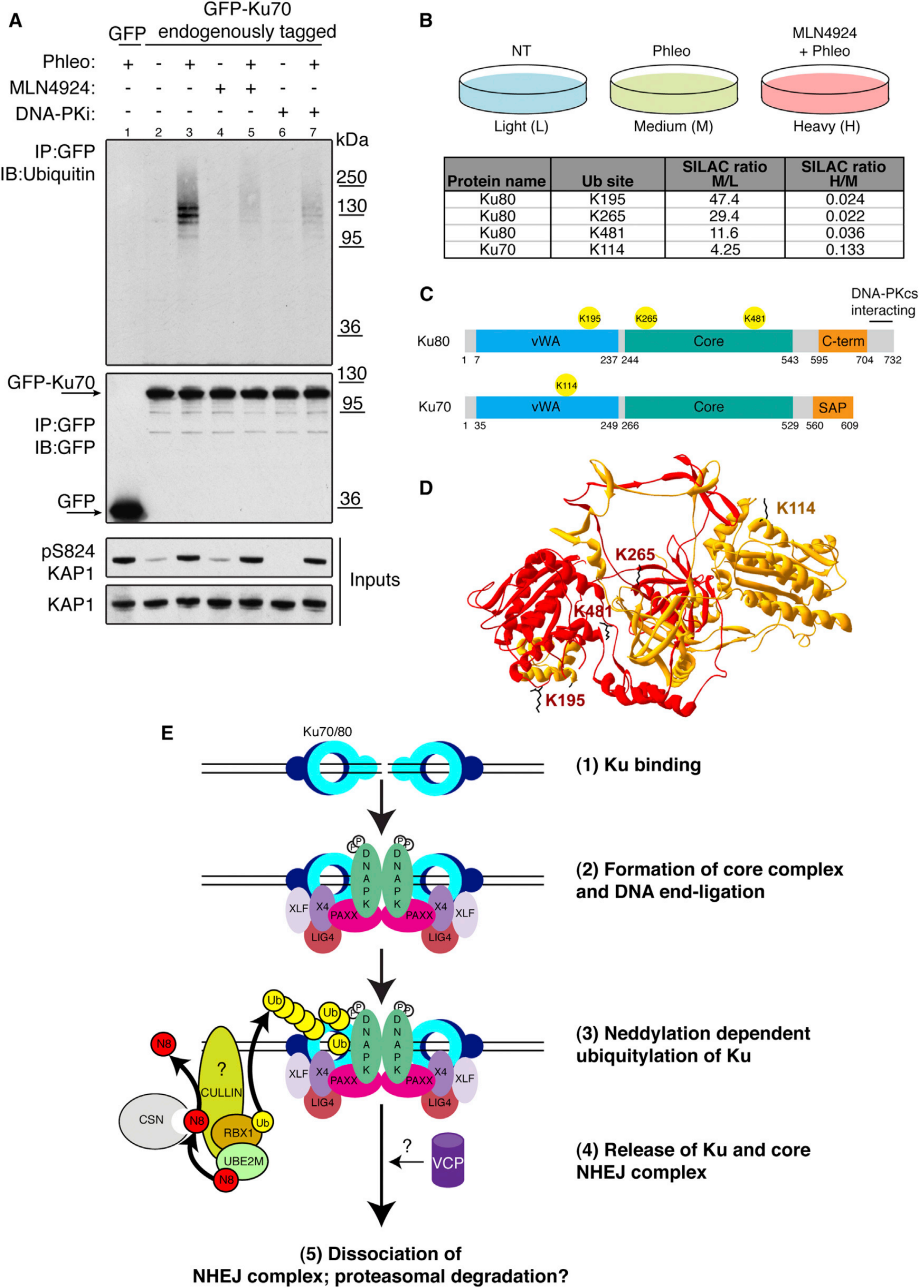
MLN4924 Blocks Ku Ubiquitylation after DNA Damage (A) In vivo ubiquitylation assay. RPE-1 cells expressing Ku70 endogenously tagged with GFP (lanes 2–7) or RPE-1 cells stably expressing GFP (lane 1) were pre-treated with DMSO, 3 μM MLN4924, or 3 μM DNA-PK inhibitor for 1 hr prior to treatment with 500 μM phleomycin (Phleo) for 1 hr as indicated. Cell lysates were immunoprecipitated (IP) with GFP-specific antibody and immunoblotted (IB) with indicated antibodies. GFP-Ku70 IP was done under stringent conditions (see Experimental Procedures). Black arrows indicate GFP-Ku70 and GFP. Phosphorylated Ser824 of KAP1 is used as a DNA-damage marker. (B) Identification of Ku ubiquitylation sites by quantitative LC-MS/MS. RPE-1 cells stably expressing Ku70 endogenously tagged with GFP were labeled with light, medium, or heavy SILAC isotopes and treated as indicated. Ku70 was enriched with GFP-Trap agarose under stringent washing conditions. Enriched proteins were resolved by SDS-PAGE and proteolysed in gel with trypsin. Peptides were extracted from gel and analyzed by LC-MS/MS. SILAC ratio M/L >2 represents induction upon DNA damage; SILAC ratio H/M < 0.5 represents inhibition by MLN4924 (see also Table S2). (C) Schematic representation of Ku70 and Ku80 domains with neddylation-dependent ubiquitylation sites identified in (B). vWA, von Willebrand factor A; C-term, C terminus; SAP, SAF-A/B, Acinus, and PIAS. (D) Positions of DNA-damage-induced neddylation-dependent ubiquitylation sites in the context of the structure of the Ku heterodimer (ID:1JEQ). Ku70 and Ku80 are in orange and red, respectively, and the ubiquitylated side chains are in black. (E) Model. (1, 2) Ku and the NHEJ complex are recruited to sites of DSBs. (3) Neddylation-dependent ubiquitylation of Ku following completion of DNA repair. (4) Ku and NHEJ factors are released from sites of DNA damage. VCP might target ubiquitylated Ku to proteasome for degradation. N8, NEDD8; Ub, ubiquitin; P, phosphorylation (see text for details). See also Figure S4.

In light of the above findings, we tested whether neddylation might promote ubiquitylation of Ku. Thus, we immunoprecipitated, under stringent conditions (1 M NaCl; see Experimental Procedures), endogenous Ku70 from RPE-GFP-Ku70 cells (Britton et al., 2013). Subsequent immunoblotting revealed that Ku ubiquitylation was markedly increased by phleomycin treatment (Figure 4A, lanes 2 and 3), whereas no ubiquitylated species were detected in cells expressing GFP alone (lane 1). Furthermore, this ubiquitylation did not occur with DNA-damaging agents that do not directly yield DSBs (Figure S4A). Crucially, inhibiting neddylation with MLN4924 strongly reduced Ku ubiquitylation induced by phleomycin treatment (Figure 4A, lanes 4 and 5; note in Figure S4B that overall ubiquitylation in cells was not affected by MLN4924). Blocking NHEJ-mediated DSB repair with a DNA-PK inhibitor, which has been shown to inhibit Ku release from DNA-damage sites (Britton et al., 2013), also impaired phleomycin induced Ku ubiquitylation (Figure 4A, lanes 6 and 7), as did siRNA depletion of DNA ligase 4 (data not shown), suggesting that Ku ubiquitylation occurs as a consequence of DSB repair. Although Ma et al. (2013) suggested that RNF168 recruitment to DNA-damage sites is neddylation dependent, we found that RNF168 depletion did not affect Ku ubiquitylation (Figure S4C), nor did it enhance Ku, XRCC4, or XLF persistence on chromatin after treating cells with phleomycin (Figure S4D; see Figure S4E for RNF168 depletion).

To establish whether the ubiquitylation we observed above was occurring specifically on Ku, we analyzed ubiquitylation in GFP-Ku70 immunoprecipitates by SILAC-based LC-MS/MS. This identified three sites (K195, K265, and K481) on Ku80 and one (K114) on Ku70, upon which ubiquitylation was increased following phleomycin treatment (SILAC ratio M/L) and was blocked by MLN4924 pretreatment (SILAC ratio H/M; Figure 4B; Table S2; the mass spectrum of K481 is shown in Figure S4F as an example). We investigated whether the sites identified on Ku80 were required for damage-dependent ubiquitylation of Ku and release of Ku from DNA-damage sites. This established that Ku ubiquitylation in the context of an exogenously expressed Ku80 mutant, with lysines 195, 265, and 481 (3K-R) mutated to arginine, still occurred following DNA-damage (data not shown). In addition, we found that the mutant form of Ku was still recruited and released from DNA damaged chromatin with kinetics similar to those of the wild-type protein (data not shown). These data suggested that there may be functional redundancy between the mapped sites and further, as yet unidentified ubiquitylation sites on Ku80 and or Ku70. To try to address this issue, we generated a U2OS cell line expressing an inducible mutant of Ku80 with all but one lysine mutated to arginine (we excluded K265, which has been shown to make direct contact with the DNA and is therefore likely to be important for DNA binding; Walker et al., 2001). Unfortunately, this all-but-one lysine mutant protein was not recruited to DNA-damage sites (data not shown) and could not therefore be used for further experiments. We did not attempt to generate cell lines expressing inducible mutants of Ku80 in combination with lysine-to-arginine mutants of Ku70, and it is possible that dimerization with endogenous Ku70 is sufficient to enable ubiquitylation and release of Ku complexes containing the Ku80 3K-R protein.

## Discussion

We have shown that neddylation is a dynamic modification at DNA-damage sites and that neddylation promotes cell survival after DSB induction. Furthermore, we have established that neddylation promotes the ubiquitylation of Ku upon DNA repair, and that this is associated with the release of Ku and other NHEJ factors from repair sites. Significantly, our work has identified DNA-damage induced, neddylation-dependent ubiquitylation of K195, K265, and K481 in Ku80 and K114 in Ku70. Interestingly, K265 and K481 lie within the core DNA binding domain of Ku80, with K265 directly making contact with DNA (Walker et al., 2001), while K114 and K195 lie within the von Willebrand factor (vWF) A domains (Grundy et al., 2013) of Ku70 and Ku80, respectively, that are thought to mediate protein-protein interactions (Figures 4C and 4D). The locations of these ubiquitylation sites suggest how Ku ubiquitylation on these and other sites could both trigger the dissociation of Ku from other NHEJ proteins as well as being associated with its release from DNA.

Collectively, the available data suggest the following model (Figure 4E): first, following DSB induction, Ku, PAXX, DNA-PK, XRCC4, LIG4, and XLF assemble at the DNA-damage site (Davis and Chen, 2013; Ochi et al., 2015; Xing et al., 2015); next, following DNA repair, Ku is ubiquitylated in a DNA-damage- and neddylation-dependent manner to promote the release of Ku and other NHEJ factors from the site of repair. Significantly, our proteomics analyses revealed that VCP and various proteasome subunits interact with Ku in a DNA-damage induced and neddylation-dependent manner. It is known that VCP can unfold ubiquitylated proteins and is important for extracting certain DNA-repair proteins from chromatin (Dantuma et al., 2014), and, while a role for VCP in removing Ku from DNA has been proposed (Postow, 2011), it has not yet been demonstrated in the literature. Because it has been reported that Ku removal from damage sites is not affected by proteasome inhibition (Postow et al., 2008), we suggest that in addition to disrupting interactions between Ku, DNA, and other NHEJ components, Ku ubiquitylation likely promotes targeting by VCP, leading to extraction of Ku from chromatin, perhaps then followed by proteasome-dependent Ku degradation (Figure 4E). As the Ku70/Ku80 heterodimer forms a highly stable ring structure encircling DNA ends (Walker et al., 2001), if Ku remains DNA bound once a DSB has been repaired, it would likely interfere with various processes, particularly transcription and replication (Frit et al., 2000; Ono et al., 1996). By serving as a barrier to complete genome replication and/or segregation, the persistence of Ku and other NHEJ factors on repaired DNA could thus account for the decreased cell survival we have observed when neddylation is abrogated after DSB induction as well as reduced NHEJ-dependent cell colony formation in plasmid integration assays. We recognize, however, that neddylation might also regulate several DSB repair pathways and that there are likely to be multiple mechanisms accounting for IR hypersensitivity upon MLN4924 treatment. Finally, we note that because NEDD8 pathway components are overexpressed or mutated in many human cancers, NEDD8-pathway inhibition is a promising anti-cancer strategy (Watson et al., 2011). Accordingly, our findings highlight opportunities for combining MLN4924 with DSB-inducing agents and for exploring cancer-genetic backgrounds where this combination might be particularly effective.

## Experimental Procedures

For more details on experimental procedures, please refer to the Supplemental Experimental Procedures.

### DNA Damage and Drug Treatments

Cells were preincubated with inhibitors for 1 hr prior to genotoxic treatments, and MLN4924 (Active Biochem) was used at 3 μM unless otherwise indicated. ATMi (KU55933) and DNA-PKi (NU7441; Tocris Bioscience) were used at 10 and 3 μM, respectively. PARPi (olaparib; Stratech Scientific) was used at 10 μM. ATRi (ATR-45; OSUCCC Medicinal Chemistry, Ohio State University) was used at 1 μM. X-ray irradiation was performed with a calibrated irradiation system (Cell Rad Faxitron) fitted with an aluminum filter for soft X-rays. Cells were irradiated in culture medium at room temperature, and standard, 10-Gy irradiation required an exposure time of 3 min 36 s. Phleomycin (Melford Labs) was used at 500 μM for 1 hr, Carboplatin (Sigma-Aldrich) was used at 100 μM for 1 hr, and Camptothecin (Sigma-Aldrich) was used at 1 μM for 1 hr. Cells were UV-irradiated with 10 J/m^2^ and analyzed 1 hr after.

### Detection of Ku

For detection of Ku on chromatin and Ku IRIF, cells were processed as described previously (Britton et al., 2013).

### Laser Microirradiation and Immunofluorescence

Laser microirradiation of cells and immunofluorescence were as previously described (Galanty et al., 2012).

### In Vivo Ubiquitylation of Ku

RPE-1 cells endogenously tagged with GFP-Ku70 at one chromosomal allele (Britton et al., 2013) grown in 10-cm plates were pretreated with DMSO or MLN4924 (3 μM, 1 hr) and then treated with Phleomycin (500 μM, 1 hr) and lysed in a lysis buffer (20 mM Tris [pH 7.5], 40 mM NaCl, 2 mM MgCl_2_, 10% glycerol, 0.5% NP-40) containing benzonase 18 U (Novagen) and supplemented with EDTA-free protease inhibitors (Roche) at room temperature. Lysates were adjusted to 0.5 M NaCl and incubated on ice for 30 min and cleared of debris by centrifugation at 4°C at 21130 relative centrifugal force (rcf). IP of GFP-tagged Ku70 was carried out with GFP-trap agarose beads (ChromoTek) for 2 hr at 4°C. Beads were washed four times in lysis buffer containing 1 M NaCl and subjected to SDS-PAGE and immunoblotting.

### Clonogenic Survival Assay

Cells were seeded at low density, in triplicate, at two dilutions, in 6-well plates and treated with IR after 24 hr. Cells were left to recover at 37°C for 10–14 days to allow colony formation. Cells were stained with 0.5% crystal violet/20% ethanol and counted. Results were normalized to plating efficiencies.

### Random Plasmid Integration Assay

Assays were performed as previously described (Galanty et al., 2012).

### Statistical Analysis

When required, an unpaired Student’s t test was calculated using GraphPad software (www.graphpad.com). Quantifications are based on at least three independent experiments unless otherwise specified. In all figures, significant differences between specified pairs of conditions, as judged by the t test, are highlighted by asterisks (^∗^p < 0.05; ^∗∗^p < 0.01; ^∗∗∗^p < 0.001; ^∗∗∗∗^p ≤ 0.0001).

## Author Contributions

J.S.B., N.L., and S.P.J. conceived and designed experiments. Y.G. and S.B. contributed to experimental design and set up. J.S.B., N.L., M.S.-C., S.B., and C.l.-S. performed experiments. P.B. performed mass spectrometry and analyzed the results. J.S.B., N.L., and S.P.J. wrote the manuscript. M.S.-C. and S.B. should be regarded as joint second authors. All authors contributed to the discussion of results and manuscript corrections.

## Supplemental Information

Document S1. Supplemental Experimental Procedures, Figures S1–S4, and Tables S3–S5

Table S1. Ku70 Interactors Identified by LC-MS/MS

Table S2. Ku Ubiquitylation Sites Identified by LC-MS/MS

Document S2. Article plus Supplemental Information
