# Supplementary material for: Neddylation Promotes Ubiquitylation and Release of Ku from DNA-Damage Sites
Source: Cell Rep. 2015 Apr 23;11(5):704–14. doi: 10.1016/j.celrep.2015.03.058 (PMC4431666; doi:10.1016/j.celrep.2015.03.058)
Supplement: Document S1. Supplemental Experimental Procedures, Figures S1–S4, and Tables S3–S5 [file mmc1.pdf]

Cell Reports

Supplemental Information

Neddylation Promotes Ubiquitylation and Release of Ku from DNA-Damage Sites

Jessica S. Brown, Natalia Lukashchuk, Matylda Sczaniecka-Clift, Sébastien Britton, Carlos le Sage, Patrick Calsou, Petra Beli, Yaron Galanty, and Stephen P. Jackson

Supplementary Information

Supplemental Figures

Figure S1 (related to Figure 1)

Figure S2 (related to Figure 2)

Figure S3 (related to Figure 3)

Figure S4 (related to Figure 4)

Supplemental Tables

Table S1. Ku70 interactors identified by LC-MS/MS (separate file)

Table S2. Ku ubiquitylation sites identified by LC-MS/MS (separate file)

Table S3. Antibodies used in this study

Table S4. siRNA sequences used in this study

Table S5. Primers used in this study

Supplemental Experimental Procedures

Supplemental References

Supplementary figures

Figure S1 relates to Figure 1.

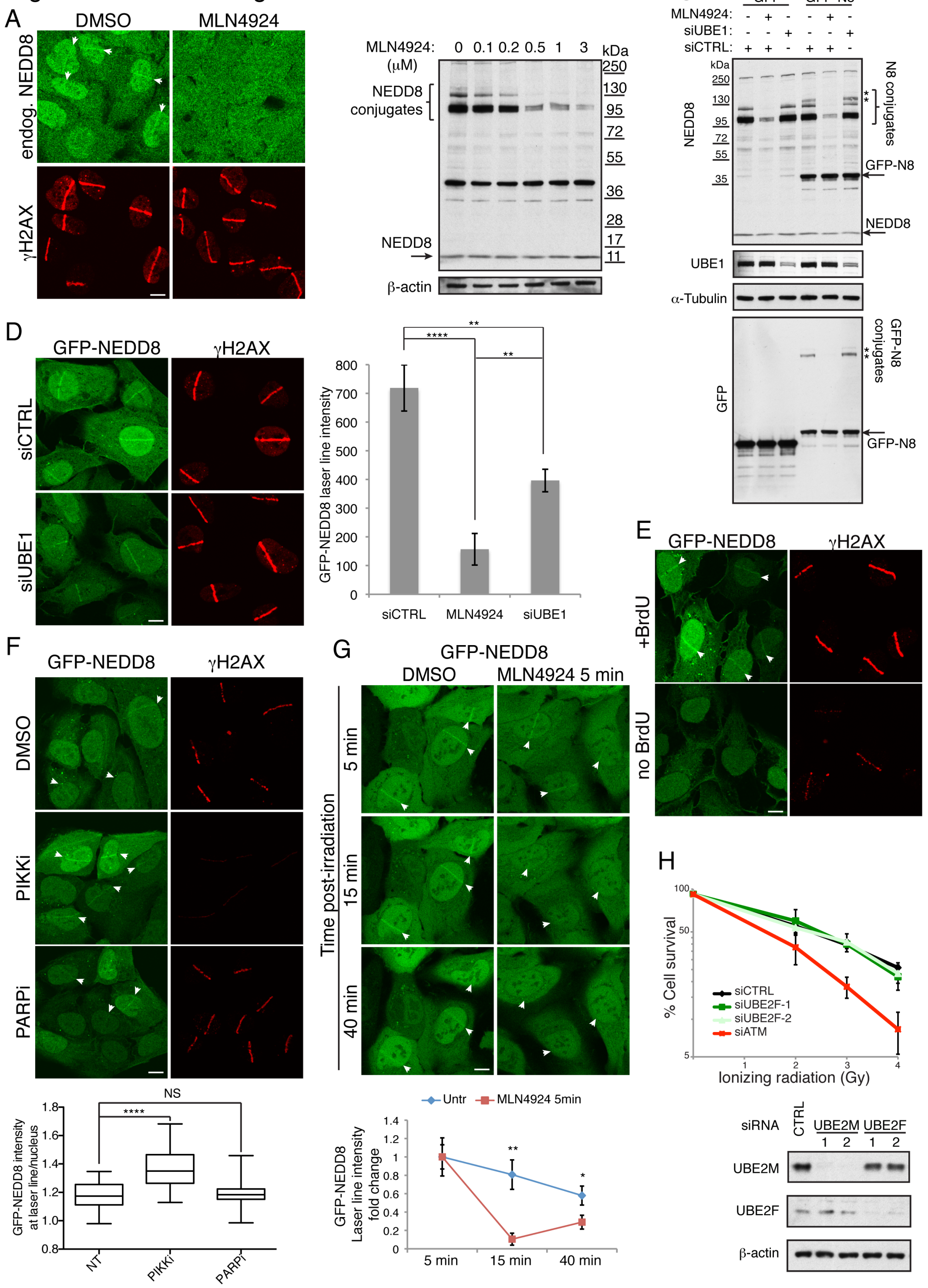

Figure S1 (related to Figure 1).

(A) MLN4924 blocks endogenous NEDD8 recruitment to DNA damage sites. U2OS cells were pre-treated for 1 h with DMSO or 3 μM MLN4924 and laser microirradiated. Cells were fixed after 20 min and visualized by immunofluorescence as indicated. White arrows mark sites of endogenous NEDD8 recruitment. White bar = 10 μM.

(B) MLN4924 inhibits neddylation. U2OS cells were treated with increasing doses of MLN4924 (as indicated) for 1 h and whole cell extracts were analyzed by immunoblotting with NEDD8- and β-actin-specific antibodies. Molecular marker is indicated.

(C) GFP-NEDD8 conjugation in a U2OS-GFP-NEDD8 stable cell line is dependent on NEDD8 E1 activity. U2OS-GFP-NEDD8 or U2OS-GFP cells were transfected with the indicated siRNAs and pre-treated for 1 h with 3 μM MLN4924 where indicated. Whole cell extracts were analyzed by immunoblotting with the indicated antibodies. The NEDD8 specific antibody recognizes free NEDD8 (~11 kDa) and neddylated substrates (mostly cullins) between 95 and 130 kDa (N8 conjugates). Importantly, GFP-NEDD8 conjugation was abrogated by pre-treatment with MLN4924, but not by depleting the ubiquitin E1 UBE1. Black arrows mark free, GFP- and endogenous NEDD8. Asterisks indicate GFP-neddylated cullins. N8 – NEDD8.

(D) UBE1 depletion reduces GFP-NEDD8 recruitment to DNA damage sites. GFP-NEDD8 cells were transfected with siRNA against UBE1 or control (CTRL) and laser microirradiated. Cells were fixed after 20 min and visualized by immunofluorescence as indicated. Quantification shows the average intensity of GFP-NEDD8 signal at the laser line (see supplementary experimental procedures

for details). Data are from the same experiment as Figure 1B. Error bars represent standard deviation between three independent experiments (asterisks as Figure 1B). White bar = 10 μM.

(E) GFP-NEDD8 is recruited to DSBs. U2OS cells stably expressing GFP-NEDD8 were untreated or incubated with 10 μM BrdU for 24 h prior to microirradiation. Cells were fixed after 20 min and visualized by immunofluorescence. White bar = 10 μM.

(F) GFP-NEDD8 recruitment is not dependent on the DDR PI-3-Kinases or PARP activity. U2OS-GFP-NEDD8 cells were pre-treated for 1 h with 10 μM ATM inhibitor, 1 μM ATR inhibitor and 3 μM DNA-PK inhibitor (labeled PIKKi), with 10 μM PARP inhibitor (PARPi) or with DMSO and microirradiated at 200 μW laser power. Cells were fixed after 20 min and visualized by immunofluorescence. White arrows mark sites of GFP-NEDD8 recruitment. Quantification shows fold change in GFP-NEDD8 intensity at the laser line compared to background of a representative experiment. Graph shows median intensity values with inter-quartile range. P-values were calculated using the non-parametric Mann-Whitney test (asterisks as Figure 1B). White bar = 10 μM.

(G) GFP-NEDD8 recruitment to sites of damage is a dynamic process. Live cell imaging of U2OS cells stably expressing GFP-NEDD8 pre-treated for 5 min with DMSO or 3 μM MLN4924 and laser microirradiated. White arrows mark sites of GFP-NEDD8 recruitment. Graph shows fold change of GFP-NEDD8 intensity at laser lines from 5 min time point. Error bars represent standard deviation between three independent experiments (asterisks as Figure 1B). White bar = 10 μM.

(H) Depletion of UBE2F does not cause cellular hypersensitivity to IR. Clonogenic cell survivals were performed in U2OS cells transfected with the indicated siRNA and subjected to increasing doses of IR. Immunoblot shows depletion of UBE2M and UBE2F. As UBE2F and UBE2M co-depletion was highly toxic, we were unable to test the effects of their combined depletion on cell survival after IR. Each point represents an average of at least three independent experiments. Error bars correspond to standard deviations between experiments (asterisks as Figure 1B).

Figure S2 relates to Figure 2.

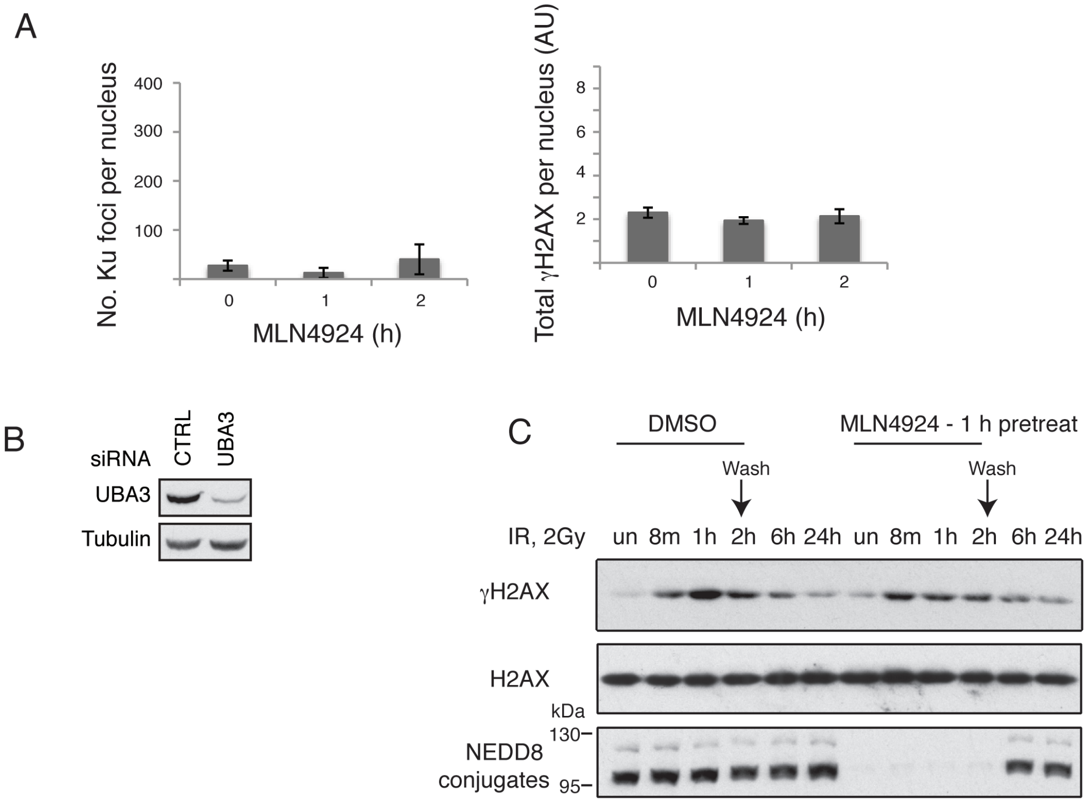

Figure S2 (related to Figure 2).

(A) MLN4924 does not affect the number of Ku80 foci (left panel) or total γH2AX intensity (right panel) per nucleus in undamaged cells. U2OS cells were treated with 3 μM MLN4924 for the indicated time points and analyzed by immunofluorescence. The graphs show the average of at least two independent experiments -/+ standard deviation (AU, arbitrary units).

(B) Immunoblot showing siRNA depletion of endogenous UBA3 in U2OS cells.

(C) Short treatment with MLN4924 does not affect γH2AX recovery following IR. U2OS cells were treated with 3 μM MLN4924 or DMSO for 1 h prior to treatment with 2 Gy IR. Cells were lysed at indicated time points after IR. MLN4924 was washed off 2 h following DNA damage (to prevent induction of DNA damage by MLN4924). Cell lysates were subject to immunoblotting with the indicated

antibodies. Note that neddylation is inhibited by MLN4924 but recovers 4 h after wash-off.

Figure S3 relates to Figure 3.

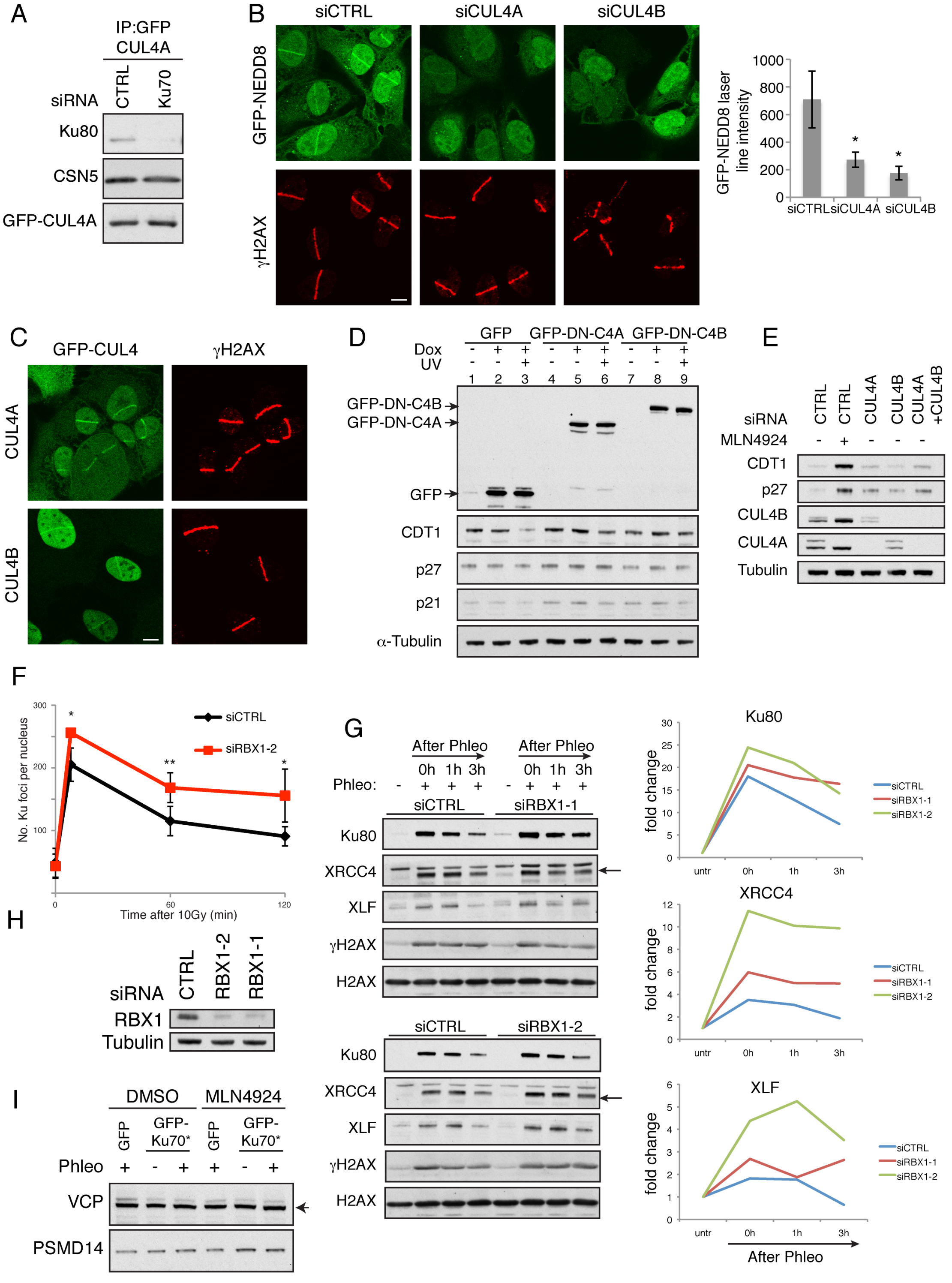

* Endogenously tagged

Figure S3 (related to Figure 3)

(A) GFP-CUL4A interacts with Ku80. U2OS cells stably expressing GFP-CUL4A were transfected with siRNA against Ku70 or control (CTRL). Cells were treated for 1 h with 500 μM Phleomycin. Cell lysates were immunoprecipitated with GFP-Trap beads and immunoblotted with the indicated antibodies. Depletion of Ku70 causes instability and decreases the levels of Ku80 (Britton et al., 2013),

(B) Depletion of CUL4A or CUL4B impairs NEDD8 recruitment to DSB sites. U2OS-GFP-NEDD8 cells were transfected with siCTRL, siCUL4A or siCUL4B (pool of 2 siRNAs) and subjected to laser microirradiation. Cells were fixed after 20 min and stained with γH2AX antibody. Quantification shows the average intensity of GFP-NEDD8 signal at the laser line (see Supplementary Experimental procedures for details) from three independent experiments. Error bars represent standard deviation between experiments (asterisks as Figure 1B). White bar = 10 μM.

(C) GFP-CUL4A and GFP-CUL4B are recruited to DNA damage sites. U2OS cells stably expressing GFP-CUL4B or inducible GFP-CUL4A were subjected to laser microirradiation. Cells were fixed after 20 min and stained with γH2AX antibody. White bar = 10 μM.

(D) Cell lines stably expressing DN-CUL4A or DN-CUL4B do not inhibit CRL4 activity efficiently. U2OS cell lines stably expressing inducible GFP, DN-GFP-CUL4A (C4A) or DN-GFP-CUL4B (C4B) were generated. GFP, DN-GFP-CUL4A or DN-GFP-CUL4B expression was induced with Doxycycline (Dox) as indicated 24 h prior to cell lysis (lanes 2+3, 5+6, 8+9). Cells were treated with 10 J/m^2 UV where indicated and collected 1 h later. CDT1 is degraded after UV in cells expressing GFP only (lanes 2 and 3). Expression of DN-CUL4A or DN-CUL4B

stabilizes CDT1 levels after UV damage as previously described (lanes 5 and 6; 8 and 9; (Emanuele et al., 2011). Basal levels of CDT1 and p21 were unaffected upon expression of DN-CUL4A and DN-CUL4B (lanes, 4 and 5, 7 and 8) and basal levels of p27 were marginally stabilized following DN-CUL4B (lanes 7 and 8), but not DN-CUL4A (lanes 4 and 5) expression.

(E) siRNA depletion of CUL4A or CUL4B fails to efficiently inhibit CRL4 activity. CUL4A and CUL4B were depleted using the indicated siRNAs (siCUL4A-2 or siCUL4B-1) either alone or in combination. Where indicated, 1 μM MLN4924 was added 16 h prior to cell lysis. Cell lysates were subject to immunoblotting with the indicated antibodies. MLN4924 treatment caused marked stabilization of CDT1 and p27 representing effective CRL4 inhibition (Emanuele et al., 2011; Higa et al., 2006). siRNA depletion of CUL4A had marginal effects on CDT1 and p27 stabilization compared to MLN4924.

(F) RBX1 depletion increases Ku foci numbers following DNA damage. U2OS cells were transfected with siRNA against RBX1 or control (siCTRL) and then treated with 10 Gy IR. Cells were collected and analyzed as Figure 2A+B. The higher number of Ku foci detected at 8 min in the siRBX1 sample likely reflects a failure to release Ku at this early time-point compared to control cells.

(G) RBX1 depletion increases retention of Ku and NHEJ factors on the chromatin following DNA damage. U2OS cells were transfected with two independent siRNAs against RBX1 or control (siCTRL) and then treated with 500 μM Phleomycin for 1 h. Samples were collected as in Figure 3A. Intensity of bands was quantified using Fiji software and intensities for Ku80, XRCC4 and XLF were normalized to intensity of H2AX. Graphs show fold change from the untreated sample.

(H) Immunoblot showing siRNA depletion of RBX1 in U2OS cells.

(I) MLN4924 treatment does not affect levels of VCP or PSMD14. RPE1 cells expressing GFP or endogenously tagged with GFP-Ku70 were treated with 3 μM MLN4924 or DMSO for 1 h, followed by 500 μM Phleomycin for 1 h as indicated. Whole cell extracts were immunoblotted with the indicated antibodies. Black arrow indicates VCP.

Figure S4 relates to Figure 4.

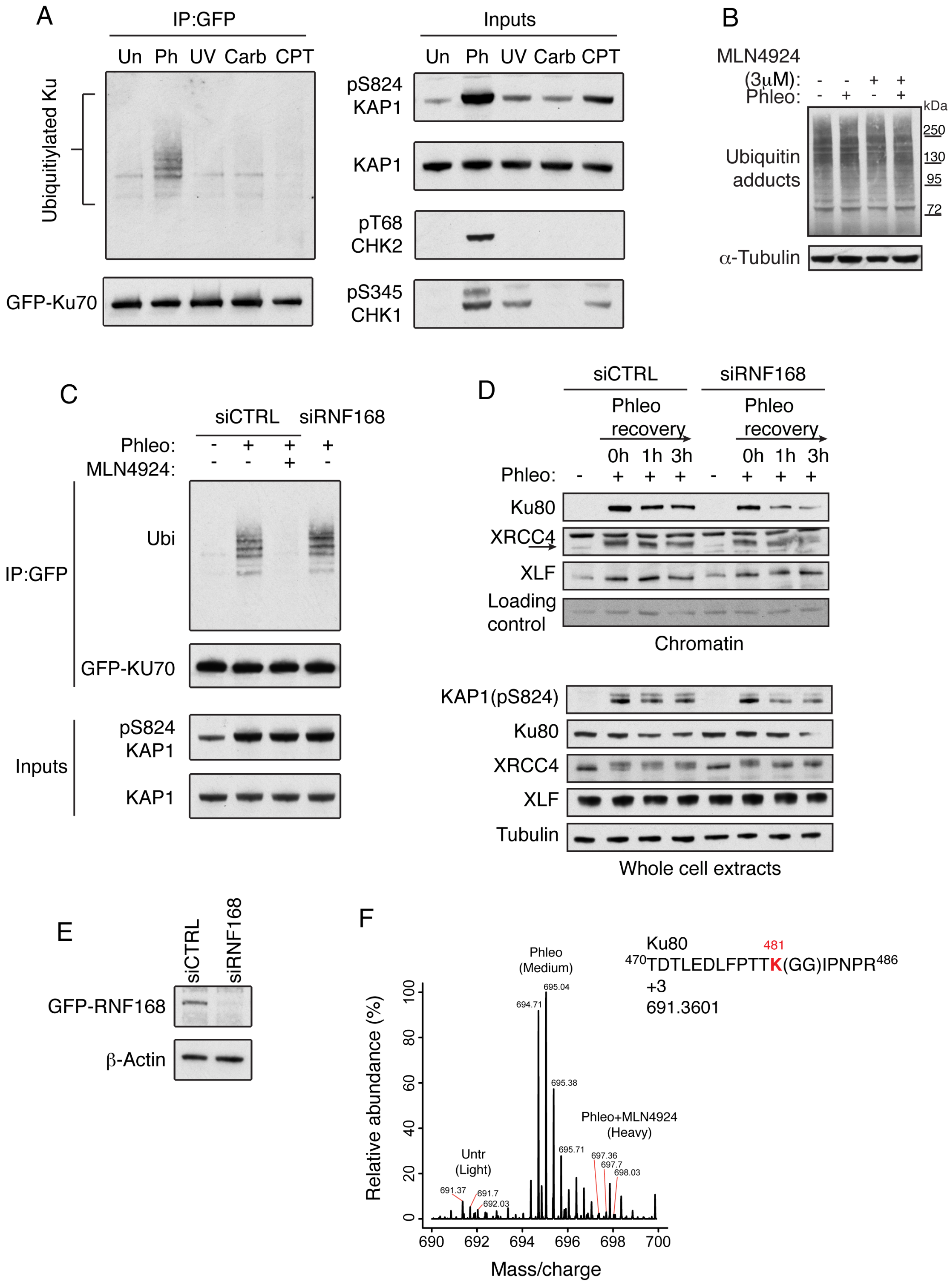

Figure S4 (related to Figure 4)

(A) Ku ubiquitylation occurs after Phleomycin, which induces DSBs directly. *In vivo* ubiquitylation assay was performed as in Figure 4A in RPE-GFP-Ku70 cells untreated (Un) or treated with 500 μM Phleomycin (Ph), 10J/m^2 UV, 100 μM Carboplatin (Carb), 1 μM Camptothecin (CPT), and collected 1h after.

(B) MLN4924 does not affect overall cellular ubiquitylation detected by immunoblotting. U2OS cells were pre-treated with DMSO or 3 μM MLN4924 for 1 h, followed by treatment with 500 μM Phleomycin for 1 h. Cell lysates were subject to immunoblotting with the Ubiquitin- or α-Tubulin-specific antibodies. Molecular marker is indicated.

(C) RNF168 depletion does not affect Ku ubiquitylation following DNA damage. *In vivo* ubiquitylation assay was performed as in Figure 4A in RPE-GFP-Ku70 cells transfected with siRNA against RNF168 or control (siCTRL) and treated with 500 μM Phleomycin (Phleo) for 1h. As a positive control, siCTRL cells were also pretreated with 3 μM MLN4924 for 1h prior to Phleomycin treatment. Ubi – ubiquitination recognized by anti-Ubiquitin antibody.

(D) RNF168 depletion does not cause persistence of Ku on the chromatin following DNA damage. U2OS cells were transfected with siRNA against RNF168 or control (siCTRL). Cells were treated with Phleomycin as indicated and the assay was performed as in Figure 3A. A non-specific antibody band is used as a loading control.

(E) Immunoblot showing siRNA depletion of GFP-RNF168 in U2OS cells stably expressing GFP-RNF168 transfected with siCTRL or siRNF168.

(F) DNA damage-induced ubiquitylation of Ku80 on Lys 481 is inhibited by MLN4924 treatment. The mass spectrum shows the relative abundance of the Ku80 peptide TDTLEDLFPTTK(GG)IPNPR (aa470-486) in untreated (SILAC light) and phleomycin-treated cells without (SILAC medium) and with MLN4924 pre-treatment (SILAC heavy). The peptide sequence, mass/charge (m/z) and charge state are indicated.

Supplementary tables

Table S3. Antibodies used in this study.

Target	Mono/ polyclonal	Reference	Raised in	Source	Dilution for I.B	Dilution for I.F
α–Tubulin	Monoclonal	T9026	Mouse	Sigma	1:20 000	
β-Actin	Monoclonal	ab8226	Mouse	Abcam	1:5000	
CHK1pS345	Polyclonal	2348	Rabbit	Cell signaling	1:5000	
CHK2pT68	Polyclonal	2661S	Rabbit	Cell signaling	1:1000	
CDT1	Polyclonal	ab70829	Rabbit	Abcam	1:1000	
CSN5	Polyclonal	A300-014A	Rabbit	Bethyl	1:5000	
CUL1	Monoclonal	ab7581	Rabbit	Abcam	1:1000	
CUL4A	Monoclonal	ab92554	Rabbit	Abcam	1:20000	
CUL4B	Monoclonal	ab76470	Rabbit	Abcam	1:1000	
FLAG	Monoclonal	F3165	Mouse	Sigma	1:1000	
GFP	Monoclonal	11814460001	Mouse	Roche	1:1000	
LIG4	Polyclonal	(Riballo et al., 1999)	Rabbit		1:1000	
γH2AX	Monoclonal	05-636	Mouse	Millipore	1:1000	1:250
γH2AX	Polyclonal	2577	Rabbit	Cell signaling		1:250
H2AX	Polyclonal	ab11175	Rabbit	Abcam	1:5000	
Histone H1	Polyclonal	ab1938	Sheep	Abcam	1:1000	
KAP1 (pS824)	Polyclonal	IHC-00073	Rabbit	Bethyl	1:1000	
KAP1	Polyclonal	ab10483	Rabbit	Abcam	1:2000	
Ku70	Monoclonal	Ab3114	Mouse	Abcam	1:200	
KU80	Monoclonal	LVMS285P1	Mouse	Fisher Scientific	1:2000	1:100
NEDD8	Monoclonal	1571-1	Rabbit	Epitomics	1:5000	1:250
P21	Polyclonal	Sc397	Rabbit	Santa Cruz	1:1000	
P27	Monoclonal	610241	Mouse	BD Bioscience	1:2000	
PSMD14	Monoclonal	ab109123	Rabbit	Abcam	1:1000	
UBA3	Monoclonal	ab124728	Rabbit	Abcam	1:10 000	
UBE1	Monoclonal	ab34711	Rabbit	Abcam	1:1000	
UBE2F	Polyclonal	ab15707	Goat	Abcam	1:1000	
UBE2M	Monoclonal	ab109507	Rabbit	Abcam	1:10 000	
Total Ubiquitin	Polyclonal	3933	Rabbit	Cell signaling	1:1000	
Ubiquitin (FK2)	Monoclonal	PW8810	Mouse	Enzo Life Sciences	1:1000	
VCP	Monoclonal	612183	Mouse	BD Bioscience	1:1000	
XLF	Polyclonal	ab33499	Rabbit	Abcam	1:500	
XRCC4	Polyclonal	ab145	Rabbit	Abcam	1:2000	

Table S4. siRNA sequences used in this study

Target	Target sequence	CDS/UTR
ATM	GAC UUU GGC UGU CAA CUU UCG	CDS
CTRL (Luciferase)	CGU ACG CGG AAU ACU UCG A	CDS
CUL4A-1	GAA GAU UAA CAC GUG CUG G	CDS
CUL4A-2	GCU UAG AGG AAG AGG GAG A	CDS
CUL4B-1	CAG AAU UUA AAG AGG GUA A	CDS
CUL4B-2	GGA ACA UCA UAG AAG AGA A	3’ UTR
Ku70	GAG UGA AGA UGA GUU GAC A	CDS
RBX1-1	GGG AUA UUG UGG UUG AUA A	CDS
RBX1-2	CCA UUG GAC AAC AGA GAG U	CDS
RNF168	GGC GAA GAG CGA UGG AGG A	CDS
UBA3	AGA GAG AGA UUA UGA GCA A	3’ UTR
UBE1	CGU CAG ACC UGC AAG AGA A	CDS
UBE2F-1	GGA AUA AAG UGG AUG ACU A	CDS
UBE2F-2	CAA CAU AAA UAC AGC AAG A	3’ UTR
UBE2M-1	AGC CAG UCC UUA CGA UAA A	CDS
UBE2M-2	GAU GAG GGC UUC UAC AAG A	CDS
XRCC4	AUA UGU UGG UGA ACU GAG A	CDS

Table S5. Primers used in this study

ID	Sequence	Restriction site
CUL4A-F	CT AAA GCT TCT GCG GAC GAG GCC CCG CGG AAG	HindIII
CUL4A-R	CTA GGA TCC TCA GGC CAC GTA GTG GTA CTG	BamHI
DN-CUL4A-F	CTC AAG CTT CCG CGG ACG AGG CCC C	HindIII
DN-CUL4A-R	TAG AAT TCC TAC TTG TCA TCG TCA TCC TTG TAG	EcoRI
DN-CUL4B-F	CTC AAG CTT CCA TGT CAC AGT CAT CTG GAT CA	HindIII
DN-CUL4B-R	GTA GGA TCC CTA CTT GTC ATC GTC ATC CTT GTA G	BamHI
NEDD8-F	AAG GCT CGA GCT CTA ATT AAA GTG AAG ACG CTG ACC	XhoI
NEDD8-R	GGA TCC CTA TCC TCC TCT CAG AGC CAA CAC	BamHI
UBE2F-F	TACT CGA GTG CTA ACG CTA GCA AGT AAA C	XhoI
UBE2F-R	TA GGA TCC TCT GGC ATA ACG TTT GAT G	BamHI
UBE2M-F	TAGT CGA CTG ATC AAG CTG TTC TCG CTG	SalI
UBE2M-R	TA AGA TCT TTT CAG GCA GCG CTC AAA GTA	BglII

Supplementary Experimental Procedures.

Cell Culture

Cells were grown in a 5% CO_2 humidified incubator at 37°C. U2OS cells were grown in DMEM supplemented with 10% FBS, 2 mM L-glutamine, 100 U/ml penicillin, and 100 μg/ml streptomycin. RPE-1 - human telomerase reverse transcriptase (hTERT) expressing cells were grown in DMEM/Ham's F12 medium supplemented as previously and buffered with sodium bicarbonate. U2OS cells stably expressing GFP, GFP-NEDD8, GFP-UBE2M, GFP-UBE2F, GFP-CSN5, GFP-CUL4B, GFP-RNF168, FLAG-UBA3 WT and FLAG-UBA3 A171T and RPE-1 cells stably expressing GFP were grown in medium supplemented with 0.5 mg/ml G418 (GIBCO, Life Technologies). U2OS cells stably expressing doxycycline-inducible GFP-CUL4A were grown in medium supplemented with 0.5 mg/ml G418 (GIBCO, Life Technologies) and 2 μg/ml Blasticidin (Invitrogen), and 10% Tet-negative FBS. U2OS cells stably expressing doxycycline-inducible DN-GFP-CUL4A or DN-GFP-CUL4B were grown in medium supplemented with 0.25 mg/ml G418 and 10% Tet-negative FBS.

siRNA transfections

siRNA transfections were done using Lipofectamine RNAiMAX (Life Technologies) according to manufacturers instructions. Two-rounds of transfection were performed 24 h apart for optimal depletion of the target protein. Experiments were performed 72 h after the 1st transfection, 144 h for siRNA Ku70. siRNA duplexes were purchased from MWG Biotech and the

sequences used are listed in Table S4. A sequence against firefly luciferase was used as siCTRL.

Plasmids.

For a list of all primers used in this study, see Table S5. All constructs were fully sequenced and are mutation free. NEDD8 was PCR-amplified from a human fetal brain cDNA library and cloned into pEGFP-C1. The original mammalian constructs of UBE2M and UBE2F were provided by the Division of Signal Transduction Therapy, Department of Biochemistry, Medical Sciences Institute, University of Dundee. They were PCR amplified and cloned into pEGFP-C1 vectors. The original constructs expressing GFP-tagged human CUL4A and CUL4B were provided by Prof. Nico Dantuma (Karolinska Institute, Sweden) and Prof. Changshun Shao (Rutgers University, US), respectively. CUL4A was PCR amplified with CUL4A-F and CUL4A-R primers and cloned into pEGFP-TO-C1 (TO – Tet-operator). GFP-DN-CUL4A-FLAG and GFP-DN-CUL4B-FLAG plasmids for inducible expression were constructed by subcloning pcDNA3-DN-hCUL4A-FLAG or pcDNA3-DN-hCUL4B-FLAG (Addgene) into the pEGFP-TO-C1 vector by PCR, using DN-CUL4A-F, DN-CUL4A-R, DN-CUL4B-F or DN-CUL4B-R primers. Sylvie Urbe and Michael Clague (Cellular and Molecular Physiology, Institute of Translational Medicine, University of Liverpool, Liverpool, UK) provided the plasmid expressing human GFP-CSN5. The FLAG-UBA3 WT and A171T mutant constructs have been described previously (Toth et al., 2012) and were provided by Matthew Petroski (Sanford-Burnham Medical Research Institute, CA, USA). HA-Ubiquitin has been described previously (Galanty et al., 2012).

Plasmid transfections and stable cell lines

Plasmid transfections were done using TransIT-LT1 (Mirus) according to manufacturers protocol. To generate monoclonal stable cell lines expressing inducible GFP-CUL4A, 48 h after transfection of U2OS TRex (Tet-repressor) cells with pEGFP-TO-CUL4A plasmid, cells were seeded at low density, in the presence of 0.5 mg/ml G418 and 2 μg/ml Blasticidin (Invitrogen). Individual clones were isolated and grown in duplicate in the presence of selection media. Clones were treated with 2 μg/ml doxycycline overnight to induce GFP-CUL4A expression and were selected based on expression of full-length protein, as validated by immunoblotting. To generate polyclonal stable cell lines expressing inducible GFP, GFP-DN-CUL4A or GFP-DN-CUL4B, U2OS TRex cells were transfected with pEGFP-TO-C1, or GFP-DN-CUL4A or GFP-DN-CUL4B using FuGENE HD (Promega) and cultured with 0.5 mg/ml G418 added to the medium for two weeks. Expression was induced by 2 μg/ml doxycycline treatment for 24 h and verified by immunoblotting and microscopy.

To generate all other stable cell lines; 48 h after transfection, cells were seeded at low density, in the presence of 0.5 mg/ml G418 (GIBCO, Life Technologies). Individual clones were isolated, grown in the presence of selection media and again, expression of full-length protein was validated by immunoblotting.

U2OS-GFP-RNF168 cells were generated as previously described (Giunta et al., 2010).

Immunoblotting

For whole cell extracts cells were lysed in an SDS lysis buffer (4% SDS, 20% Glycerol, 125 mM Tris-HCl pH 6.8) and protein concentration was measured. Lysates were then diluted to equal concentration and supplemented with 10% β-Mercaptoethanol and 0.005% Bromophenol blue. Samples were resolved on 4-12% gradient Bis-Tris NuPAGE gels (Novex, Life Technologies) and transferred onto nitrocellulose membranes (GE Life Sciences). Membranes were blocked in 5% milk in TBS containing 0.1% Tween-20 and incubated with the corresponding primary antibody (Table S3) followed by an appropriate secondary antibody coupled to horseradish peroxidase (Fisher Scientific). Detection was performed with ECL reagent (GE Healthcare). Quantification of the immunoblots was done using Fiji software (http://fiji.sc/Fiji).

Immunofluorescence

For laser microirradiation experiments, cells were washed three times with PBS and then fixed in 2% PFA (15 min). Cells were permeabilised with 0.2% Triton X-100/PBS (5 min) and then blocked with 5% BSA in PBS/0.1% Tween 20 (PBS-T) for 10 min. Cells were stained for 1 h at room temperature with the indicated primary antibodies in 5% BSA/PBS-T, washed with PBS-T and then stained for 1 h at room temperature with the appropriate goat secondary antibodies coupled to Alexa Fluor 488 or 568 fluorophores in 5% BSA/PBS-T.

Detection of Ku on chromatin

For the detection of Ku on chromatin (Figure 3A) cells were processed similarly to as described previously (Britton et al., 2013). Briefly, cells were pre-extracted twice for 3 min with CSK buffer (10 mM Pipes, pH 7.0, 100 mM NaCl, 300 mM sucrose, 3 mM $MgCl_2$ and 0.7% Triton X-100) supplemented with 0.3mg/ml RNase A for 3 min, then washed 3 times with PBS and collected in a SDS lysis buffer (all at room temperature) and processed for immunoblotting as described above.

Detection of Ku foci

Coverslips were prepared as described previously (Britton et al., 2013). Briefly, cells were seeded onto 160 µm coverslips (VWR International) 24 h prior to the experiment. Cells were washed three times with PBS and then pre-extracted by incubating twice for 3 min in CSK buffer (see Immunoblotting) supplemented with 0.3 mg/ml RNase A and washed three times in between with PBS (all at room temperature). Cells were then fixed in 2% PFA for 15 min. Before staining, cells were treated with PBS/0.2% Triton X-100 for 5 min, washed with PBS-T and then blocked with 5% BSA/PBS-T for 10 min. Cells were incubated in primary antibodies in 5% BSA/PBS-T for 1 h at room temperature, washed in PBS-T and then incubated for 1 h at room temperature with goat secondary antibodies coupled to Alexa Fluor 488 or 594 fluorophores (Life Technologies) in 5% BSA/PBS-T. Cells were mounted onto glass slides using Vectashield (Vector Laboratories).

High resolution microscopy and deconvolution

As described previously for the visualization of Ku foci (Britton et al., 2013), high-resolution images were acquired on a Deltavision PersonalDV (Applied Precision/GE Healthcare) equipped with a 1,024×1,024 CCD camera (CoolSNAP HQ2; Photometrics), and a 100× U Plan S Apochromat/1.40 NA oil objective (Olympus) and controlled with SoftWoRx software x5.5 (Applied Precision/GE Healthcare). Z stacks were taken at 0.2-μm intervals and the fluorescent channels were acquired sequentially. Deconvolution was then performed within SoftWoRx in conservative mode. Brightness and contrast were adjusted and images were cropped using Photoshop CS5 (Adobe).

Quantification of Ku foci

Deconvoluted images of >10 cells per sample were submitted to automatic focus detection using Volocity 6.3 (PerkinElmer). Cells were selected randomly based on DAPI staining. As the DNA content and therefore the number of double strand break induced foci doubles during the S- and G2-phases of the cell cycle, DAPI volume was used as a surrogate marker of DNA content. The number of foci detected per nucleus were therefore adjusted to the mean DAPI volume. Of note, a 1 h, 3 μM treatment of MLN4924 did not significantly alter the mean DAPI volume.

Laser microirradiation

Cells grown on glass-bottom dishes (Willco Wells) in phenol red-free complete medium (Invitrogen) were treated with 10 μM bromodeoxyuridine (BrdU) for 24 h. Laser microirradiation was performed using a FluoView 1000 confocal

inverted microscope (Olympus) equipped with a 37°C heating stage (Ibidi) and a 405 nm laser diode (6 mW) focused through a 60× UPlanSApo/1.35 oil objective to yield a spot size of 0.5-1 mm. The laser settings 0.40 mW output, 50 frames, unless otherwise indicated, were chosen to generate a detectable damage response in a manner dependent on BrdU presensitization and without noticeable cytotoxicity. Cells were analyzed by immunofluorescence using a confocal microscope (FluoView 1000; Olympus).

Quantification of Laser microirradiation experiments

Intensity of GFP-NEDD8 signal in the laser line was calculated by subtracting average nuclear intensity from average laser line intensity (Figures 1B, S1D, S3B and S1G) or by dividing average laser line intensity by average nuclear intensity (Fold change; Figure S1F). Intensity measurements were done using Fiji software (http://fiji.sc/Fiji). P-value was calculated using unpaired T-test (*, $P < 0.05$; **, $P < 0.01$; ***, $P < 0.001$; ****, $P \leq 0.0001$).

Random Plasmid Integration assay

Between 2-rounds of siRNA transfection, U2OS cells were transfected with BamHI–XhoI-linearized pEGFP-C1 (Clontech). 24 h after DNA transfection, cells were collected, counted and plated at low confluency onto: a 15 cm dish and 6 cm dish in complete medium, and a 15 cm dish in complete medium with 0.5 mg/ml G418 (GIBCO, Life technologies). The following day, transfection efficiency was calculated by determining the proportion of GFP-positive cells on the 6 cm dish. Cells on the 15 cm dishes were incubated at 37 °C for 10-14 days until colonies had formed. Colonies were stained with 0.5% crystal violet/20%

ethanol and counted. Random plasmid integration events were normalized to transfection and plating efficiencies. The P-value was calculated using an un-paired Student's t-test.

Identification of Ku ubiquitylation sites

RPE-1 cells expressing Ku70 endogenously tagged with GFP were grown in SILAC DMEM/Ham's F12 minus L-Lysine and L-Arginine (Thermo Fisher) supplemented with 10% dialysed FBS, 2 mM L-glutamine, 100 U/ml penicillin, and 100 μg/ml streptomycin, and containing either Lysine (K0)/Arginine (R0) (Light), Lysine (K4)/Arginine (R6) (Medium) or Lysine (K8)/Arginine (R10) (Heavy) amino acids for two weeks prior to the experiment. Light cells were untreated, Medium cells were treated with Phleomycin (500 μM, 1 h) and Heavy cells were treated with MLN4924 (3μM, 1 h) prior to treatment with Phleomycin (500 μM, 1 h). GFP-immunoprecipitation was performed with GFP-TRAP beads (Invitrogen) as described for the Ku ubiquitylation experiment and Ku ubiquitylation was analyzed by MS.

Identification of GFP-Ku70 interactors

RPE-1 cells stably expressing GFP and RPE-1 cells expressing Ku70 endogenously tagged with GFP were grown in SILAC medium as described above for the identification of Ku ubiquitylation sites. RPE-1-GFP were grown in Light and RPE1-GFP-Ku70 were grown in Medium or Heavy media for two weeks prior to the experiment. A full 15 cm plate of cells was treated with Phleomycin (500 μM, 1 h; Light RPE-GFP and Medium RPE-GFP-Ku70) or pre-treated with MLN4924 (3μM, 1 h) prior to Phleomycin treatment (Light RPE-GFP and Heavy

RPE-GFP-Ku70). MLN4924 treatment was added to the GFP-only cells to control for increased non-specific interactions mediated by MLN4924 itself. GFP-immunoprecipitation was performed with GFP-TRAP beads (Invitrogen) similarly as described for the Ku ubiquitylation experiment, with the exception that lysis and washes were done in 150 mM NaCl. Interactors were analyzed by LC-MS/MS.

LC-MS/MS for Ku interactors.

Precipitated proteins were resolved by SDS-PAGE and digested in-gel with trypsin. Peptide fractions were analyzed on a quadrupole Orbitrap mass spectrometer (Q-Exactive Plus, Thermo Scientific) equipped with an EASY-nLC II nanoflow HPLC system (Thermo Scientific) as described (Michalski et al. PMID: 21642640). Raw data files were analyzed using MaxQuant development version 1.3.9.21 (Cox and Mann, 2008). Parent ion and MS2 spectra were searched against a database containing 88,473 human protein sequences obtained from the UniProt knowledge base released in December 2013 using the Andromeda search engine (Cox et al., 2011). Spectra were searched with a mass tolerance of 6 ppm in MS mode, 20 ppm in HCD MS2 mode, strict trypsin specificity and allowing up to 2 missed cleavage sites. Cysteine carbamidomethylation was included as a fixed modification and N-terminal protein acetylation and methionine oxidation were included as variable modifications. For mapping of ubiquitylation sites n-ethylmaleimide modification of cysteines, di-glycine-lysine, N-terminal protein acetylation and methionine oxidation were searched as variable modifications. Site localization probabilities were determined by MaxQuant using the PTM scoring algorithm as described previously (Cox and

Mann, 2008). The dataset was filtered based on posterior error probability (PEP) to arrive at a false discovery rate of 1% for peptide spectrum matches and protein groups.

Supplementary References

Britton, S., Coates, J., and Jackson, S.P. (2013). A new method for high-resolution imaging of Ku foci to decipher mechanisms of DNA double-strand break repair. J. Cell Biol. *202*, 579–595.

Cox, J., and Mann, M. (2008). MaxQuant enables high peptide identification rates, individualized p.p.b.-range mass accuracies and proteome-wide protein quantification. Nat. Biotechnol. *26*, 1367–1372.

Cox, J., Neuhauser, N., Michalski, A., Scheltema, R. a, Olsen, J. V, and Mann, M. (2011). Andromeda: a peptide search engine integrated into the MaxQuant environment. J. Proteome Res. *10*, 1794–1805.

Emanuele, M.J., Elia, A.E.H., Xu, Q., Thoma, C.R., Izhar, L., Leng, Y., Guo, A., Chen, Y.-N., Rush, J., Hsu, P.W.-C., et al. (2011). Global identification of modular cullin-RING ligase substrates. Cell *147*, 459–474.

Galanty, Y., Belotserkovskaya, R., Coates, J., and Jackson, S.P. (2012). RNF4, a SUMO-targeted ubiquitin E3 ligase, promotes DNA double-strand break repair. Genes Dev. *26*, 1179–1195.

Giunta, S., Belotserkovskaya, R., and Jackson, S.P. (2010). DNA damage signaling in response to double-strand breaks during mitosis. J. Cell Biol. *190*, 197–207.

Higa, L., Yang, Y., Zheng, J., Banks, D., Wu, M., Ghosh, P., Sun, H., and Zhang, H. (2006). Involvement of CUL4 Ubiquitin E3 Ligases in Regulating CDK Inhibitors Dacapo/p27KIP1 and Cyclin E Degradation. Cell Cycle 71–77.

Riballo, E., Critchlow, S.E., Teo, S.H., Doherty, a J., Priestley, a, Broughton, B., Kysela, B., Beamish, H., Plowman, N., Arlett, C.F., et al. (1999). Identification of a defect in DNA ligase IV in a radiosensitive leukaemia patient. Curr. Biol. *9*, 699–702.

Toth, J.I., Yang, L., Dahl, R., and Petroski, M.D. (2012). A gatekeeper residue for NEDD8-activating enzyme inhibition by MLN4924. Cell Rep. *1*, 309–316.
