# Supplementary material for: Neddylation Promotes Ubiquitylation and Release of Ku from DNA-Damage Sites
Source: Cell Rep. 2015 Apr 23;11(5):704–14. doi: 10.1016/j.celrep.2015.03.058 (PMC4431666; doi:10.1016/j.celrep.2015.03.058)
Supplement: Document S2. Article plus Supplemental Information [file mmc4.pdf]

Cell Reports

Article

Neddylation Promotes Ubiquitylation and Release of Ku from DNA-Damage Sites

Graphical Abstract

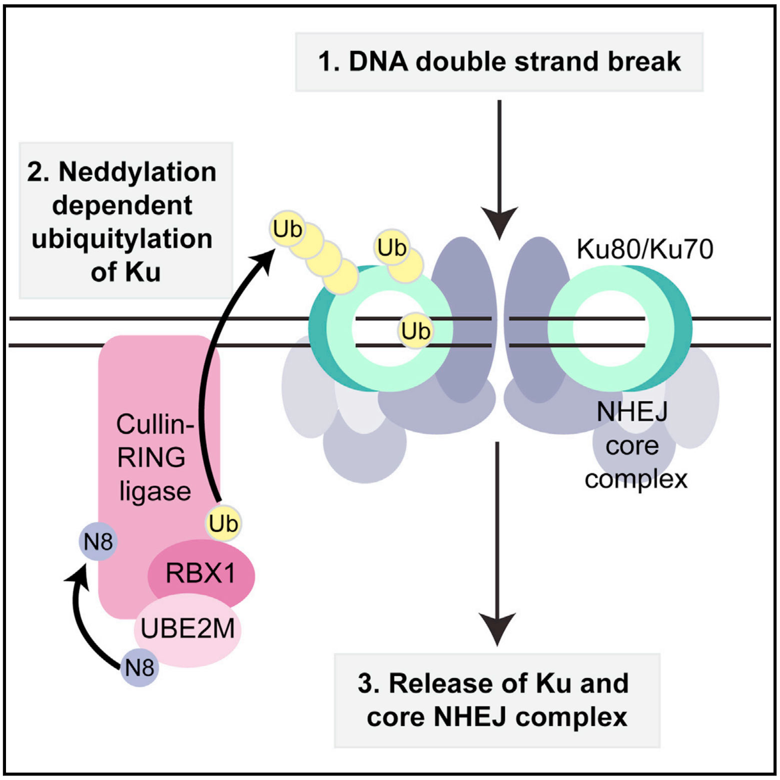

Highlights

- NEDD8 accumulation at DNA-damage sites is a dynamic process
- Depletion of the NEDD8 E2 UBE2M reduces cell survival following NHEJ
- Neddylation promotes ubiquitylation of Ku following DNA damage
- Neddylation promotes Ku release from damage sites following DNA repair

Authors

Jessica S. Brown, Natalia Lukashchuk, ..., Yaron Galanty, Stephen P. Jackson

Correspondence

p.beli@imb-mainz.de (P.B.),
y.galanty@gurdon.cam.ac.uk (Y.G.),
s.jackson@gurdon.cam.ac.uk (S.P.J.)

In Brief

Double-strand break (DSB) repair is essential for genomic stability and is tightly regulated by modification of proteins with ubiquitin and ubiquitin-like molecules. Brown et al. show that, upon completion of repair, neddylation promotes release of the main DSB sensor Ku from damaged DNA, providing insight into a long-sought mechanism.

Brown et al., 2015, Cell Reports *11*, 704–714
May 5, 2015 ©2015 The Authors
http://dx.doi.org/10.1016/j.celrep.2015.03.058

OPEN
ACCESS
CellPress

Cell Reports
Article

Neddylation Promotes Ubiquitylation and Release of Ku from DNA-Damage Sites

Jessica S. Brown,[1,4] Natalia Lukashchuk,[1,4] Matylda Sczaniecka-Clift,[1] Sébastien Britton,[1,2] Carlos le Sage,[1] Patrick Calsou,[2] Petra Beli,[3,*] Yaron Galanty,[1,*] and Stephen P. Jackson[1,*]
[1]The Wellcome Trust and Cancer Research UK Gurdon Institute, University of Cambridge, Cambridge 2 1QN, UK
[2]Institut de Pharmacologie et de Biologie Structurale, CNRS, Université de Toulouse-Université Paul Sabatier, Equipe Labellisée Ligue contre le Cancer, 31077 Toulouse, France
[3]Institute of Molecular Biology (IMB), 55128 Mainz, Germany
[4]Co-first author
*Correspondence: p.beli@imb-mainz.de (P.B.), y.galanty@gurdon.cam.ac.uk (Y.G.), s.jackson@gurdon.cam.ac.uk (S.P.J.)
http://dx.doi.org/10.1016/j.celrep.2015.03.058
This is an open access article under the CC BY license (http://creativecommons.org/licenses/by/4.0/).

SUMMARY

The activities of many DNA-repair proteins are controlled through reversible covalent modification by ubiquitin and ubiquitin-like molecules. Nonhomologous end-joining (NHEJ) is the predominant DNA double-strand break (DSB) repair pathway in mammalian cells and is initiated by DSB ends being recognized by the Ku70/Ku80 (Ku) heterodimer. By using MLN4924, an anti-cancer drug in clinical trials that specifically inhibits conjugation of the ubiquitin-like protein, NEDD8, to target proteins, we demonstrate that NEDD8 accumulation at DNA-damage sites is a highly dynamic process. In addition, we show that depleting cells of the NEDD8 E2-conjugating enzyme, UBE2M, yields ionizing radiation hypersensitivity and reduced cell survival following NHEJ. Finally, we demonstrate that neddylation promotes Ku ubiquitylation after DNA damage and release of Ku and Ku-associated proteins from damage sites following repair. These studies provide insights into how the NHEJ core complex dissociates from repair sites and highlight its importance for cell survival following DSB induction.

INTRODUCTION

The DNA-damage response (DDR), comprising the sensing, signaling, and repair of damaged DNA, requires recruitment and post-translational modification (PTM) of many proteins at DNA-damage sites (Polo and Jackson, 2011). Effective DSB repair is essential for genomic stability, with hereditary DSB repair defects causing cancer predisposition, immunodeficiency, developmental defects, and hypersensitivity to DNA damaging agents (Jackson and Bartek, 2009; Ciccia and Elledge, 2010). DSB repair mainly occurs through two pathways: homologous recombination (HR) and nonhomologous end-joining (NHEJ). Classical NHEJ requires binding of the Ku70/Ku80 heterodimer to DNA ends, with ensuing recruitment of DNA-PKcs, PAXX, and end-processing factors leading to repair by the DNA ligase IV/XRCC4/XLF complex (Davis and Chen, 2013; Grundy et al., 2014; Wang and Lees-Miller, 2013; Ochi et al., 2015; Xing et al., 2015). While the main NHEJ proteins have been characterized, it is not yet clear how their recruitment to, and dissociation from, DSBs is regulated.

The covalent attachments of ubiquitin and the ubiquitin-like molecule (UBL) SUMO to DDR proteins have well-established roles in the DDR (Jackson and Durocher, 2013). However, functions of other UBLs in such processes remain relatively unexplored (Pinder et al., 2013). Of the UBLs, NEDD8 has the highest sequence similarity to ubiquitin and is conjugated to substrates in an enzymatic process analogous to those of ubiquitin and other UBLs (Figure 1A; reviewed by Enchev et al., 2015; Lydeard et al., 2013; Schulman and Harper, 2009; Watson et al., 2011). The NEDD8 E1 activating enzyme, comprising the NAE1-UBA3 heterodimer, adenylates the exposed NEDD8 C-terminal glycine and forms a covalent NEDD8-thioester linkage. Activated NEDD8 is then conjugated to substrates, predominantly by the E2/E3 enzyme complexes UBE2M/RBX1 or UBE2F/RBX2 (Huang et al., 2009). Although RBX1 and RBX2 are the major NEDD8 E3s, others have been described (Kurz et al., 2005; Ma et al., 2013; Meyer-Schaller et al., 2009; Kurz et al., 2008; Scott et al., 2010; Xirodimas et al., 2004). De-neddylation is mainly mediated by the CSN (COP9 signalosome) complex (Cope et al., 2002). The best-characterized NEDD8 substrates, cullins (CUL1, 2, 3, 4A, 4B, 5, and 7 and PARC in human cells), serve as molecular scaffolds for cullin-RING ubiquitin ligases (CRLs; Lydeard et al., 2013; Sarikas et al., 2011). Cullin neddylation increases CRL ubiquitylation activity via conformational changes that optimize ubiquitin transfer to target proteins (Duda et al., 2008). MLN4924, a mechanism-based inhibitor of NAE1-UBA3, currently being explored as an anti-cancer treatment, blocks neddylation in cells, inhibiting CRL activity (Brownell et al., 2010; Soucy et al., 2009; Milhollen et al., 2011). While neddylation has a well-defined role in DNA nucleotide excision repair (Groisman et al., 2003), recent studies have connected it to DSB-repair processes (Cukras et al., 2014; Li et al., 2014; Ma et al., 2013; Wu et al., 2012; Jimeno et al., 2015). Here, we establish that neddylation is crucial for cell survival after DSB induction, and that it promotes Ku ubiquitylation and release from DSB sites.

CrossMark

RESULTS

Neddylation Occurs at DSB Sites

To determine whether NEDD8 is present at DNA-damage sites, we used laser microirradiation to generate DSBs in cells presensitized with bromodeoxyuridine (BrdU; Lukas et al., 2003). This revealed that both stably expressed GFP-tagged (Figure 1B) and endogenous (Figure S1A) NEDD8 were detectable at DNA-damage sites within minutes, co-localizing with Ser-139 phosphorylated histone H2AX (γH2AX), an established DSB marker (Rogakou et al., 1998). Pre-incubating cells for 1 hr with MLN4924 at a dose that effectively inhibits NEDD8 conjugation in cells (Figure S1B) blocked NEDD8 recruitment to sites of laser microirradiation (Figures 1B and S1A), indicating that DNA-damage-induced NEDD8 accrual requires an active neddylation pathway.

The ubiquitin machinery, particularly the ubiquitin E1 UBE1, can utilize highly overexpressed NEDD8, causing "false" neddylation of substrates (Hjerpe et al., 2012). Importantly, GFP-NEDD8 conjugation detected by immunoblotting of extracts from our stable cell line was blocked by MLN4924, but not by depleting UBE1 (Figure S1C). This indicated that overexpressed GFP-NEDD8 in this cell line was not substantially used by the ubiquitin system. However, depletion of UBE1 did reduce GFP-NEDD8 recruitment to DNA-damage sites (Figure S1D), although to a lesser extent than MLN4924 treatment (Figures 1B and S1D), demonstrating that NEDD8 accumulation is at least partially dependent on ubiquitylation-mediated events. Of note, NEDD8 accumulation was only observed in BrdU pretreated cells (Figure S1E), implying that in our system, NEDD8 accrual was largely promoted by DSBs rather than other forms of damage (Lukas et al., 2003). NEDD8 recruitment did not require the activity of PARP or the DDR kinases ATM, ATR, and DNA-PK (Figure S1F). Indeed, impairing DNA repair by inhibiting these kinases actually increased NEDD8 accumulation at laser sites (Figure S1F), supporting a role for neddylation in DSB-dependent events.

Although it was reported recently by Ma et al. (2013) that neddylation promotes ubiquitylation at sites of DNA damage, we found that, in our system, robust inhibition of neddylation by MLN4924 did not decrease ubiquitylation at DNA-damage sites as detected by the FK2 antibody (Figure 1B). In the Ma et al. (2013) study, neddylation was inhibited by depleting RNF111/Arkadia, which they reported to be a NEDD8 E3 ligase. However, RNF111 is also a well-established ubiquitin E3 ligase with a role in the DDR (Poulsen et al., 2013) and it was not determined by Ma et al. whether the effects they observed on ubiquitylation and other aspects of the DDR were due to the ubiquitin E3 activity, rather than the reported NEDD8 E3 ligase activity of RNF111.

Through assessing GFP-NEDD8 recruitment kinetics in live cells, we found that NEDD8 accumulated at damaged sites as early as 5 min after microirradiation and persisted until 40 min in most cells (Figure S1G, left). To further investigate neddylation dynamics, we treated cells with MLN4924 immediately before laser microirradiation. In these cells, NEDD8 was initially detected at damaged sites (5 min) and then rapidly disappeared, being undetectable by 15 min (Figure S1G, right). The initial accumulation of NEDD8 in this instance most likely represents the time taken for neddylation to be completely inhibited in cells by MLN4924 (which occurs within 5 min; Brownell et al., 2010). These data therefore suggested that neddylation is a dynamic modification that occurs and turns over at DSBs, although we cannot exclude the possibility that some pre-neddylated proteins accumulate at damaged sites then disperse. To corroborate our findings, we tested for DNA-damage-dependent recruitment of neddylation-pathway components. Crucially, this revealed that the NEDD8-conjugating E2 enzymes UBE2M and UBE2F (Figure 1C), and the deneddylating-complex catalytic subunit, CSN5 (Figure 1D), were recruited to DNA-damage sites with kinetics similar to that of GFP-NEDD8. Furthermore, CSN5 recruitment was blocked by MLN4924 (Figure 1D), implying that neddylation is required for CSN5 recruitment. Collectively, these data strongly supported a model in which neddylation and deneddylation actively occur at DSB sites.

Neddylation Promotes Cell Survival after NHEJ

In light of the above findings and because inhibiting neddylation can sensitize cells to DNA-damaging agents (Kee et al., 2012; Wei et al., 2012; Yang et al., 2012; Garcia et al., 2014), we hypothesized that neddylation promotes DSB repair. To investigate this, we tested the effects of depleting UBE2M or UBE2F by small interfering RNAs (siRNAs) on clonogenic cell survival following ionizing radiation (IR) treatment. Notably, while both UBE2M and UBE2F were recruited to DNA-damage sites (Figure 1C), UBE2M but not UBE2F depletion significantly sensitized cells to IR (Figures 1E and S1H). We speculate that functional compensation by UBE2M (and potentially lower levels of UBE2F compared to UBE2M in the cells we tested) may explain why UBE2F was recruited to laser lines but its depletion did not sensitize cells to IR.

Although DSB repair by HR is restricted to S and G2 cells and can take several hours to complete (Shibata et al., 2011), NHEJ occurs in all cell-cycle stages, with most simple breaks being repaired within minutes (Wang et al., 2001; DiBiase et al., 2000). Because NEDD8 accrual at DNA-damage sites was rapid and occurred in most cells, we speculated that neddylation might regulate NHEJ. In accord with this, depleting UBE2M with two independent siRNAs significantly reduced the number of cell colonies arising in an assay for random plasmid integration (Figure 1F), which is mediated by NHEJ as well as alternative DNA end-joining processes.

Neddylation Promotes Ku Release from DNA Damage Sites

To explore the impact of neddylation on NHEJ, we used high-resolution microscopy together with an RNase A-based extraction method to study formation and dissolution of Ku IR-induced foci (IRIF). In agreement with published findings (Britton et al., 2013), Ku foci in control cells were formed within 8 min following IR and then decayed over time, returning to near baseline levels by 1 hr (Figures 2A and 2B). Strikingly, while not impairing Ku IRIF formation, MLN4924 treatment significantly delayed their dissolution, with high numbers of Ku foci remaining even after 2 hr (Figures 2A and 2B). This effect was not through MLN4924 itself causing DNA damage because parallel treatments of non-irradiated cells with MLN4924 did not induce Ku IRIF or γH2AX

OPEN ACCESS
CellPress

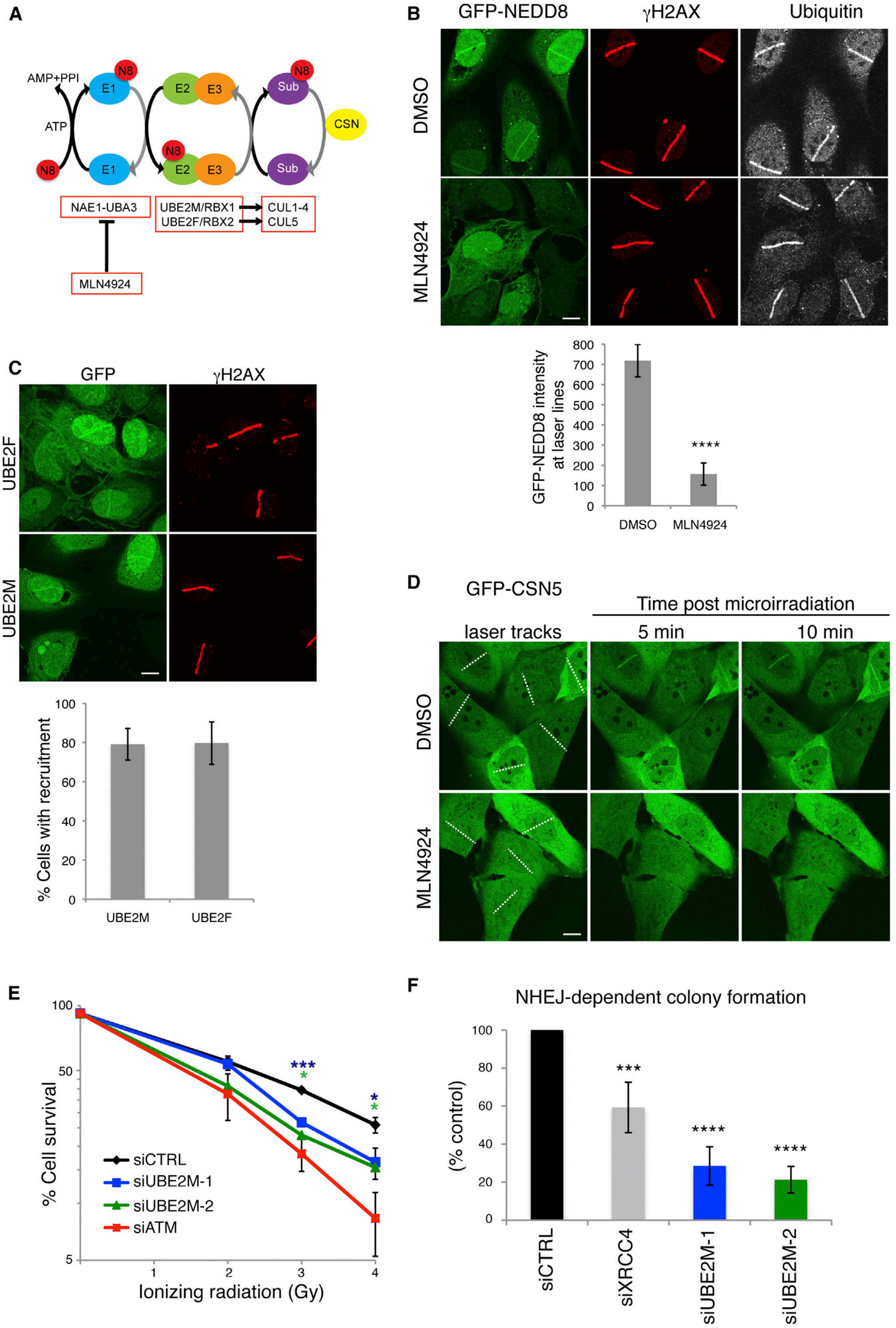

(legend on next page)

(Figure S2A; MLN4924 treatment for longer than 6 hr did cause DNA damage as previously described by Soucy et al., 2009). Of note, while Feng and Chen (2012) published that RNF8 depletion caused Ku80 retention at laser microirradiation sites, we were unable to detect any effect of RNF8 depletion on the resolution of Ku IRIF (data not shown).

To test whether the effect of MLN4924 on Ku removal was indeed via UBA3 inhibition, we generated U2OS cell lines stably expressing wild-type UBA3 or a UBA3 Ala-171 to Thr mutant (UBA3-A171T) that confers MLN4924 resistance (Toth et al., 2012; Milhollen et al., 2012). As expected, NEDD8 conjugation was abolished by MLN4924 in cells expressing wild-type UBA3 but not UBA3 A171T (Figures 2C and S2B). Importantly, while both cell lines showed comparable Ku IRIF kinetics under control conditions (Figure 2D), MLN4924 caused persistent Ku IRIF only in cells expressing wild-type UBA3 (Figure 2D), thus indicating that the effect of MLN4924 on Ku was via UBA3 inhibition. Although we initially considered the possibility that Ku IRIF persistence reflected defective DSB repair, this did not appear to be the case because MLN4924 did not impair the time-dependent reduction of IR-induced γH2AX, a well-established readout of DSB repair (Britton et al., 2013; Löbrich et al., 2010), detected either by immunoblotting (Figure S2C) or immunofluorescence microscopy (Figures 2A and 2E; note in Figure 2E that γH2AX did persist following DNA-PK inhibition). During these analyses, we found that the size and intensity of Ku foci were unaffected by MLN4924, indicating that MLN4924 does not lead to more Ku molecules being loaded onto each DSB (data not shown). Collectively, these findings suggested that blocking neddylation does not affect Ku loading but rather impairs Ku removal from damage sites after repair has occurred.

To assess the above model by a different approach, we used immunoblotting to monitor the accumulation of Ku and other NHEJ factors in RNase A-resistant chromatin fractions after treating cells with the radiomimetic compound phleomycin. In accord with our immunofluorescence data, inhibiting neddylation with MLN4924 caused Ku80 and Ku70 persistence on chromatin after treating cells with a pulse of phleomycin (Figure 3A). Similarly, MLN4924 caused persistence of the NHEJ factors XRCC4, LIG4, and XLF, suggesting that they are recruited and subsequently released concomitantly with Ku (Figure 3A; as shown on the right, total levels of Ku80, XRCC4, LIG4, and XLF were unaltered by DNA damage and/or MLN4924). These data supported a model in which neddylation promotes removal of Ku and other NHEJ factors from DNA-damage sites.

Proteomics Identifies Neddylation-Dependent Ku Interactors

To identify factors that might associate with Ku in a NEDD8-pathway-dependent manner, we used human RPE-1 cells stably expressing GFP or RPE-GFP-Ku70 cells expressing endogenously tagged GFP-Ku70, wherein the GFP-tag was fused to one of the *XRCC6* chromosomal alleles (Britton et al., 2013), in SILAC (stable isotope labeling of amino acids in cell culture) studies followed by liquid chromatography-tandem mass spectrometry (LC-MS/MS; Figure 3B; Table S1). Applying a cutoff of ≥2-fold enrichment for Ku-specific binding, we identified several known Ku interactors as well as various other proteins, including CUL4A (Figure 3B; a highly related cullin CUL4B had a ratio of 1.8). Subsequent reciprocal co-immunoprecipitation studies confirmed CUL4A as a Ku interactor (Figures 3C and S3A). Notably, we found that depletion of either CUL4A or CUL4B significantly reduced NEDD8 accrual at DNA-damage sites (Figure S3B), and stably expressed GFP-CUL4A and GFP-CUL4B were both recruited to DSB sites (Figure S3C). To investigate their potential functional roles in Ku release from chromatin following DNA repair, we depleted CUL4A/CUL4B by siRNA and established cell lines stably expressing inducible dominant-negative CUL4A or CUL4B. However, by neither of these approaches were we able to demonstrate consistently strong effects on Ku removal (data not shown). Nevertheless, we also noted that neither approach inhibited CRL4 ubiquitylation activity to a level comparable to MLN4924 treatment, as monitored by protein levels of the CRL4 substrates CDT1, p27, and p21 (Figures S3D and S3E). We therefore concluded that residual CRL enzymatic activity and/or functional redundancy between CUL4A, CUL4B, and probably other cullins likely precluded us from observing effects in these studies. Indeed, in vitro studies have implicated CUL1 in the removal of Ku from DNA in cell-free *Xenopus laevis* egg extracts (Postow et al.,

Figure 1. NEDD8 and the Neddylation Machinery Accumulate at Sites of DNA Breaks and Promote Cell Survival after NHEJ

(A) Representation of major neddylation pathway components. NEDD8 (N8) is conjugated in an ATP-dependent cascade involving an E1 (NAE1-UBA3), E2 (UBE2M or F), and E3 (RBX1 or 2) to Cullin substrates (Sub). Neddylation is reversed by the CSN complex. MLN4924 inhibits UBA3. Figure adapted from Brown and Jackson (2015).

(B) MLN4924 blocks NEDD8, but not ubiquitin recruitment to DNA-damage sites. U2OS-GFP-NEDD8 cells were pre-treated for 1 hr with DMSO or 3 μM MLN4924 and laser microirradiated. Cells were fixed after 20 min and visualized by immunofluorescence as indicated. Graph shows average intensity of GFP-NEDD8 at the laser line from three experiments ±SD. White bar represents 10 μM. Asterisks indicate statistically significant difference to control (*p < 0.05; **p < 0.01; ***p < 0.001; ****p ≤ 0.0001).

(C) GFP-UBE2F and GFP-UBE2M are recruited to DNA-damage sites. U2OS cells stably expressing GFP-UBE2F or GFP-UBE2M were laser microirradiated, fixed, and visualized as in (B). Graph shows average percentage of γH2AX positive cells with detectable GFP-UBE2M or GFP-UBE2F recruitment from five independent experiments ±SD. White bar represents 10 μM.

(D) GFP-CSN5 recruitment to DNA-damage sites is blocked by MLN4924. U2OS cells stably expressing GFP-CSN5 were treated as in (B). Images were acquired by live cell imaging. Laser tracks are indicated by dashed white lines. White bar represents 10 μM

(E) UBE2M depletion causes hypersensitivity to IR. Clonogenic U2OS cell survivals were performed after transfection with indicated siRNAs and doses of IR. Each point represents an average of at least three independent experiments (except UBE2M-2 which was repeated twice). Error bars correspond to SDs, and asterisks are as in (B).

(F) UBE2M depletion causes an NHEJ defect. Random plasmid integration assay was performed in U2OS cells transfected with indicated siRNAs. Error bars correspond to SD of at least three independent experiments (asterisks as in B). See also Figure S1.

OPEN
ACCESS
CellPress

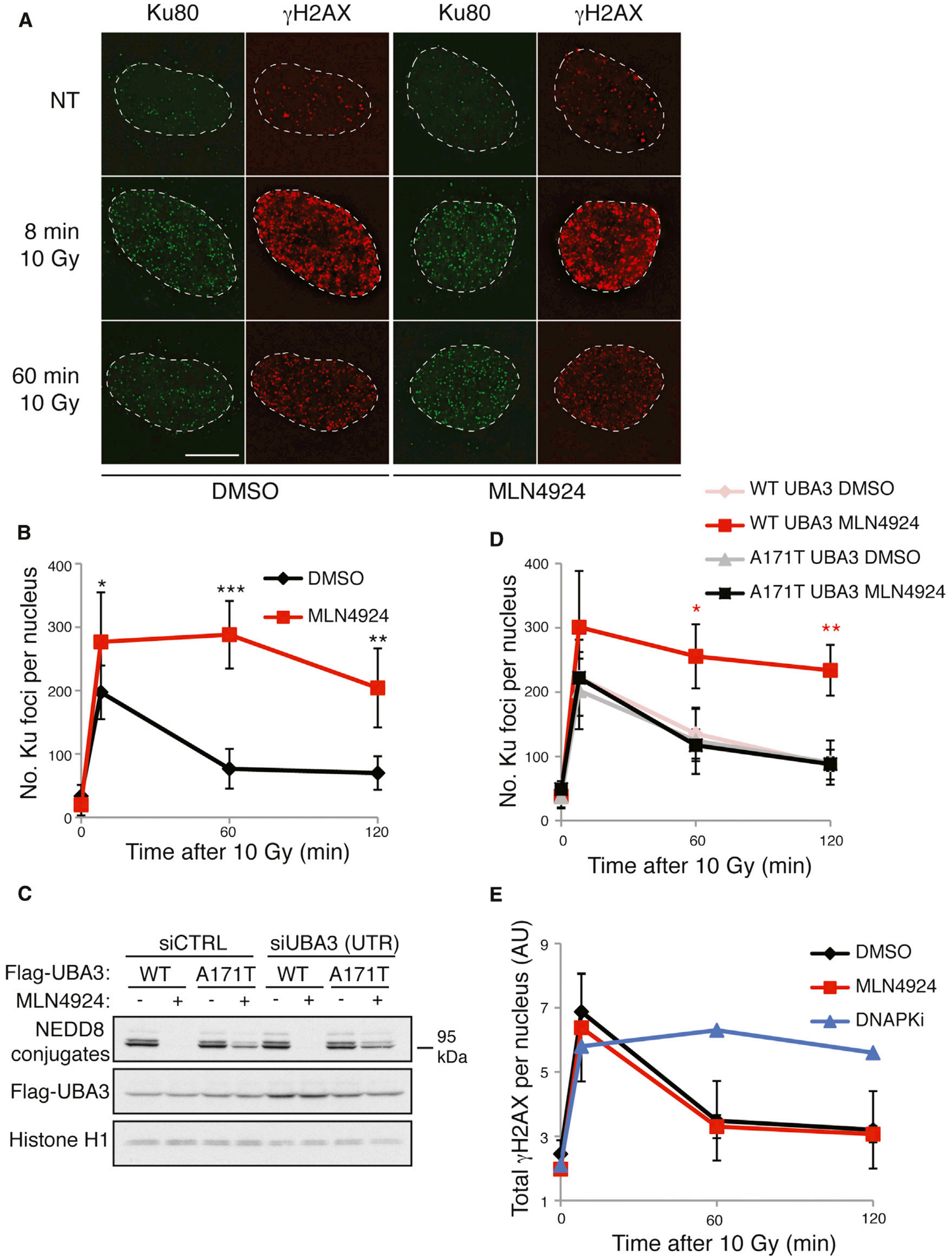

Figure 2. MLN4924 Inhibits Ku Removal from DNA Repair Sites

(A and B) MLN4924 causes Ku80 foci persistence after IR. U2OS cells were pre-treated with DMSO or 3 μM MLN4924 for 1 hr and then subjected to 10 Gy IR. Samples were pre-extracted with CSK+RNase A and visualized by immunofluorescence. (A) shows representative images, and (B) shows quantification. Dotted lines indicate nuclear peripheries. Error bars correspond to SDs of at least three independent experiments (asterisks as in Figure 1B). White bar represents 10 μM.

(C) U2OS-A171T UBA3 are resistant to MLN4924. U2OS cells stably expressing WT UBA3 or A171T UBA3 were treated with DMSO or 3 μM MLN4924 for 1 hr and analyzed by immunoblotting with indicated antibodies. Endogenous UBA3 was depleted with a siRNA to the 3′ UTR (Figure S2B). Neddylated conjugates are detected with a NEDD8-specific antibody.

(legend continued on next page)

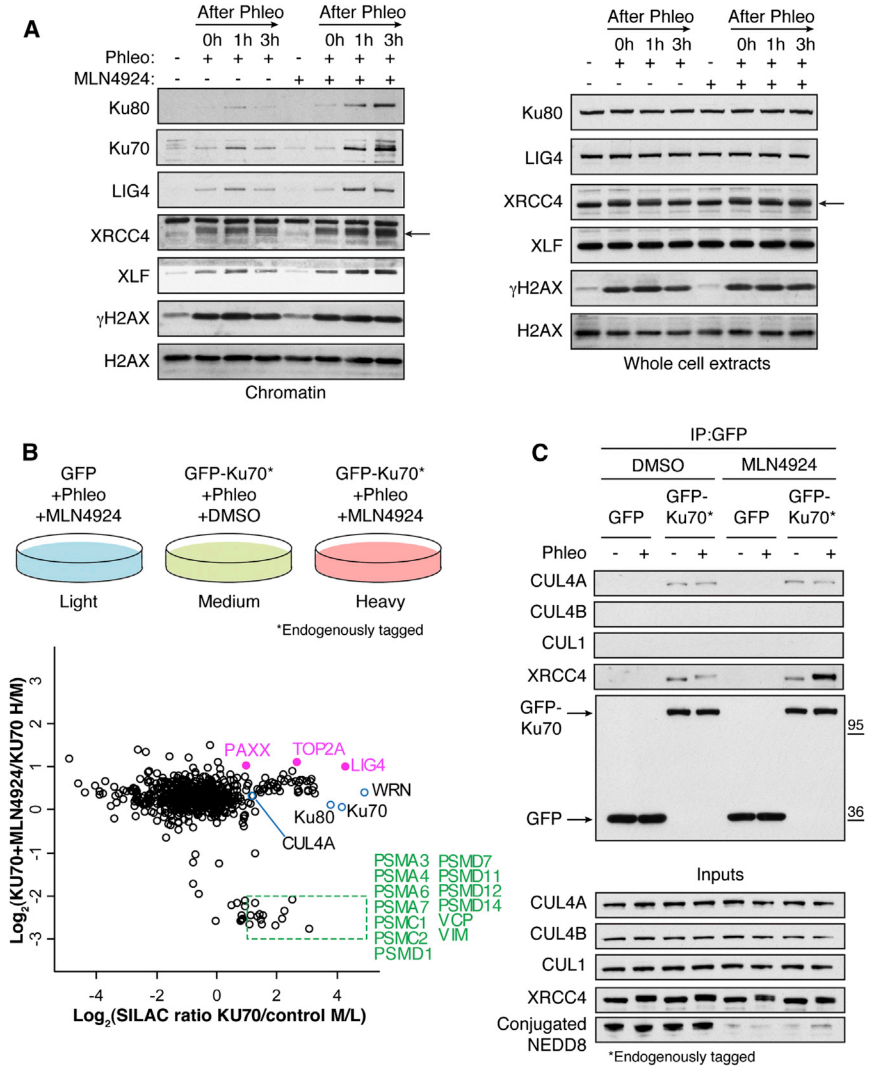

Figure 3. Proteomics Identifies Neddylation-Dependent Ku Interactors

(A) MLN4924 causes retention of NHEJ factors on the chromatin. U2OS cells were pretreated with DMSO or 3 μM MLN4924 for 1 hr and then treated with 500 μM phleomycin (Phleo) for 1 hr. Cells were left to recover in the presence of MLN4924 or DMSO following phleomycin removal and then collected at the indicated times. Cells were pre-extracted with CSK buffer + RNase A prior to lysis (chromatin; left) or lysed as whole cell extracts (right) and immunoblotted with indicated antibodies. Black arrow indicates XRCC4.

(B) RPE-1 cells stably expressing GFP or Ku70 endogenously tagged with GFP were labeled with light, medium, or heavy isotopes and treated as indicated. Cell lysates were subjected to GFP retrieval. Enriched proteins were resolved by SDS-PAGE and proteolysed in gel with trypsin, and peptides were analyzed by LC-MS/MS. The scatterplot shows the logarithmized SILAC ratio of GFP-KU70/GFP control and GFP-KU70 + MLN4924/GFP-KU70. The known Ku interactors and CUL4A (ratio 2.23) are labeled in black font and open blue circles. In pink are interactions enhanced upon MLN4924. In green are interactions decreased upon MLN4924 (see also Table S1).

(C) Experiment repeated as in (B) without isotope labeling of cells. Following GFP IP, cell lysates were immunoblotted with indicated antibodies. Note that CUL1 (Postow and Funabiki, 2013) and CUL4B were not detected in Ku immunoprecipitates. See also Figure S3.

2008; Postow and Funabiki, 2013), thus indirectly supporting the idea that Ku could be a shared substrate of CUL1 and CUL4A/B in human cells. In regard to the above, we found that depletion of RBX1—which functions together with UBE2M (Huang et al., 2009) and is the NEDD8 and ubiquitin E3 ligase for cullins 1, 2, 3, 4A, and 4B—increased the number of Ku IRIF at all time points tested and caused persistence of Ku and NHEJ factors on chromatin after phleomycin treatment (Figures S3F–S3H). These effects on NHEJ-factor kinetics were less marked than with MLN4924 treatment, however, and also the kinetics of Ku release following RBX1 depletion differed from that seen with MLN4924 treatment. These differences might reflect the potency of MLN4924 treatment compared to incomplete RBX1 depletion (Figure S3H) and/or could be influenced by prolonged RBX1 depletion over 72 hr compared to 1 hr exposure to MLN4924. Collectively, these data were consistent with CRL activity promoting Ku removal, although we acknowledge that other factors might also be involved.

In support of a model in which neddylation promotes dissociation of the NHEJ apparatus (Figure 3A), our proteomics data and subsequent co-immunoprecipitation studies revealed that the interaction between Ku and DNA ligase 4/XRCC4, as well as the recently identified NHEJ complex component PAXX (Ochi et al., 2015; Xing et al., 2015), was significantly enhanced when neddylation was blocked by MLN4924 (Figures 3B and 3C; Table S1). Interestingly, the interaction between Ku and several other proteins, including topoisomerase 2A (TOP2A), was also enhanced when neddylation was blocked by

(D) MLN4924 effects on Ku80 foci retention are through UBA3 inhibition. U2OS cells stably expressing WT UBA3 or A171T UBA3 were processed as in (A), and results were quantified as in (B).

(E) MLN4924 does not affect γH2AX recovery after IR. Quantification of total γH2AX intensity per nucleus in cells treated with 10 Gy IR then harvested at indicated times. Samples were prepared as in (A). Pre-treatment for 1 hr with 3 μM DNA-PK inhibitor (DNA-PKi) used as a positive control. Statistical analysis as in (B). AU, arbitrary units. See also Figure S2.

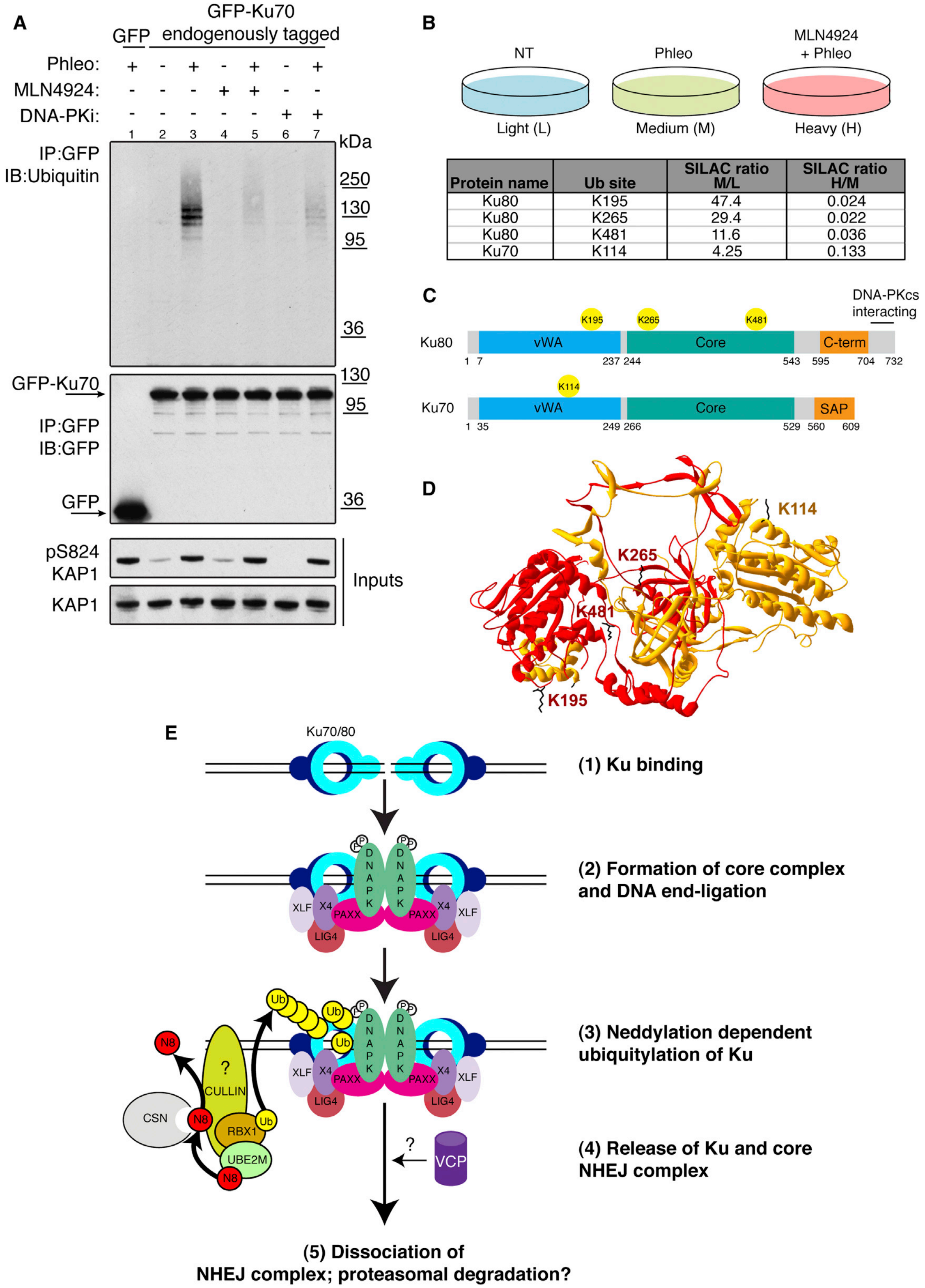

Figure 4. MLN4924 Blocks Ku Ubiquitylation after DNA Damage

(A) In vivo ubiquitylation assay. RPE-1 cells expressing Ku70 endogenously tagged with GFP (lanes 2–7) or RPE-1 cells stably expressing GFP (lane 1) were pre-treated with DMSO, 3 μM MLN4924, or 3 μM DNA-PK inhibitor for 1 hr prior to treatment with 500 μM phleomycin (Phleo) for 1 hr as indicated. Cell lysates were

(legend continued on next page)

MLN4924 (Figure 3B; Table S1). This could occur because such factors directly interact with Ku on the chromatin and are therefore released with Ku, or, alternatively, these factors might interact with chromatin in other ways, in a manner that is regulated by neddylation. Strikingly, almost all the proteins whose association with Ku was diminished upon MLN4924 treatment comprised factors associated with the 26S proteasome, as well as the segregase/unfoldase VCP (valosin containing protein; also known as p97) that targets ubiquitylated proteins to dissociate them from molecular assemblies, frequently promoting their proteasomal degradation (Meerang et al., 2011). Notably, MLN4924 treatment did not affect the levels of VCP or proteasome components (Figure S3I). Collectively, these findings suggested that VCP and proteasomal components recognize DNA-damage-dependent, NEDD8-mediated ubiquitylation of Ku, and/or other NHEJ components.

Neddylation Promotes Ku Ubiquitylation following DNA-Damage Induction

In light of the above findings, we tested whether neddylation might promote ubiquitylation of Ku. Thus, we immunoprecipitated, under stringent conditions (1 M NaCl; see Experimental Procedures), endogenous Ku70 from RPE-GFP-Ku70 cells (Britton et al., 2013). Subsequent immunoblotting revealed that Ku ubiquitylation was markedly increased by phleomycin treatment (Figure 4A, lanes 2 and 3), whereas no ubiquitylated species were detected in cells expressing GFP alone (lane 1). Furthermore, this ubiquitylation did not occur with DNA-damaging agents that do not directly yield DSBs (Figure S4A). Crucially, inhibiting neddylation with MLN4924 strongly reduced Ku ubiquitylation induced by phleomycin treatment (Figure 4A, lanes 4 and 5; note in Figure S4B that overall ubiquitylation in cells was not affected by MLN4924). Blocking NHEJ-mediated DSB repair with a DNA-PK inhibitor, which has been shown to inhibit Ku release from DNA-damage sites (Britton et al., 2013), also impaired phleomycin induced Ku ubiquitylation (Figure 4A, lanes 6 and 7), as did siRNA depletion of DNA ligase 4 (data not shown), suggesting that Ku ubiquitylation occurs as a consequence of DSB repair. Although Ma et al. (2013) suggested that RNF168 recruitment to DNA-damage sites is neddylation dependent, we found that RNF168 depletion did not affect Ku ubiquitylation (Figure S4C), nor did it enhance Ku, XRCC4, or XLF persistence on chromatin after treating cells with phleomycin (Figure S4D; see Figure S4E for RNF168 depletion).

To establish whether the ubiquitylation we observed above was occurring specifically on Ku, we analyzed ubiquitylation in GFP-Ku70 immunoprecipitates by SILAC-based LC-MS/MS. This identified three sites (K195, K265, and K481) on Ku80 and one (K114) on Ku70, upon which ubiquitylation was increased following phleomycin treatment (SILAC ratio M/L) and was blocked by MLN4924 pretreatment (SILAC ratio H/M; Figure 4B; Table S2; the mass spectrum of K481 is shown in Figure S4F as an example). We investigated whether the sites identified on Ku80 were required for damage-dependent ubiquitylation of Ku and release of Ku from DNA-damage sites. This established that Ku ubiquitylation in the context of an exogenously expressed Ku80 mutant, with lysines 195, 265, and 481 (3K-R) mutated to arginine, still occurred following DNA-damage (data not shown). In addition, we found that the mutant form of Ku was still recruited and released from DNA damaged chromatin with kinetics similar to those of the wild-type protein (data not shown). These data suggested that there may be functional redundancy between the mapped sites and further, as yet unidentified ubiquitylation sites on Ku80 and or Ku70. To try to address this issue, we generated a U2OS cell line expressing an inducible mutant of Ku80 with all but one lysine mutated to arginine (we excluded K265, which has been shown to make direct contact with the DNA and is therefore likely to be important for DNA binding; Walker et al., 2001). Unfortunately, this all-but-one lysine mutant protein was not recruited to DNA-damage sites (data not shown) and could not therefore be used for further experiments. We did not attempt to generate cell lines expressing inducible mutants of Ku80 in combination with lysine-to-arginine mutants of Ku70, and it is possible that dimerization with endogenous Ku70 is sufficient to enable ubiquitylation and release of Ku complexes containing the Ku80 3K-R protein.

DISCUSSION

We have shown that neddylation is a dynamic modification at DNA-damage sites and that neddylation promotes cell survival after DSB induction. Furthermore, we have established that neddylation promotes the ubiquitylation of Ku upon DNA repair, and that this is associated with the release of Ku and other NHEJ factors from repair sites. Significantly, our work has identified DNA-damage induced, neddylation-dependent ubiquitylation of K195, K265, and K481 in Ku80 and K114 in Ku70. Interestingly, K265 and K481 lie within the core DNA binding domain of Ku80, with K265 directly making contact with DNA (Walker

immunoprecipitated (IP) with GFP-specific antibody and immunoblotted (IB) with indicated antibodies. GFP-Ku70 IP was done under stringent conditions (see Experimental Procedures). Black arrows indicate GFP-Ku70 and GFP. Phosphorylated Ser824 of KAP1 is used as a DNA-damage marker.

(B) Identification of Ku ubiquitylation sites by quantitative LC-MS/MS. RPE-1 cells stably expressing Ku70 endogenously tagged with GFP were labeled with light, medium, or heavy SILAC isotopes and treated as indicated. Ku70 was enriched with GFP-Trap agarose under stringent washing conditions. Enriched proteins were resolved by SDS-PAGE and proteolysed in gel with trypsin. Peptides were extracted from gel and analyzed by LC-MS/MS. SILAC ratio M/L >2 represents induction upon DNA damage; SILAC ratio H/M < 0.5 represents inhibition by MLN4924 (see also Table S2).

(C) Schematic representation of Ku70 and Ku80 domains with neddylation-dependent ubiquitylation sites identified in (B). vWA, von Willebrand factor A; C-term, C terminus; SAP, SAF-A/B, Acinus, and PIAS.

(D) Positions of DNA-damage-induced neddylation-dependent ubiquitylation sites in the context of the structure of the Ku heterodimer (ID:1JEQ). Ku70 and Ku80 are in orange and red, respectively, and the ubiquitylated side chains are in black.

(E) Model. (1, 2) Ku and the NHEJ complex are recruited to sites of DSBs. (3) Neddylation-dependent ubiquitylation of Ku following completion of DNA repair. (4) Ku and NHEJ factors are released from sites of DNA damage. VCP might target ubiquitylated Ku to proteasome for degradation. N8, NEDD8; Ub, ubiquitin; P, phosphorylation (see text for details). See also Figure S4.

OPEN ACCESS
CellPress

et al., 2001), while K114 and K195 lie within the von Willebrand factor (vWF) A domains (Grundy et al., 2013) of Ku70 and Ku80, respectively, that are thought to mediate protein-protein interactions (Figures 4C and 4D). The locations of these ubiquitylation sites suggest how Ku ubiquitylation on these and other sites could both trigger the dissociation of Ku from other NHEJ proteins as well as being associated with its release from DNA.

Collectively, the available data suggest the following model (Figure 4E): first, following DSB induction, Ku, PAXX, DNA-PK, XRCC4, LIG4, and XLF assemble at the DNA-damage site (Davis and Chen, 2013; Ochi et al., 2015; Xing et al., 2015); next, following DNA repair, Ku is ubiquitylated in a DNA-damage- and neddylation-dependent manner to promote the release of Ku and other NHEJ factors from the site of repair. Significantly, our proteomics analyses revealed that VCP and various proteasome subunits interact with Ku in a DNA-damage induced and neddylation-dependent manner. It is known that VCP can unfold ubiquitylated proteins and is important for extracting certain DNA-repair proteins from chromatin (Dantuma et al., 2014), and, while a role for VCP in removing Ku from DNA has been proposed (Postow, 2011), it has not yet been demonstrated in the literature. Because it has been reported that Ku removal from damage sites is not affected by proteasome inhibition (Postow et al., 2008), we suggest that in addition to disrupting interactions between Ku, DNA, and other NHEJ components, Ku ubiquitylation likely promotes targeting by VCP, leading to extraction of Ku from chromatin, perhaps then followed by proteasome-dependent Ku degradation (Figure 4E). As the Ku70/Ku80 heterodimer forms a highly stable ring structure encircling DNA ends (Walker et al., 2001), if Ku remains DNA bound once a DSB has been repaired, it would likely interfere with various processes, particularly transcription and replication (Frit et al., 2000; Ono et al., 1996). By serving as a barrier to complete genome replication and/or segregation, the persistence of Ku and other NHEJ factors on repaired DNA could thus account for the decreased cell survival we have observed when neddylation is abrogated after DSB induction as well as reduced NHEJ-dependent cell colony formation in plasmid integration assays. We recognize, however, that neddylation might also regulate several DSB repair pathways and that there are likely to be multiple mechanisms accounting for IR hypersensitivity upon MLN4924 treatment. Finally, we note that because NEDD8 pathway components are overexpressed or mutated in many human cancers, NEDD8-pathway inhibition is a promising anti-cancer strategy (Watson et al., 2011). Accordingly, our findings highlight opportunities for combining MLN4924 with DSB-inducing agents and for exploring cancer-genetic backgrounds where this combination might be particularly effective.

EXPERIMENTAL PROCEDURES

For more details on experimental procedures, please refer to the Supplemental Experimental Procedures.

DNA Damage and Drug Treatments

Cells were preincubated with inhibitors for 1 hr prior to genotoxic treatments, and MLN4924 (Active Biochem) was used at 3 μM unless otherwise indicated. ATMi (KU55933) and DNA-PKi (NU7441; Tocris Bioscience) were used at 10 and 3 μM, respectively. PARPi (olaparib; Stratech Scientific) was used at 10 μM. ATRi (ATR-45; OSUCCC Medicinal Chemistry, Ohio State University) was used at 1 μM. X-ray irradiation was performed with a calibrated irradiation system (Cell Rad Faxitron) fitted with an aluminum filter for soft X-rays. Cells were irradiated in culture medium at room temperature, and standard, 10-Gy irradiation required an exposure time of 3 min 36 s. Phleomycin (Melford Labs) was used at 500 μM for 1 hr, Carboplatin (Sigma-Aldrich) was used at 100 μM for 1 hr, and Camptothecin (Sigma-Aldrich) was used at 1 μM for 1 hr. Cells were UV-irradiated with 10 J/m^2 and analyzed 1 hr after.

Detection of Ku

For detection of Ku on chromatin and Ku IRIF, cells were processed as described previously (Britton et al., 2013).

Laser Microirradiation and Immunofluorescence

Laser microirradiation of cells and immunofluorescence were as previously described (Galanty et al., 2012).

In Vivo Ubiquitylation of Ku

RPE-1 cells endogenously tagged with GFP-Ku70 at one chromosomal allele (Britton et al., 2013) grown in 10-cm plates were pretreated with DMSO or MLN4924 (3 μM, 1 hr) and then treated with Phleomycin (500 μM, 1 hr) and lysed in a lysis buffer (20 mM Tris [pH 7.5], 40 mM NaCl, 2 mM $MgCl_2$, 10% glycerol, 0.5% NP-40) containing benzonase 18 U (Novagen) and supplemented with EDTA-free protease inhibitors (Roche) at room temperature. Lysates were adjusted to 0.5 M NaCl and incubated on ice for 30 min and cleared of debris by centrifugation at 4°C at 21130 relative centrifugal force (rcf). IP of GFP-tagged Ku70 was carried out with GFP-trap agarose beads (ChromoTek) for 2 hr at 4°C. Beads were washed four times in lysis buffer containing 1 M NaCl and subjected to SDS-PAGE and immunoblotting.

Clonogenic Survival Assay

Cells were seeded at low density, in triplicate, at two dilutions, in 6-well plates and treated with IR after 24 hr. Cells were left to recover at 37°C for 10–14 days to allow colony formation. Cells were stained with 0.5% crystal violet/20% ethanol and counted. Results were normalized to plating efficiencies.

Random Plasmid Integration Assay

Assays were performed as previously described (Galanty et al., 2012).

Statistical Analysis

When required, an unpaired Student's t test was calculated using GraphPad software (www.graphpad.com). Quantifications are based on at least three independent experiments unless otherwise specified. In all figures, significant differences between specified pairs of conditions, as judged by the t test, are highlighted by asterisks (*$p < 0.05$; **$p < 0.01$; ***$p < 0.001$; ****$p \leq 0.0001$).

SUPPLEMENTAL INFORMATION

Supplemental Information includes Supplemental Experimental Procedures, four figures, and five tables and can be found with this article online at http://dx.doi.org/10.1016/j.celrep.2015.03.058.

AUTHOR CONTRIBUTIONS

J.S.B., N.L., and S.P.J. conceived and designed experiments. Y.G. and S.B. contributed to experimental design and set up. J.S.B., N.L., M.S.-C., S.B., and C.I.-S. performed experiments. P.B. performed mass spectrometry and analyzed the results. J.S.B., N.L., and S.P.J. wrote the manuscript. M.S.-C. and S.B. should be regarded as joint second authors. All authors contributed to the discussion of results and manuscript corrections.

ACKNOWLEDGMENTS

We thank Thimo Kurz (University of Dundee, UK) for providing MLN4924 and Kate Dry, Rimma Berlotserkovskaya (S.P.J.'s laboratory), and Eric Lightcap

(Takeda Pharmaceuticals) for critical reading of the manuscript. We thank Sylvie Urbe and Michael Clague (University of Liverpool, UK) for providing the GFP-CSN5 plasmid, the Division of Signal Transduction Therapy (University of Dundee, UK) for providing UBE2M and UBE2F plasmids, Matthew Petroski (Sanford-Burnham Medical Research Institute, US) for providing FLAG-UBA3 wild-type (WT) and FLAG-UBA3-A171T constructs, and Nico Dantuma (Karolinska Institute, Sweden) and Changshun Shao (Rutgers University) for providing CUL4A and CUL4B plasmids, respectively. We also thank Nicola Lawrence, Alex Sossick, and Richard Butler (Gurdon Institute, Cambridge, UK) for help with microscopy, Volocity, and Fiji. Research in the S.P.J.'s laboratory is funded by Cancer Research UK programme grant C6/A11224, the European Research Council, and the European Community Seventh Framework Programme grant agreement no. HEALTH-F2-2010-259893 (DDResponse). Core funding is provided by CRUK (C6946/A14492) and the Wellcome Trust (WT092096). S.P.J. receives his salary from the University of Cambridge, UK, supplemented by CRUK. N.L. is funded by CRUK programme grant C6/A11224, J.S.B. is funded by a Wellcome Trust Clinical Fellowship (WT083416), and Y.G. and M.S.-C. are funded by European Research Council grant DDREAM. S.B. was funded by an EMBO long-term fellowship ALTF 93-2010, Cancer Research UK, and a post-doctoral grant from Ligue Nationale Contre le Cancer. P.B. is supported by the Emmy Noether Programme of the German Research Foundation (DFG, BE 5342/1-1).

Received: June 9, 2014
Revised: November 12, 2014
Accepted: March 25, 2015
Published: April 23, 2015

REFERENCES

Britton, S., Coates, J., and Jackson, S.P. (2013). A new method for high-resolution imaging of Ku foci to decipher mechanisms of DNA double-strand break repair. J. Cell Biol. *202*, 579–595.

Brown, J.S., and Jackson, S.P. (2015). Ubiquitylation, neddylation and the DNA damage response. Open Biol. *5* http://dx.doi.org/10.1098/rsob.150018.

Brownell, J.E., Sintchak, M.D., Gavin, J.M., Liao, H., Bruzzese, F.J., Bump, N.J., Soucy, T.A., Milhollen, M.A., Yang, X., Burkhardt, A.L., et al. (2010). Substrate-assisted inhibition of ubiquitin-like protein-activating enzymes: the NEDD8 E1 inhibitor MLN4924 forms a NEDD8-AMP mimetic in situ. Mol. Cell *37*, 102–111.

Ciccia, A., and Elledge, S.J. (2010). The DNA damage response: making it safe to play with knives. Mol. Cell *40*, 179–204.

Cope, G.A., Suh, G.S., Aravind, L., Schwarz, S.E., Zipursky, S.L., Koonin, E.V., and Deshaies, R.J. (2002). Role of predicted metalloprotease motif of Jab1/Csn5 in cleavage of Nedd8 from Cul1. Science *298*, 608–611.

Cukras, S., Morffy, N., Ohn, T., and Kee, Y. (2014). Inactivating UBE2M impacts the DNA damage response and genome integrity involving multiple cullin ligases. PLoS ONE *9*, e101844.

Dantuma, N.P., Acs, K., and Luijsterburg, M.S. (2014). Should I stay or should I go: VCP/p97-mediated chromatin extraction in the DNA damage response. Exp. Cell Res. *329*, 9–17.

Davis, A.J., and Chen, D.J. (2013). DNA double strand break repair via non-homologous end-joining. Transl. Cancer Res. *2*, 130–143.

DiBiase, S.J., Zeng, Z.C., Chen, R., Hyslop, T., Curran, W.J., Jr., and Iliakis, G. (2000). DNA-dependent protein kinase stimulates an independently active, nonhomologous, end-joining apparatus. Cancer Res. *60*, 1245–1253.

Duda, D.M., Borg, L.A., Scott, D.C., Hunt, H.W., Hammel, M., and Schulman, B.A. (2008). Structural insights into NEDD8 activation of cullin-RING ligases: conformational control of conjugation. Cell *134*, 995–1006.

Enchev, R.I., Schulman, B.A., and Peter, M. (2015). Protein neddylation: beyond cullin-RING ligases. Nat. Rev. Mol. Cell Biol. *16*, 30–44.

Feng, L., and Chen, J. (2012). The E3 ligase RNF8 regulates KU80 removal and NHEJ repair. Nat. Struct. Mol. Biol. *19*, 201–206.

Frit, P., Li, R.Y., Arzel, D., Salles, B., and Calsou, P. (2000). Ku entry into DNA inhibits inward DNA transactions in vitro. J. Biol. Chem. *275*, 35684–35691.

Galanty, Y., Belotserkovskaya, R., Coates, J., and Jackson, S.P. (2012). RNF4, a SUMO-targeted ubiquitin E3 ligase, promotes DNA double-strand break repair. Genes Dev. *26*, 1179–1195.

Garcia, K., Blank, J.L., Bouck, D.C., Liu, X.J., Sappal, D.S., Hather, G., Cosmopoulos, K., Thomas, M.P., Kuranda, M., Pickard, M.D., et al. (2014). Nedd8-activating enzyme inhibitor MLN4924 provides synergy with mitomycin C through interactions with ATR, BRCA1/BRCA2, and chromatin dynamics pathways. Mol. Cancer Ther. *13*, 1625–1635.

Groisman, R., Polanowska, J., Kuraoka, I., Sawada, J., Saijo, M., Drapkin, R., Kisselev, A.F., Tanaka, K., and Nakatani, Y. (2003). The ubiquitin ligase activity in the DDB2 and CSA complexes is differentially regulated by the COP9 signalosome in response to DNA damage. Cell *113*, 357–367.

Grundy, G.J., Rulten, S.L., Zeng, Z., Arribas-Bosacoma, R., Iles, N., Manley, K., Oliver, A., and Caldecott, K.W. (2013). APLF promotes the assembly and activity of non-homologous end joining protein complexes. EMBO J. *32*, 112–125.

Grundy, G.J., Moulding, H.A., Caldecott, K.W., and Rulten, S.L. (2014). One ring to bring them all—the role of Ku in mammalian non-homologous end joining. DNA Repair (Amst.) *17*, 30–38.

Hjerpe, R., Thomas, Y., and Kurz, T. (2012). NEDD8 overexpression results in neddylation of ubiquitin substrates by the ubiquitin pathway. J. Mol. Biol. *421*, 27–29.

Huang, D.T., Ayrault, O., Hunt, H.W., Taherbhoy, A.M., Duda, D.M., Scott, D.C., Borg, L.A., Neale, G., Murray, P.J., Roussel, M.F., and Schulman, B.A. (2009). E2-RING expansion of the NEDD8 cascade confers specificity to cullin modification. Mol. Cell *33*, 483–495.

Jackson, S.P., and Bartek, J. (2009). The DNA-damage response in human biology and disease. Nature *461*, 1071–1078.

Jackson, S.P., and Durocher, D. (2013). Regulation of DNA damage responses by ubiquitin and SUMO. Mol. Cell *49*, 795–807.

Jimeno, S., Fernández-Ávila, M.J., Cruz-García, A., Cepeda-García, C., Gómez-Cabello, D., and Huertas, P. (2015). Neddylation inhibits CtIP-mediated resection and regulates DNA double strand break repair pathway choice. Nucleic Acids Res. *43*, 987–999.

Kee, Y., Huang, M., Chang, S., Moreau, L.A., Park, E., Smith, P.G., and D'Andrea, A.D. (2012). Inhibition of the Nedd8 system sensitizes cells to DNA interstrand cross-linking agents. Mol. Cancer Res. *10*, 369–377.

Kurz, T., Ozlü, N., Rudolf, F., O'Rourke, S.M., Luke, B., Hofmann, K., Hyman, A.A., Bowerman, B., and Peter, M. (2005). The conserved protein DCN-1/Dcn1p is required for cullin neddylation in C. elegans and S. cerevisiae. Nature *435*, 1257–1261.

Kurz, T., Chou, Y.C., Willems, A.R., Meyer-Schaller, N., Hecht, M.L., Tyers, M., Peter, M., and Sicheri, F. (2008). Dcn1 functions as a scaffold-type E3 ligase for cullin neddylation. Mol. Cell *29*, 23–35.

Li, T., Guan, J., Huang, Z., Hu, X., and Zheng, X. (2014). RNF168-mediated H2A neddylation antagonizes ubiquitylation of H2A and regulates DNA damage repair. J. Cell Sci. *127*, 2238–2248.

Löbrich, M., Shibata, A., Beucher, A., Fisher, A., Ensminger, M., Goodarzi, A.A., Barton, O., and Jeggo, P.A. (2010). gammaH2AX foci analysis for monitoring DNA double-strand break repair: strengths, limitations and optimization. Cell Cycle *9*, 662–669.

Lukas, C., Falck, J., Bartkova, J., Bartek, J., and Lukas, J. (2003). Distinct spatiotemporal dynamics of mammalian checkpoint regulators induced by DNA damage. Nat. Cell Biol. *5*, 255–260.

Lydeard, J.R., Schulman, B.A., and Harper, J.W. (2013). Building and remodelling Cullin-RING E3 ubiquitin ligases. EMBO Rep. *14*, 1050–1061.

Ma, T., Chen, Y., Zhang, F., Yang, C.Y., Wang, S., and Yu, X. (2013). RNF111-dependent neddylation activates DNA damage-induced ubiquitination. Mol. Cell *49*, 897–907.

Meerang, M., Ritz, D., Paliwal, S., Garajova, Z., Bosshard, M., Mailand, N., Janscak, P., Hübscher, U., Meyer, H., and Ramadan, K. (2011). The

ubiquitin-selective segregase VCP/p97 orchestrates the response to DNA double-strand breaks. Nat. Cell Biol. *13*, 1376–1382.

Meyer-Schaller, N., Chou, Y.C., Sumara, I., Martin, D.D., Kurz, T., Katheder, N., Hofmann, K., Berthiaume, L.G., Sicheri, F., and Peter, M. (2009). The human Dcn1-like protein DCNL3 promotes Cul3 neddylation at membranes. Proc. Natl. Acad. Sci. USA *106*, 12365–12370.

Milhollen, M.A., Narayanan, U., Soucy, T.A., Veiby, P.O., Smith, P.G., and Amidon, B. (2011). Inhibition of NEDD8-activating enzyme induces rereplication and apoptosis in human tumor cells consistent with deregulating CDT1 turnover. Cancer Res. *71*, 3042–3051.

Milhollen, M.A., Thomas, M.P., Narayanan, U., Traore, T., Riceberg, J., Amidon, B.S., Bence, N.F., Bolen, J.B., Brownell, J., Dick, L.R., et al. (2012). Treatment-emergent mutations in NAEβ confer resistance to the NEDD8-activating enzyme inhibitor MLN4924. Cancer Cell *21*, 388–401.

Ochi, T., Blackford, A.N., Coates, J., Jhujh, S., Mehmood, S., Tamura, N., Travers, J., Wu, Q., Draviam, V.M., Robinson, C.V., et al. (2015). DNA repair. PAXX, a paralog of XRCC4 and XLF, interacts with Ku to promote DNA double-strand break repair. Science *347*, 185–188.

Ono, M., Tucker, P.W., and Capra, J.D. (1996). Ku is a general inhibitor of DNA-protein complex formation and transcription. Mol. Immunol. *33*, 787–796.

Pinder, J.B., Attwood, K.M., and Dellaire, G. (2013). Reading, writing, and repair: the role of ubiquitin and the ubiquitin-like proteins in DNA damage signaling and repair. Front. Genet. *4*, 45.

Polo, S.E., and Jackson, S.P. (2011). Dynamics of DNA damage response proteins at DNA breaks: a focus on protein modifications. Genes Dev. *25*, 409–433.

Postow, L. (2011). Destroying the ring: Freeing DNA from Ku with ubiquitin. FEBS Lett. *585*, 2876–2882.

Postow, L., and Funabiki, H. (2013). An SCF complex containing Fbxl12 mediates DNA damage-induced Ku80 ubiquitylation. Cell Cycle *12*, 587–595.

Postow, L., Ghenoiu, C., Woo, E.M., Krutchinsky, A.N., Chait, B.T., and Funabiki, H. (2008). Ku80 removal from DNA through double strand break-induced ubiquitylation. J. Cell Biol. *182*, 467–479.

Poulsen, S.L., Hansen, R.K., Wagner, S.A., van Cuijk, L., van Belle, G.J., Streicher, W., Wikström, M., Choudhary, C., Houtsmuller, A.B., Marteijn, J.A., et al. (2013). RNF111/Arkadia is a SUMO-targeted ubiquitin ligase that facilitates the DNA damage response. J. Cell Biol. *201*, 797–807.

Rogakou, E.P., Pilch, D.R., Orr, A.H., Ivanova, V.S., and Bonner, W.M. (1998). DNA double-stranded breaks induce histone H2AX phosphorylation on serine 139. J. Biol. Chem. *273*, 5858–5868.

Sarikas, A., Hartmann, T., and Pan, Z.-Q. (2011). The cullin protein family. Genome Biol. *12*, 220.

Schulman, B.A., and Harper, J.W. (2009). Ubiquitin-like protein activation by E1 enzymes: the apex for downstream signalling pathways. Nat. Rev. Mol. Cell Biol. *10*, 319–331.

Scott, D.C., Monda, J.K., Grace, C.R., Duda, D.M., Kriwacki, R.W., Kurz, T., and Schulman, B.A. (2010). A dual E3 mechanism for Rub1 ligation to Cdc53. Mol. Cell *39*, 784–796.

Shibata, A., Conrad, S., Birraux, J., Geuting, V., Barton, O., Ismail, A., Kakarougkas, A., Meek, K., Taucher-Scholz, G., Löbrich, M., and Jeggo, P.A. (2011). Factors determining DNA double-strand break repair pathway choice in G2 phase. EMBO J. *30*, 1079–1092.

Soucy, T.A., Smith, P.G., Milhollen, M.A., Berger, A.J., Gavin, J.M., Adhikari, S., Brownell, J.E., Burke, K.E., Cardin, D.P., Critchley, S., et al. (2009). An inhibitor of NEDD8-activating enzyme as a new approach to treat cancer. Nature *458*, 732–736.

Toth, J.I., Yang, L., Dahl, R., and Petroski, M.D. (2012). A gatekeeper residue for NEDD8-activating enzyme inhibition by MLN4924. Cell Rep. *1*, 309–316.

Walker, J.R., Corpina, R.A., and Goldberg, J. (2001). Structure of the Ku heterodimer bound to DNA and its implications for double-strand break repair. Nature *412*, 607–614.

Wang, C., and Lees-Miller, S.P. (2013). Detection and repair of ionizing radiation-induced DNA double strand breaks: new developments in nonhomologous end joining. Int. J. Radiat. Oncol. Biol. Phys. *86*, 440–449.

Wang, H., Zeng, Z.C., Perrault, A.R., Cheng, X., Qin, W., and Iliakis, G. (2001). Genetic evidence for the involvement of DNA ligase IV in the DNA-PK-dependent pathway of non-homologous end joining in mammalian cells. Nucleic Acids Res. *29*, 1653–1660.

Watson, I.R., Irwin, M.S., and Ohh, M. (2011). NEDD8 pathways in cancer, Sine Quibus Non. Cancer Cell *19*, 168–176.

Wei, D., Li, H., Yu, J., Sebolt, J.T., Zhao, L., Lawrence, T.S., Smith, P.G., Morgan, M.A., and Sun, Y. (2012). Radiosensitization of human pancreatic cancer cells by MLN4924, an investigational NEDD8-activating enzyme inhibitor. Cancer Res. *72*, 282–293.

Wu, J., Zhang, X., Zhang, L., Wu, C.Y., Rezaeian, A.H., Chan, C.H., Li, J.M., Wang, J., Gao, Y., Han, F., et al. (2012). Skp2 E3 ligase integrates ATM activation and homologous recombination repair by ubiquitinating NBS1. Mol. Cell *46*, 351–361.

Xing, M., Yang, M., Huo, W., Feng, F., Wei, L., Jiang, W., Ning, S., Yan, Z., Li, W., Wang, Q., et al. (2015). Interactome analysis identifies a new paralogue of XRCC4 in non-homologous end joining DNA repair pathway. Nat. Commun. *6*, 6233.

Xirodimas, D.P., Saville, M.K., Bourdon, J.C., Hay, R.T., and Lane, D.P. (2004). Mdm2-mediated NEDD8 conjugation of p53 inhibits its transcriptional activity. Cell *118*, 83–97.

Yang, D., Tan, M., Wang, G., and Sun, Y. (2012). The p21-dependent radiosensitization of human breast cancer cells by MLN4924, an investigational inhibitor of NEDD8 activating enzyme. PLoS ONE *7*, e34079.

Cell Reports
Supplemental Information

Neddylation Promotes Ubiquitylation and Release of Ku from DNA-Damage Sites

Jessica S. Brown, Natalia Lukashchuk, Matylda Sczaniecka-Clift, Sébastien Britton, Carlos le Sage, Patrick Calsou, Petra Beli, Yaron Galanty, and Stephen P. Jackson

Supplementary Information

Supplemental Figures

Figure S1 (related to Figure 1)

Figure S2 (related to Figure 2)

Figure S3 (related to Figure 3)

Figure S4 (related to Figure 4)

Supplemental Tables

Table S1. Ku70 interactors identified by LC-MS/MS (separate file)

Table S2. Ku ubiquitylation sites identified by LC-MS/MS (separate file)

Table S3. Antibodies used in this study

Table S4. siRNA sequences used in this study

Table S5. Primers used in this study

Supplemental Experimental Procedures

Supplemental References

Supplementary figures

Figure S1 relates to Figure 1.

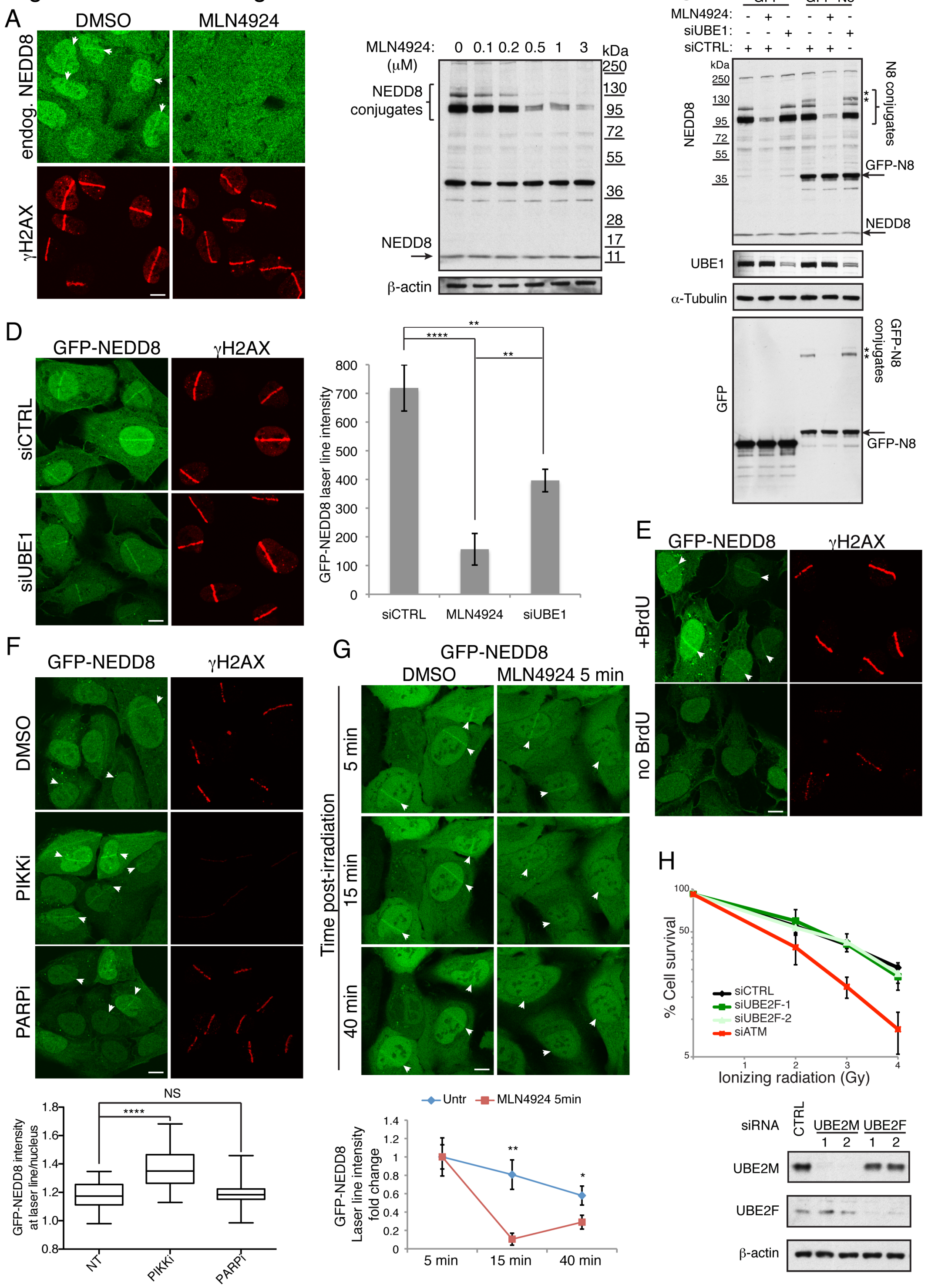

Figure S1 (related to Figure 1).

(A) MLN4924 blocks endogenous NEDD8 recruitment to DNA damage sites. U2OS cells were pre-treated for 1 h with DMSO or 3 μM MLN4924 and laser microirradiated. Cells were fixed after 20 min and visualized by immunofluorescence as indicated. White arrows mark sites of endogenous NEDD8 recruitment. White bar = 10 μM.

(B) MLN4924 inhibits neddylation. U2OS cells were treated with increasing doses of MLN4924 (as indicated) for 1 h and whole cell extracts were analyzed by immunoblotting with NEDD8- and β-actin-specific antibodies. Molecular marker is indicated.

(C) GFP-NEDD8 conjugation in a U2OS-GFP-NEDD8 stable cell line is dependent on NEDD8 E1 activity. U2OS-GFP-NEDD8 or U2OS-GFP cells were transfected with the indicated siRNAs and pre-treated for 1 h with 3 μM MLN4924 where indicated. Whole cell extracts were analyzed by immunoblotting with the indicated antibodies. The NEDD8 specific antibody recognizes free NEDD8 (~11 kDa) and neddylated substrates (mostly cullins) between 95 and 130 kDa (N8 conjugates). Importantly, GFP-NEDD8 conjugation was abrogated by pre-treatment with MLN4924, but not by depleting the ubiquitin E1 UBE1. Black arrows mark free, GFP- and endogenous NEDD8. Asterisks indicate GFP-neddylated cullins. N8 – NEDD8.

(D) UBE1 depletion reduces GFP-NEDD8 recruitment to DNA damage sites. GFP-NEDD8 cells were transfected with siRNA against UBE1 or control (CTRL) and laser microirradiated. Cells were fixed after 20 min and visualized by immunofluorescence as indicated. Quantification shows the average intensity of GFP-NEDD8 signal at the laser line (see supplementary experimental procedures

for details). Data are from the same experiment as Figure 1B. Error bars represent standard deviation between three independent experiments (asterisks as Figure 1B). White bar = 10 μM.

(E) GFP-NEDD8 is recruited to DSBs. U2OS cells stably expressing GFP-NEDD8 were untreated or incubated with 10 μM BrdU for 24 h prior to microirradiation. Cells were fixed after 20 min and visualized by immunofluorescence. White bar = 10 μM.

(F) GFP-NEDD8 recruitment is not dependent on the DDR PI-3-Kinases or PARP activity. U2OS-GFP-NEDD8 cells were pre-treated for 1 h with 10 μM ATM inhibitor, 1 μM ATR inhibitor and 3 μM DNA-PK inhibitor (labeled PIKKi), with 10 μM PARP inhibitor (PARPi) or with DMSO and microirradiated at 200 μW laser power. Cells were fixed after 20 min and visualized by immunofluorescence. White arrows mark sites of GFP-NEDD8 recruitment. Quantification shows fold change in GFP-NEDD8 intensity at the laser line compared to background of a representative experiment. Graph shows median intensity values with inter-quartile range. P-values were calculated using the non-parametric Mann-Whitney test (asterisks as Figure 1B). White bar = 10 μM.

(G) GFP-NEDD8 recruitment to sites of damage is a dynamic process. Live cell imaging of U2OS cells stably expressing GFP-NEDD8 pre-treated for 5 min with DMSO or 3 μM MLN4924 and laser microirradiated. White arrows mark sites of GFP-NEDD8 recruitment. Graph shows fold change of GFP-NEDD8 intensity at laser lines from 5 min time point. Error bars represent standard deviation between three independent experiments (asterisks as Figure 1B). White bar = 10 μM.

(H) Depletion of UBE2F does not cause cellular hypersensitivity to IR. Clonogenic cell survivals were performed in U2OS cells transfected with the indicated siRNA and subjected to increasing doses of IR. Immunoblot shows depletion of UBE2M and UBE2F. As UBE2F and UBE2M co-depletion was highly toxic, we were unable to test the effects of their combined depletion on cell survival after IR. Each point represents an average of at least three independent experiments. Error bars correspond to standard deviations between experiments (asterisks as Figure 1B).

Figure S2 relates to Figure 2.

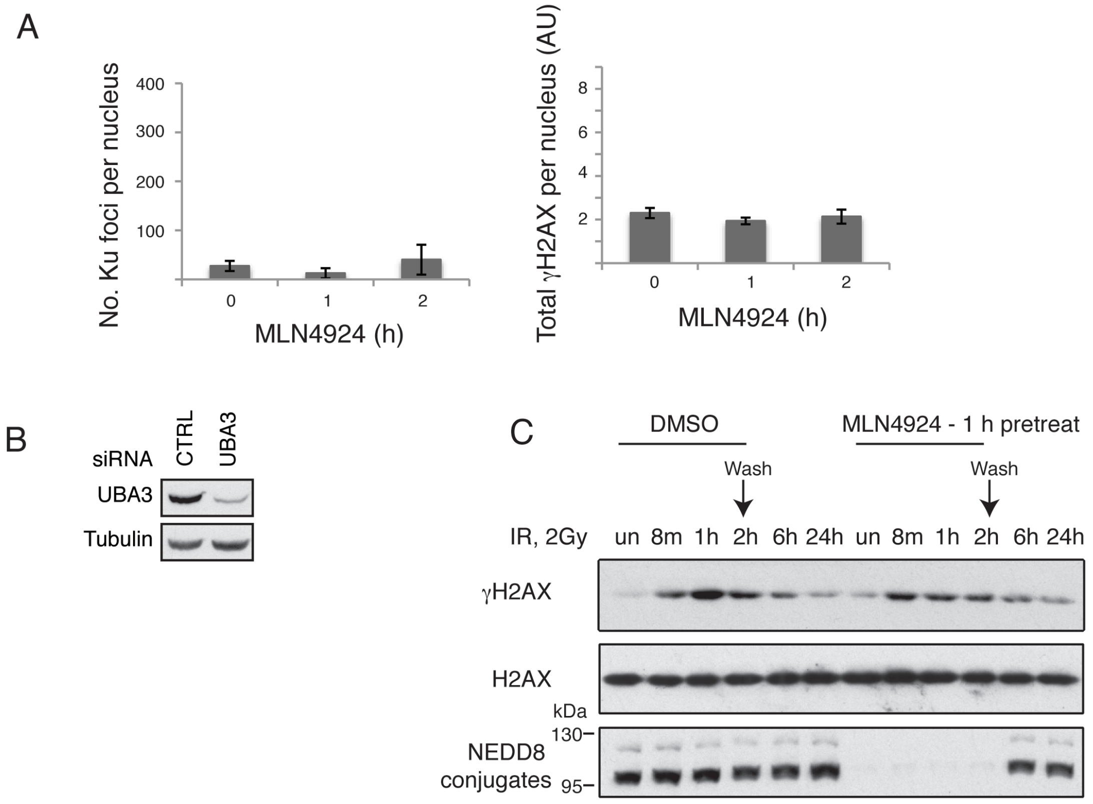

Figure S2 (related to Figure 2).

(A) MLN4924 does not affect the number of Ku80 foci (left panel) or total γH2AX intensity (right panel) per nucleus in undamaged cells. U2OS cells were treated with 3 μM MLN4924 for the indicated time points and analyzed by immunofluorescence. The graphs show the average of at least two independent experiments -/+ standard deviation (AU, arbitrary units).

(B) Immunoblot showing siRNA depletion of endogenous UBA3 in U2OS cells.

(C) Short treatment with MLN4924 does not affect γH2AX recovery following IR. U2OS cells were treated with 3 μM MLN4924 or DMSO for 1 h prior to treatment with 2 Gy IR. Cells were lysed at indicated time points after IR. MLN4924 was washed off 2 h following DNA damage (to prevent induction of DNA damage by MLN4924). Cell lysates were subject to immunoblotting with the indicated

antibodies. Note that neddylation is inhibited by MLN4924 but recovers 4 h after wash-off.

Figure S3 relates to Figure 3.

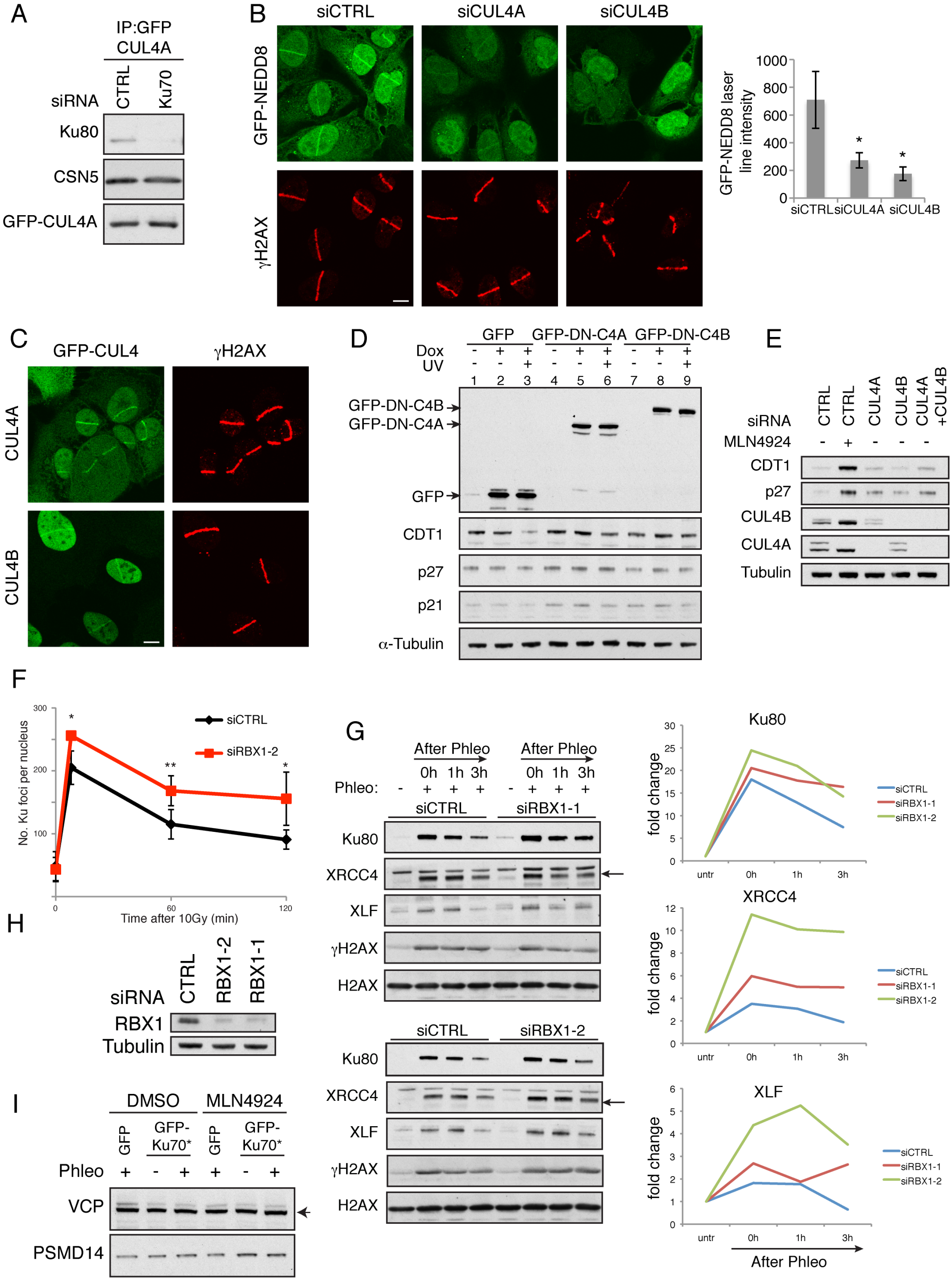

* Endogenously tagged

Figure S3 (related to Figure 3)

(A) GFP-CUL4A interacts with Ku80. U2OS cells stably expressing GFP-CUL4A were transfected with siRNA against Ku70 or control (CTRL). Cells were treated for 1 h with 500 μM Phleomycin. Cell lysates were immunoprecipitated with GFP-Trap beads and immunoblotted with the indicated antibodies. Depletion of Ku70 causes instability and decreases the levels of Ku80 (Britton et al., 2013),

(B) Depletion of CUL4A or CUL4B impairs NEDD8 recruitment to DSB sites. U2OS-GFP-NEDD8 cells were transfected with siCTRL, siCUL4A or siCUL4B (pool of 2 siRNAs) and subjected to laser microirradiation. Cells were fixed after 20 min and stained with γH2AX antibody. Quantification shows the average intensity of GFP-NEDD8 signal at the laser line (see Supplementary Experimental procedures for details) from three independent experiments. Error bars represent standard deviation between experiments (asterisks as Figure 1B). White bar = 10 μM.

(C) GFP-CUL4A and GFP-CUL4B are recruited to DNA damage sites. U2OS cells stably expressing GFP-CUL4B or inducible GFP-CUL4A were subjected to laser microirradiation. Cells were fixed after 20 min and stained with γH2AX antibody. White bar = 10 μM.

(D) Cell lines stably expressing DN-CUL4A or DN-CUL4B do not inhibit CRL4 activity efficiently. U2OS cell lines stably expressing inducible GFP, DN-GFP-CUL4A (C4A) or DN-GFP-CUL4B (C4B) were generated. GFP, DN-GFP-CUL4A or DN-GFP-CUL4B expression was induced with Doxycycline (Dox) as indicated 24 h prior to cell lysis (lanes 2+3, 5+6, 8+9). Cells were treated with 10 J/m^2 UV where indicated and collected 1 h later. CDT1 is degraded after UV in cells expressing GFP only (lanes 2 and 3). Expression of DN-CUL4A or DN-CUL4B

stabilizes CDT1 levels after UV damage as previously described (lanes 5 and 6; 8 and 9; (Emanuele et al., 2011). Basal levels of CDT1 and p21 were unaffected upon expression of DN-CUL4A and DN-CUL4B (lanes, 4 and 5, 7 and 8) and basal levels of p27 were marginally stabilized following DN-CUL4B (lanes 7 and 8), but not DN-CUL4A (lanes 4 and 5) expression.

(E) siRNA depletion of CUL4A or CUL4B fails to efficiently inhibit CRL4 activity. CUL4A and CUL4B were depleted using the indicated siRNAs (siCUL4A-2 or siCUL4B-1) either alone or in combination. Where indicated, 1 μM MLN4924 was added 16 h prior to cell lysis. Cell lysates were subject to immunoblotting with the indicated antibodies. MLN4924 treatment caused marked stabilization of CDT1 and p27 representing effective CRL4 inhibition (Emanuele et al., 2011; Higa et al., 2006). siRNA depletion of CUL4A had marginal effects on CDT1 and p27 stabilization compared to MLN4924.

(F) RBX1 depletion increases Ku foci numbers following DNA damage. U2OS cells were transfected with siRNA against RBX1 or control (siCTRL) and then treated with 10 Gy IR. Cells were collected and analyzed as Figure 2A+B. The higher number of Ku foci detected at 8 min in the siRBX1 sample likely reflects a failure to release Ku at this early time-point compared to control cells.

(G) RBX1 depletion increases retention of Ku and NHEJ factors on the chromatin following DNA damage. U2OS cells were transfected with two independent siRNAs against RBX1 or control (siCTRL) and then treated with 500 μM Phleomycin for 1 h. Samples were collected as in Figure 3A. Intensity of bands was quantified using Fiji software and intensities for Ku80, XRCC4 and XLF were normalized to intensity of H2AX. Graphs show fold change from the untreated sample.

(H) Immunoblot showing siRNA depletion of RBX1 in U2OS cells.

(I) MLN4924 treatment does not affect levels of VCP or PSMD14. RPE1 cells expressing GFP or endogenously tagged with GFP-Ku70 were treated with 3 μM MLN4924 or DMSO for 1 h, followed by 500 μM Phleomycin for 1 h as indicated. Whole cell extracts were immunoblotted with the indicated antibodies. Black arrow indicates VCP.

Figure S4 relates to Figure 4.

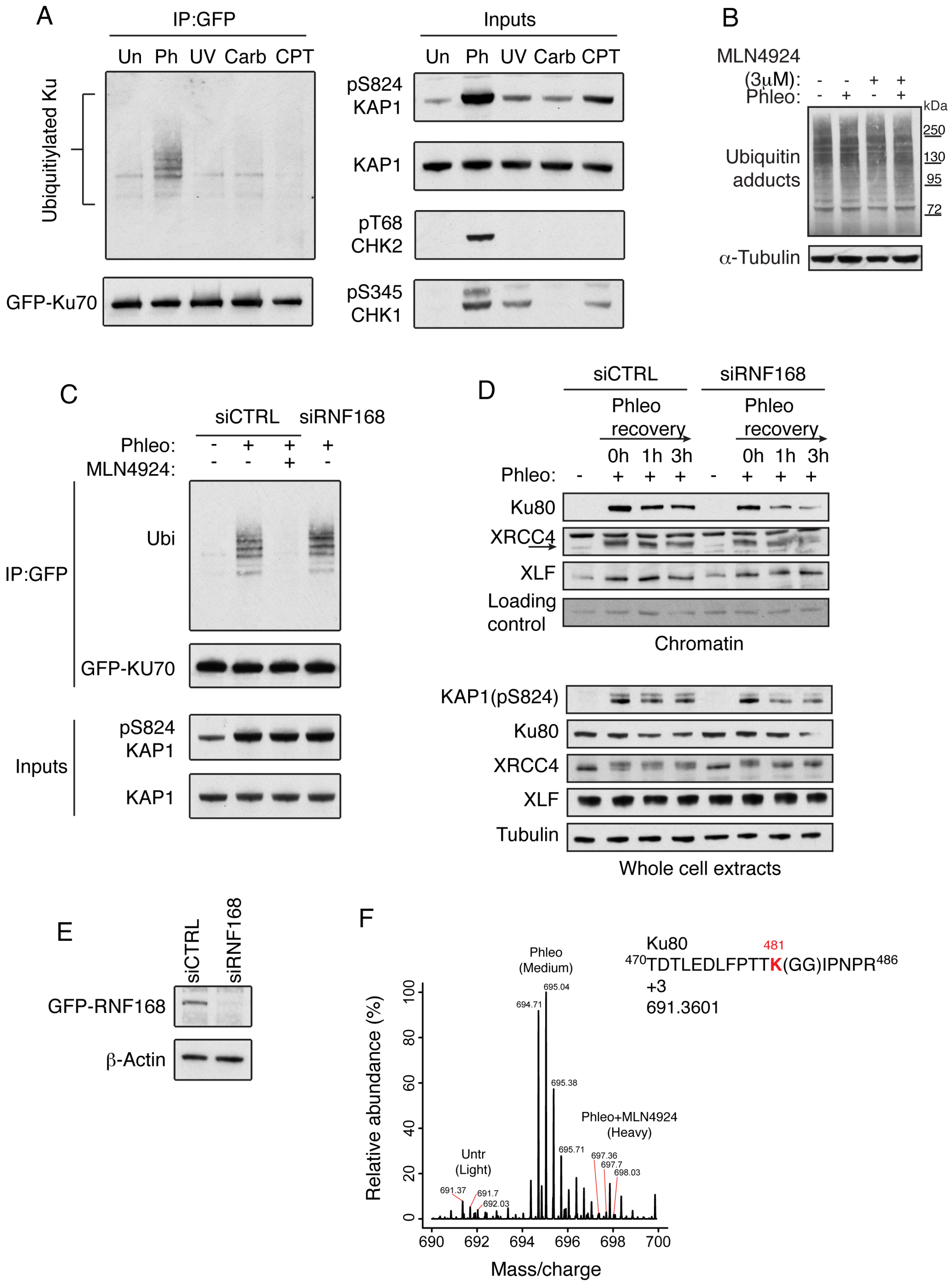

Figure S4 (related to Figure 4)

(A) Ku ubiquitylation occurs after Phleomycin, which induces DSBs directly. *In vivo* ubiquitylation assay was performed as in Figure 4A in RPE-GFP-Ku70 cells untreated (Un) or treated with 500 μM Phleomycin (Ph), 10J/m^2 UV, 100 μM Carboplatin (Carb), 1 μM Camptothecin (CPT), and collected 1h after.

(B) MLN4924 does not affect overall cellular ubiquitylation detected by immunoblotting. U2OS cells were pre-treated with DMSO or 3 μM MLN4924 for 1 h, followed by treatment with 500 μM Phleomycin for 1 h. Cell lysates were subject to immunoblotting with the Ubiquitin- or α-Tubulin-specific antibodies. Molecular marker is indicated.

(C) RNF168 depletion does not affect Ku ubiquitylation following DNA damage. *In vivo* ubiquitylation assay was performed as in Figure 4A in RPE-GFP-Ku70 cells transfected with siRNA against RNF168 or control (siCTRL) and treated with 500 μM Phleomycin (Phleo) for 1h. As a positive control, siCTRL cells were also pretreated with 3 μM MLN4924 for 1h prior to Phleomycin treatment. Ubi – ubiquitination recognized by anti-Ubiquitin antibody.

(D) RNF168 depletion does not cause persistence of Ku on the chromatin following DNA damage. U2OS cells were transfected with siRNA against RNF168 or control (siCTRL). Cells were treated with Phleomycin as indicated and the assay was performed as in Figure 3A. A non-specific antibody band is used as a loading control.

(E) Immunoblot showing siRNA depletion of GFP-RNF168 in U2OS cells stably expressing GFP-RNF168 transfected with siCTRL or siRNF168.

(F) DNA damage-induced ubiquitylation of Ku80 on Lys 481 is inhibited by MLN4924 treatment. The mass spectrum shows the relative abundance of the Ku80 peptide TDTLEDLFPTTK(GG)IPNPR (aa470-486) in untreated (SILAC light) and phleomycin-treated cells without (SILAC medium) and with MLN4924 pre-treatment (SILAC heavy). The peptide sequence, mass/charge (m/z) and charge state are indicated.

Supplementary tables

Table S3. Antibodies used in this study.

Target	Mono/ polyclonal	Reference	Raised in	Source	Dilution for I.B	Dilution for I.F
α–Tubulin	Monoclonal	T9026	Mouse	Sigma	1:20 000	
β-Actin	Monoclonal	ab8226	Mouse	Abcam	1:5000	
CHK1pS345	Polyclonal	2348	Rabbit	Cell signaling	1:5000	
CHK2pT68	Polyclonal	2661S	Rabbit	Cell signaling	1:1000	
CDT1	Polyclonal	ab70829	Rabbit	Abcam	1:1000	
CSN5	Polyclonal	A300-014A	Rabbit	Bethyl	1:5000	
CUL1	Monoclonal	ab7581	Rabbit	Abcam	1:1000	
CUL4A	Monoclonal	ab92554	Rabbit	Abcam	1:20000	
CUL4B	Monoclonal	ab76470	Rabbit	Abcam	1:1000	
FLAG	Monoclonal	F3165	Mouse	Sigma	1:1000	
GFP	Monoclonal	11814460001	Mouse	Roche	1:1000	
LIG4	Polyclonal	(Riballo et al., 1999)	Rabbit		1:1000	
γH2AX	Monoclonal	05-636	Mouse	Millipore	1:1000	1:250
γH2AX	Polyclonal	2577	Rabbit	Cell signaling		1:250
H2AX	Polyclonal	ab11175	Rabbit	Abcam	1:5000	
Histone H1	Polyclonal	ab1938	Sheep	Abcam	1:1000	
KAP1 (pS824)	Polyclonal	IHC-00073	Rabbit	Bethyl	1:1000	
KAP1	Polyclonal	ab10483	Rabbit	Abcam	1:2000	
Ku70	Monoclonal	Ab3114	Mouse	Abcam	1:200	
KU80	Monoclonal	LVMS285P1	Mouse	Fisher Scientific	1:2000	1:100
NEDD8	Monoclonal	1571-1	Rabbit	Epitomics	1:5000	1:250
P21	Polyclonal	Sc397	Rabbit	Santa Cruz	1:1000	
P27	Monoclonal	610241	Mouse	BD Bioscience	1:2000	
PSMD14	Monoclonal	ab109123	Rabbit	Abcam	1:1000	
UBA3	Monoclonal	ab124728	Rabbit	Abcam	1:10 000	
UBE1	Monoclonal	ab34711	Rabbit	Abcam	1:1000	
UBE2F	Polyclonal	ab15707	Goat	Abcam	1:1000	
UBE2M	Monoclonal	ab109507	Rabbit	Abcam	1:10 000	
Total Ubiquitin	Polyclonal	3933	Rabbit	Cell signaling	1:1000	
Ubiquitin (FK2)	Monoclonal	PW8810	Mouse	Enzo Life Sciences	1:1000	
VCP	Monoclonal	612183	Mouse	BD Bioscience	1:1000	
XLF	Polyclonal	ab33499	Rabbit	Abcam	1:500	
XRCC4	Polyclonal	ab145	Rabbit	Abcam	1:2000	

Table S4. siRNA sequences used in this study

Target	Target sequence	CDS/UTR
ATM	GAC UUU GGC UGU CAA CUU UCG	CDS
CTRL (Luciferase)	CGU ACG CGG AAU ACU UCG A	CDS
CUL4A-1	GAA GAU UAA CAC GUG CUG G	CDS
CUL4A-2	GCU UAG AGG AAG AGG GAG A	CDS
CUL4B-1	CAG AAU UUA AAG AGG GUA A	CDS
CUL4B-2	GGA ACA UCA UAG AAG AGA A	3' UTR
Ku70	GAG UGA AGA UGA GUU GAC A	CDS
RBX1-1	GGG AUA UUG UGG UUG AUA A	CDS
RBX1-2	CCA UUG GAC AAC AGA GAG U	CDS
RNF168	GGC GAA GAG CGA UGG AGG A	CDS
UBA3	AGA GAG AGA UUA UGA GCA A	3' UTR
UBE1	CGU CAG ACC UGC AAG AGA A	CDS
UBE2F-1	GGA AUA AAG UGG AUG ACU A	CDS
UBE2F-2	CAA CAU AAA UAC AGC AAG A	3' UTR
UBE2M-1	AGC CAG UCC UUA CGA UAA A	CDS
UBE2M-2	GAU GAG GGC UUC UAC AAG A	CDS
XRCC4	AUA UGU UGG UGA ACU GAG A	CDS

Table S5. Primers used in this study

ID	Sequence	Restriction site
CUL4A-F	CT AAA GCT TCT GCG GAC GAG GCC CCG CGG AAG	HindIII
CUL4A-R	CTA GGA TCC TCA GGC CAC GTA GTG GTA CTG	BamHI
DN-CUL4A-F	CTC AAG CTT CCG CGG ACG AGG CCC C	HindIII
DN-CUL4A-R	TAG AAT TCC TAC TTG TCA TCG TCA TCC TTG TAG	EcoRI
DN-CUL4B-F	CTC AAG CTT CCA TGT CAC AGT CAT CTG GAT CA	HindIII
DN-CUL4B-R	GTA GGA TCC CTA CTT GTC ATC GTC ATC CTT GTA G	BamHI
NEDD8-F	AAG GCT CGA GCT CTA ATT AAA GTG AAG ACG CTG ACC	XhoI
NEDD8-R	GGA TCC CTA TCC TCC TCT CAG AGC CAA CAC	BamHI
UBE2F-F	TACT CGA GTG CTA ACG CTA GCA AGT AAA C	XhoI
UBE2F-R	TA GGA TCC TCT GGC ATA ACG TTT GAT G	BamHI
UBE2M-F	TAGT CGA CTG ATC AAG CTG TTC TCG CTG	SalI
UBE2M-R	TA AGA TCT TTT CAG GCA GCG CTC AAA GTA	BglII

Supplementary Experimental Procedures.

Cell Culture

Cells were grown in a 5% CO_2 humidified incubator at 37°C. U2OS cells were grown in DMEM supplemented with 10% FBS, 2 mM L-glutamine, 100 U/ml penicillin, and 100 μg/ml streptomycin. RPE-1 - human telomerase reverse transcriptase (hTERT) expressing cells were grown in DMEM/Ham's F12 medium supplemented as previously and buffered with sodium bicarbonate. U2OS cells stably expressing GFP, GFP-NEDD8, GFP-UBE2M, GFP-UBE2F, GFP-CSN5, GFP-CUL4B, GFP-RNF168, FLAG-UBA3 WT and FLAG-UBA3 A171T and RPE-1 cells stably expressing GFP were grown in medium supplemented with 0.5 mg/ml G418 (GIBCO, Life Technologies). U2OS cells stably expressing doxycycline-inducible GFP-CUL4A were grown in medium supplemented with 0.5 mg/ml G418 (GIBCO, Life Technologies) and 2 μg/ml Blasticidin (Invitrogen), and 10% Tet-negative FBS. U2OS cells stably expressing doxycycline-inducible DN-GFP-CUL4A or DN-GFP-CUL4B were grown in medium supplemented with 0.25 mg/ml G418 and 10% Tet-negative FBS.

siRNA transfections

siRNA transfections were done using Lipofectamine RNAiMAX (Life Technologies) according to manufacturers instructions. Two-rounds of transfection were performed 24 h apart for optimal depletion of the target protein. Experiments were performed 72 h after the 1st transfection, 144 h for siRNA Ku70. siRNA duplexes were purchased from MWG Biotech and the

sequences used are listed in Table S4. A sequence against firefly luciferase was used as siCTRL.

Plasmids.

For a list of all primers used in this study, see Table S5. All constructs were fully sequenced and are mutation free. NEDD8 was PCR-amplified from a human fetal brain cDNA library and cloned into pEGFP-C1. The original mammalian constructs of UBE2M and UBE2F were provided by the Division of Signal Transduction Therapy, Department of Biochemistry, Medical Sciences Institute, University of Dundee. They were PCR amplified and cloned into pEGFP-C1 vectors. The original constructs expressing GFP-tagged human CUL4A and CUL4B were provided by Prof. Nico Dantuma (Karolinska Institute, Sweden) and Prof. Changshun Shao (Rutgers University, US), respectively. CUL4A was PCR amplified with CUL4A-F and CUL4A-R primers and cloned into pEGFP-TO-C1 (TO – Tet-operator). GFP-DN-CUL4A-FLAG and GFP-DN-CUL4B-FLAG plasmids for inducible expression were constructed by subcloning pcDNA3-DN-hCUL4A-FLAG or pcDNA3-DN-hCUL4B-FLAG (Addgene) into the pEGFP-TO-C1 vector by PCR, using DN-CUL4A-F, DN-CUL4A-R, DN-CUL4B-F or DN-CUL4B-R primers. Sylvie Urbe and Michael Clague (Cellular and Molecular Physiology, Institute of Translational Medicine, University of Liverpool, Liverpool, UK) provided the plasmid expressing human GFP-CSN5. The FLAG-UBA3 WT and A171T mutant constructs have been described previously (Toth et al., 2012) and were provided by Matthew Petroski (Sanford-Burnham Medical Research Institute, CA, USA). HA-Ubiquitin has been described previously (Galanty et al., 2012).

Plasmid transfections and stable cell lines

Plasmid transfections were done using TransIT-LT1 (Mirus) according to manufacturers protocol. To generate monoclonal stable cell lines expressing inducible GFP-CUL4A, 48 h after transfection of U2OS TRex (Tet-repressor) cells with pEGFP-TO-CUL4A plasmid, cells were seeded at low density, in the presence of 0.5 mg/ml G418 and 2 μg/ml Blasticidin (Invitrogen). Individual clones were isolated and grown in duplicate in the presence of selection media. Clones were treated with 2 μg/ml doxycycline overnight to induce GFP-CUL4A expression and were selected based on expression of full-length protein, as validated by immunoblotting. To generate polyclonal stable cell lines expressing inducible GFP, GFP-DN-CUL4A or GFP-DN-CUL4B, U2OS TRex cells were transfected with pEGFP-TO-C1, or GFP-DN-CUL4A or GFP-DN-CUL4B using FuGENE HD (Promega) and cultured with 0.5 mg/ml G418 added to the medium for two weeks. Expression was induced by 2 μg/ml doxycycline treatment for 24 h and verified by immunoblotting and microscopy.

To generate all other stable cell lines; 48 h after transfection, cells were seeded at low density, in the presence of 0.5 mg/ml G418 (GIBCO, Life Technologies). Individual clones were isolated, grown in the presence of selection media and again, expression of full-length protein was validated by immunoblotting.

U2OS-GFP-RNF168 cells were generated as previously described (Giunta et al., 2010).

Immunoblotting

For whole cell extracts cells were lysed in an SDS lysis buffer (4% SDS, 20% Glycerol, 125 mM Tris-HCl pH 6.8) and protein concentration was measured. Lysates were then diluted to equal concentration and supplemented with 10% β-Mercaptoethanol and 0.005% Bromophenol blue. Samples were resolved on 4-12% gradient Bis-Tris NuPAGE gels (Novex, Life Technologies) and transferred onto nitrocellulose membranes (GE Life Sciences). Membranes were blocked in 5% milk in TBS containing 0.1% Tween-20 and incubated with the corresponding primary antibody (Table S3) followed by an appropriate secondary antibody coupled to horseradish peroxidase (Fisher Scientific). Detection was performed with ECL reagent (GE Healthcare). Quantification of the immunoblots was done using Fiji software (http://fiji.sc/Fiji).

Immunofluorescence

For laser microirradiation experiments, cells were washed three times with PBS and then fixed in 2% PFA (15 min). Cells were permeabilised with 0.2% Triton X-100/PBS (5 min) and then blocked with 5% BSA in PBS/0.1% Tween 20 (PBS-T) for 10 min. Cells were stained for 1 h at room temperature with the indicated primary antibodies in 5% BSA/PBS-T, washed with PBS-T and then stained for 1 h at room temperature with the appropriate goat secondary antibodies coupled to Alexa Fluor 488 or 568 fluorophores in 5% BSA/PBS-T.

Detection of Ku on chromatin

For the detection of Ku on chromatin (Figure 3A) cells were processed similarly to as described previously (Britton et al., 2013). Briefly, cells were pre-extracted twice for 3 min with CSK buffer (10 mM Pipes, pH 7.0, 100 mM NaCl, 300 mM sucrose, 3 mM $MgCl_2$ and 0.7% Triton X-100) supplemented with 0.3mg/ml RNase A for 3 min, then washed 3 times with PBS and collected in a SDS lysis buffer (all at room temperature) and processed for immunoblotting as described above.

Detection of Ku foci

Coverslips were prepared as described previously (Britton et al., 2013). Briefly, cells were seeded onto 160 µm coverslips (VWR International) 24 h prior to the experiment. Cells were washed three times with PBS and then pre-extracted by incubating twice for 3 min in CSK buffer (see Immunoblotting) supplemented with 0.3 mg/ml RNase A and washed three times in between with PBS (all at room temperature). Cells were then fixed in 2% PFA for 15 min. Before staining, cells were treated with PBS/0.2% Triton X-100 for 5 min, washed with PBS-T and then blocked with 5% BSA/PBS-T for 10 min. Cells were incubated in primary antibodies in 5% BSA/PBS-T for 1 h at room temperature, washed in PBS-T and then incubated for 1 h at room temperature with goat secondary antibodies coupled to Alexa Fluor 488 or 594 fluorophores (Life Technologies) in 5% BSA/PBS-T. Cells were mounted onto glass slides using Vectashield (Vector Laboratories).

High resolution microscopy and deconvolution

As described previously for the visualization of Ku foci (Britton et al., 2013), high-resolution images were acquired on a Deltavision PersonalDV (Applied Precision/GE Healthcare) equipped with a 1,024×1,024 CCD camera (CoolSNAP HQ2; Photometrics), and a 100× U Plan S Apochromat/1.40 NA oil objective (Olympus) and controlled with SoftWoRx software x5.5 (Applied Precision/GE Healthcare). Z stacks were taken at 0.2-μm intervals and the fluorescent channels were acquired sequentially. Deconvolution was then performed within SoftWoRx in conservative mode. Brightness and contrast were adjusted and images were cropped using Photoshop CS5 (Adobe).

Quantification of Ku foci

Deconvoluted images of >10 cells per sample were submitted to automatic focus detection using Volocity 6.3 (PerkinElmer). Cells were selected randomly based on DAPI staining. As the DNA content and therefore the number of double strand break induced foci doubles during the S- and G2-phases of the cell cycle, DAPI volume was used as a surrogate marker of DNA content. The number of foci detected per nucleus were therefore adjusted to the mean DAPI volume. Of note, a 1 h, 3 μM treatment of MLN4924 did not significantly alter the mean DAPI volume.

Laser microirradiation

Cells grown on glass-bottom dishes (Willco Wells) in phenol red-free complete medium (Invitrogen) were treated with 10 μM bromodeoxyuridine (BrdU) for 24 h. Laser microirradiation was performed using a FluoView 1000 confocal

inverted microscope (Olympus) equipped with a 37°C heating stage (Ibidi) and a 405 nm laser diode (6 mW) focused through a 60× UPlanSApo/1.35 oil objective to yield a spot size of 0.5-1 mm. The laser settings 0.40 mW output, 50 frames, unless otherwise indicated, were chosen to generate a detectable damage response in a manner dependent on BrdU presensitization and without noticeable cytotoxicity. Cells were analyzed by immunofluorescence using a confocal microscope (FluoView 1000; Olympus).

Quantification of Laser microirradiation experiments

Intensity of GFP-NEDD8 signal in the laser line was calculated by subtracting average nuclear intensity from average laser line intensity (Figures 1B, S1D, S3B and S1G) or by dividing average laser line intensity by average nuclear intensity (Fold change; Figure S1F). Intensity measurements were done using Fiji software (http://fiji.sc/Fiji). P-value was calculated using unpaired T-test (*, P < 0.05; **, P < 0.01; ***, P < 0.001; ****, P≤0.0001).

Random Plasmid Integration assay

Between 2-rounds of siRNA transfection, U2OS cells were transfected with BamHI–XhoI-linearized pEGFP-C1 (Clontech). 24 h after DNA transfection, cells were collected, counted and plated at low confluency onto: a 15 cm dish and 6 cm dish in complete medium, and a 15 cm dish in complete medium with 0.5 mg/ml G418 (GIBCO, Life technologies). The following day, transfection efficiency was calculated by determining the proportion of GFP-positive cells on the 6 cm dish. Cells on the 15 cm dishes were incubated at 37 °C for 10-14 days until colonies had formed. Colonies were stained with 0.5% crystal violet/20%

ethanol and counted. Random plasmid integration events were normalized to transfection and plating efficiencies. The P-value was calculated using an un-paired Student's t-test.

Identification of Ku ubiquitylation sites

RPE-1 cells expressing Ku70 endogenously tagged with GFP were grown in SILAC DMEM/Ham's F12 minus L-Lysine and L-Arginine (Thermo Fisher) supplemented with 10% dialysed FBS, 2 mM L-glutamine, 100 U/ml penicillin, and 100 µg/ml streptomycin, and containing either Lysine (K0)/Arginine (R0) (Light), Lysine (K4)/Arginine (R6) (Medium) or Lysine (K8)/Arginine (R10) (Heavy) amino acids for two weeks prior to the experiment. Light cells were untreated, Medium cells were treated with Phleomycin (500 µM, 1 h) and Heavy cells were treated with MLN4924 (3µM, 1 h) prior to treatment with Phleomycin (500 µM, 1 h). GFP-immunoprecipitation was performed with GFP-TRAP beads (Invitrogen) as described for the Ku ubiquitylation experiment and Ku ubiquitylation was analyzed by MS.

Identification of GFP-Ku70 interactors

RPE-1 cells stably expressing GFP and RPE-1 cells expressing Ku70 endogenously tagged with GFP were grown in SILAC medium as described above for the identification of Ku ubiquitylation sites. RPE-1-GFP were grown in Light and RPE1-GFP-Ku70 were grown in Medium or Heavy media for two weeks prior to the experiment. A full 15 cm plate of cells was treated with Phleomycin (500 µM, 1 h; Light RPE-GFP and Medium RPE-GFP-Ku70) or pre-treated with MLN4924 (3µM, 1 h) prior to Phleomycin treatment (Light RPE-GFP and Heavy

RPE-GFP-Ku70). MLN4924 treatment was added to the GFP-only cells to control for increased non-specific interactions mediated by MLN4924 itself. GFP-immunoprecipitation was performed with GFP-TRAP beads (Invitrogen) similarly as described for the Ku ubiquitylation experiment, with the exception that lysis and washes were done in 150 mM NaCl. Interactors were analyzed by LC-MS/MS.

LC-MS/MS for Ku interactors.

Precipitated proteins were resolved by SDS-PAGE and digested in-gel with trypsin. Peptide fractions were analyzed on a quadrupole Orbitrap mass spectrometer (Q-Exactive Plus, Thermo Scientific) equipped with an EASY-nLC II nanoflow HPLC system (Thermo Scientific) as described (Michalski et al. PMID: 21642640). Raw data files were analyzed using MaxQuant development version 1.3.9.21 (Cox and Mann, 2008). Parent ion and MS2 spectra were searched against a database containing 88,473 human protein sequences obtained from the UniProt knowledge base released in December 2013 using the Andromeda search engine (Cox et al., 2011). Spectra were searched with a mass tolerance of 6 ppm in MS mode, 20 ppm in HCD MS2 mode, strict trypsin specificity and allowing up to 2 missed cleavage sites. Cysteine carbamidomethylation was included as a fixed modification and N-terminal protein acetylation and methionine oxidation were included as variable modifications. For mapping of ubiquitylation sites n-ethylmaleimide modification of cysteines, di-glycine-lysine, N-terminal protein acetylation and methionine oxidation were searched as variable modifications. Site localization probabilities were determined by MaxQuant using the PTM scoring algorithm as described previously (Cox and

Mann, 2008). The dataset was filtered based on posterior error probability (PEP) to arrive at a false discovery rate of 1% for peptide spectrum matches and protein groups.

Supplementary References

Britton, S., Coates, J., and Jackson, S.P. (2013). A new method for high-resolution imaging of Ku foci to decipher mechanisms of DNA double-strand break repair. J. Cell Biol. *202*, 579–595.

Cox, J., and Mann, M. (2008). MaxQuant enables high peptide identification rates, individualized p.p.b.-range mass accuracies and proteome-wide protein quantification. Nat. Biotechnol. *26*, 1367–1372.

Cox, J., Neuhauser, N., Michalski, A., Scheltema, R. a, Olsen, J. V, and Mann, M. (2011). Andromeda: a peptide search engine integrated into the MaxQuant environment. J. Proteome Res. *10*, 1794–1805.

Emanuele, M.J., Elia, A.E.H., Xu, Q., Thoma, C.R., Izhar, L., Leng, Y., Guo, A., Chen, Y.-N., Rush, J., Hsu, P.W.-C., et al. (2011). Global identification of modular cullin-RING ligase substrates. Cell *147*, 459–474.

Galanty, Y., Belotserkovskaya, R., Coates, J., and Jackson, S.P. (2012). RNF4, a SUMO-targeted ubiquitin E3 ligase, promotes DNA double-strand break repair. Genes Dev. *26*, 1179–1195.

Giunta, S., Belotserkovskaya, R., and Jackson, S.P. (2010). DNA damage signaling in response to double-strand breaks during mitosis. J. Cell Biol. *190*, 197–207.

Higa, L., Yang, Y., Zheng, J., Banks, D., Wu, M., Ghosh, P., Sun, H., and Zhang, H. (2006). Involvement of CUL4 Ubiquitin E3 Ligases in Regulating CDK Inhibitors Dacapo/p27KIP1 and Cyclin E Degradation. Cell Cycle 71–77.

Riballo, E., Critchlow, S.E., Teo, S.H., Doherty, a J., Priestley, a, Broughton, B., Kysela, B., Beamish, H., Plowman, N., Arlett, C.F., et al. (1999). Identification of a defect in DNA ligase IV in a radiosensitive leukaemia patient. Curr. Biol. *9*, 699–702.

Toth, J.I., Yang, L., Dahl, R., and Petroski, M.D. (2012). A gatekeeper residue for NEDD8-activating enzyme inhibition by MLN4924. Cell Rep. *1*, 309–316.
